# Supplementary material for: Recent innovations in long-term care coverage and financing: a rapid scoping review
Source: BMJ Open. 2024 Feb 22;14(2):e077309. doi: 10.1136/bmjopen-2023-077309 (PMC10884182; doi:10.1136/bmjopen-2023-077309)
Supplement: Supplementary data [file bmjopen-2023-077309supp002.pdf]

Table 1: Study Characteristics

Author Year	Country	Initiative	Design	Aim	Population and Data Sources	Quality Appraisal
Alders & Shut 2022(54)	The Netherlands	LTCI reform	Economic evaluation	To discern if municipalities with a low solvency rate (<20 percent) are more likely to engage in strategic cost shifting and if this resulted in an overall upward trend in admission rates to LTC services covered by public LTC services.	People aged 65+ in the Netherlands; data from 327 out of 355 municipalities from 2014–2019.<br><br>Data source(s): Monitor Long-term Care (Monitor Langdurige Zorg); Online dashboard by the Ministry of the Interior; Statistics Netherlands	Medium
Beauregard & Miller 2021(47)	U.S.	Community First Choice program	Comparative case study (qualitative)	To understand state processes related to the adoption of the Medicaid Community First Choice (CFC) Program and to identify factors that influenced states' decisions to adopt CFC.	LTC population: Medicaid-eligible older adults in Texas, Oklahoma, Maryland, and nationally (U.S.).<br><br>Study population: Data collected from n=46 individuals in roles as follows: federal and state bureaucrats, consumer and provider advocacy groups, consultants, and policy experts. Maryland (n=14), Texas (n=14), Oklahoma (n=9), and national (n=9) level participants.<br><br>Data source(s): Interviews	Medium
Chen et al. 2020(35)	China	LTCI pilots	Cross-sectional study	To evaluate the influence of health insurance and other factors on the availability of LTC services.	n=7787; Urban: n=3945; Rural: n=3842; CHARLS (2015) database adults aged 45 and up; the sample is aged 60 and up; CHARLS (2015) covered more than 10,000 families and 17,500 individuals from 150 districts of 28 provinces.<br><br>Data source(s): China Health and Retirement Longitudinal Study (CHARLS) database, 2015	Medium
Chen & Fu 2020(53)	Taiwan	LTC 2.0 reform	Policy analysis	To review the development of LTC policies, present strategies for expanding LTC, and outline policy suggestions and implications.	Older adults in Taiwan. The proportion of the population aged 65 years and over in Taiwan has doubled from 7% in 1993 to 14% in 2018. The number of older adults receiving LTC services increased from 9,148 in 2008 to	High

Author Year	Country	Initiative	Design	Aim	Population and Data Sources	Quality Appraisal
					258,351 in 2019, which only accounts for 41.47% of the population needing LTC.<br><br>Data source(s): Policy documents; government data	
Chen & Ning 2022(43)	China	LTCI pilot	Quasi-experimental design	To examine the policy treatment effect of long-term care insurance (LTCI) on health care utilization and OOP health expenditure (OOP) in China.	N=20,814<br>Participants in the China Health and Retirement Longitudinal Study (CHARLS).<br><br>Data source(s): China Health and Retirement Longitudinal Study (CHARLS) longitudinal study, 2018	High
Chiu et al. 2019(52)	Taiwan	LTC 2.0 reform	Qualitative research	To examine the experiences and challenges of integration for LTC plan 2.0 agencies in Taiwan	LTC population: Older adults in 20 counties where LTC Plan 2.0 was being piloted in Taiwan in early 2017.<br><br>Study population: Interviews with 6 people working in 3 LTC Plan 2.0 Tier A agencies in north, west, and central Taiwan, including a chief executive officer (CEO) or executive director (ED) and a case manager at each agency.<br><br>Data source(s): Interviews	Medium
Dai et al. 2022(41)	China	LTCI pilot	Systematic literature review of LTC policies and implementation	This study examines the LTCI pilot in China and compares it to other LTCI programs in Germany, Japan, and South Korea through a systematic literature review and discusses the implications of its implementation in a national framework.	Adults in one of the 49 expanded pilot cities in China.<br><br>Data source(s): National and local government websites; China National Knowledge Infrastructure, Baidu Scholar and international databases	High
Feng et al. 2021(40)	China	LTCI pilot	Policy analysis	To provide a timely review and assessment of China's ongoing LTCI programs	People over 60 who meet the eligibility requirement for the LTCI pilot and who live in one of the 15 pilot cities.	High

Author Year	Country	Initiative	Design	Aim	Population and Data Sources	Quality Appraisal
				across the initial 15 pilot cities. The focus is on synthesizing key LTCI program features across all pilot sites, not on profiling and analyzing individual pilots in depth.	Data source(s): Policy documents; published research articles; grey literature; LTCI Policy Database, School of Public Health, Peking University	
Fong & Borowski 2022(51)	Singapore	CareShield Life	Policy analysis	To analyze the 2019 long-term care social insurance program, including successes and challenges, goals, design, features, financing arrangements, and initial assessment.	Older adults in Singapore. The proportion of those aged 65 and older increased x3 from 4.9% in 1980 to 14.5% in 2019.<br><br>Data source(s): Not reported. The reference list includes government documents and websites.	High
Han & Shen 2022(42)	China	LTCI pilot	Qualitative research	To explore the common problems in policy practices and to provide recommendations for further expansion of the LTCI pilot.	LTC population: China's adult population ≥60, which accounts for 18.7% of the total population.<br><br>Study population: n=10 participants, including managers (n=4), caregivers (n=2), a medical staff member (n=1), and older adults with disabilities (n=3)<br><br>Data source(s): In-depth interviews	High
Hashiguchi & Llena-Nozal 2020(32)	25 out of 41 OECD countries and EU Member States*	Public social protection systems for LTC	Comparative case study	To provide a novel set of comprehensive and internationally comparable estimates of the adequacy, equity and efficiency of public social protection systems for LTC in old age in OECD countries and EU Member States.	People 65 and older in the selected 25 OECD countries and EU Member States*.<br><br>Data source(s): Survey of Health, Ageing and Retirement in Europe (SHARE) Wave 7	N/A

Author Year	Country	Initiative	Design	Aim	Population and Data Sources	Quality Appraisal
Kotschy & Bloom 2022(33)	Germany, Israel, Japan, the Netherlands, South Korea	LTC systems comparison	Policy analysis	To investigate the challenges that population aging poses to long-term care.	Adults aged 65+ in Germany, Israel, Japan, the Netherlands, and South Korea<br><br>Data source(s): Survey of Health, Ageing and Retirement in Europe (SHARE) wave 7 and 8; Health and Retirement Study (HRS) wave 13; English Longitudinal Study of Ageing (ELSA) wave 8	N/A
Koumoutzis et al. 2021(49)	U.S.	Local initiatives to fund social care services	Qualitative research	To examine how communities across the nation are utilizing local funding streams to support aging services.	LTC population: Adults 60+ in the U.S.<br><br>Study population: NR<br><br>Data source(s): 2019 Advancing States national survey of State Units on Aging; telephone calls (State Units on Aging, Area Agencies on Aging, secretary of state's offices, county clerk's offices, and other officials involved in local funding efforts); web searches	Medium
Koumoutzis et al. 2022(48)	U.S.	Community LTC initiatives to support ageing	Survey	To study community initiatives developed to provide services to older adults through locally generated tax revenues and provide insight into their caregiver support efforts.	LTC population: Older adults in the U.S.<br><br>Study population: Representatives from 228 organizations across 15 states, delivering LTC services.<br><br>Data source(s): Phase 1: 2019 Advancing States national survey of state units on aging; telephone calls (telephone calls to state units on aging, area agencies on aging, aging services providers, county auditor offices, and Secretary of State offices); online reviews and web searches. Phase 2: Qualtrics survey to local funding organizations	High
Lei et al. 2022(34)	China	LTCI pilot	Quasi-experimental design	To evaluate the impact of LTCI on the covered populations in the LTCI pilots across different cities from 2015 to 2017, and to study	Older adults (aged 65+) from 152 cities: n=3,423<br>Treatment group (coverage): n=187<br>Control group (no coverage): n=3,236.	High

Author Year	Country	Initiative	Design	Aim	Population and Data Sources	Quality Appraisal
				various outcomes (including older adults' ADL-related need for care, family burden, medical expenditures, and some important health outcomes) among covered older adults and their families.	Data source(s): Chinese Longitudinal Healthy Longevity Survey (CLHLS)	
Liu & Hu 2022(46)	China	LTCI pilot	Quasi-experimental design	To investigate the impact of the policy pilot on the income fairness of the group system and to comprehensively analyze the institutional effect of the LTCI policy pilot.	People aged 60 and over living in one of the 15 LTCI pilot areas (n=25,063) included in CHARLS.<br><br>Data source(s): China Health and Retirement Longitudinal Study (CHARLS) database, 2013, 2015, 2018 (valid samples with a 3-year period)	High
Noda et al. 2021(55)	Japan, Korea, Thailand, China, Indonesia, the Philippines	Healthcare service delivery reform policies for the ageing	Policy comparison	To compare healthcare service delivery reform policies for older adults in Japan, Korea, Thailand, China, Indonesia and the Philippines.	LTC population: Older adults in Japan, Korea, Thailand, China, Indonesia, and the Philippines.<br><br>Study population: Health officials of provincial health and welfare departments, and service providers such as doctors and LTC providers (no n provided).<br><br>Data source(s): Document reviews; interviews with key informants	Medium
Peng et al. 2022(44)	China	LTCI pilot	Policy analysis	To assess the sustainability of the LTCI system in China using a policy population economics (PPE) system.	Population of 14 pilot cities in China. Due to a lack of data, one pilot city, Shihezi, was excluded.<br><br>Data source(s): Policy documents; China Statistical Yearbook 2020; the China City Statistical Yearbook; the Chinese National Economy and Social Development Statistics Bulletin	N/A

Author Year	Country	Initiative	Design	Aim	Population and Data Sources	Quality Appraisal
Sum et al. 2022(50)	Singapore	Integrated Patient-Centred Medical Home (PCMH) model	Quasi-experimental design	To address gaps in the literature on the evaluation of costs associated with the implementation of an integrated PCMH model for community-dwelling older adults with complex needs.	Patients with high biopsychosocial health risk, aged ≥40, who resided in Whampoa (n=165)<br><br>Data source(s): Client Service Receipt Inventory (CSRI) survey	High
Tang et al. 2022(45)	China	LTCI pilot	Quasi-experimental design	To evaluate the implementation effect of the Long-Term Care Insurance (LTCI) policy, and its impact on the medical expenses and the health status of the middle-aged and elder population.	Families with people middle-aged (≥45) and older in any of the 12 cities included in the CHARLS database (n=42,591)<br><br>Data source(s): China Health and Retirement Longitudinal Study (CHARLS)	Medium
Wu et al. 2020(39)	China	LTCI pilot	Retrospective cross-sectional study	To evaluate the LTCI pilot by exploring the characteristics and care needs of claimants, and performance of the assessment tool.	Claimants: n=4810; Individuals who received benefits: n=4582;<br>Aged ≥60 years: n=4357;<br>Beneficiaries without dementia: n=3791;<br>Beneficiaries with dementia: n=791;<br>Unsuccessful claimants without dementia: n=206;<br>Unsuccessful claimants with dementia: n=22<br><br>Data source(s): Claims data	Medium
Zhang & Yu 2019(37)	China	LTCI pilot	Quasi-experimental design	To explore the outcomes and evaluate the performance of the LTCI policy in the Chinese pilot cities and estimate the willingness of Chinese citizens to expand the formal implementation of LTCI policy in China.	Older adults engaged in the implementation process of the LTCI pilots in 15 pilot cities (n=1,167)<br>Elite politicians (n=5)<br><br>Data source(s): Survey questionnaire; interviews	Medium

Author Year	Country	Initiative	Design	Aim	Population and Data Sources	Quality Appraisal
Zhang et al. 2020(36)	China	LTCI pilot	Quasi-experimental design	To examine how the use of formal care impacts the use of informal care in Shanghai, which was one of China's first long-term insurance pilots in 2016.	Families in Shanghai that include an older adult (≥60) who had used formal care provided by LTCI for 1 to 3 months and a child who is primarily responsible for daily informal care.<br>Care recipients: n=407<br>Caregivers n=407<br><br>Data source(s): Interview-based surveys	Medium
Zhou & Dai 2021(38)	China	LTCI pilot	Systematic literature review of LTC policies	To review and assess the performance and effectiveness of the LTCI policy regime in China.	Older adults in one of the 15 pilot cities.<br><br>Data source(s): Policy documents	Medium

*The countries and subnational areas covered include Austria (Vienna region), Belgium (Flanders), Canada (Ontario), Croatia, Czech Republic, England, Estonia (Tallinn), Finland, France, Germany, Hungary, Iceland (Reykjavik), Ireland, Italy (South Tyrol), Japan, Korea, Latvia, Lithuania, Luxembourg, Slovak Republic, Slovenia, Spain, Sweden, the Netherlands, the United States of America (Illinois and California).
Quality appraisal (score % as per # of tool items): high quality=80-100%; medium quality=50-79%; low quality= <50%

Table 2: PROGRESS-Plus characteristics reported by study

Author	P	G/S	E	SE	SC	D	A	Author	P	G/S	E	SE	SC	D	A
Alders 2022 (52)	✓			✓		✓	✓	Koumoutzis 2021 (47)							
Beauregard 2021 (45)								Koumoutzis 2022 (46)							
Chen 2020 (33)	✓	✓	✓	✓	✓	✓	✓	Lei 2022 (32)	✓	✓		✓	✓		✓
Chen 2020 (51)	✓						✓	Liu 2022 (44)	✓	✓	✓	✓	✓	✓	✓
Chen 2022 (41)	✓	✓	✓	✓	✓	✓	✓	Noda 2021 (53)							
Chiu 2019 (50)								Peng 2022 (42)	✓					✓	✓
Dai 2022 (39)	✓						✓	Sum 2022 (48)	✓	✓	✓		✓	✓	✓
Feng 2021 (38)	✓						✓	Tang 2022 (43)		✓	✓	✓	✓	✓	✓
Fong 2022 (49)						✓	✓	Wu 2020 (37)	✓	✓				✓	✓
Han 2022 (40)	✓	✓				✓	✓	Zhang 2019 (35)	✓	✓		✓	✓	✓	✓
Hashiguchi 2020 (30)	✓			✓			✓	Zhang 2020 (34)	✓	✓	✓	✓	✓		✓
Kotschy 2022 (54)	✓					✓		Zhou 2021 (36)							

P=Place of residence; G=Gender/Sex; E=Education; SE=Socio-economic status; SC=Social capital; D=Disability; A=Age

Search Strategies

Search strategies are available via searchRxiv for the following information sources:

- MEDLINE: https://doi.org/10.1079/searchRxiv.2023.00214
- CINAHL: https://doi.org/10.1079/searchRxiv.2023.00213
- EconLit: https://doi.org/10.1079/searchRxiv.2023.00216
- Embase: https://doi.org/10.1079/searchRxiv.2023.00217
- ProQuest Dissertations and Theses: https://doi.org/10.1079/searchRxiv.2023.00220
- WHO ICTRP: https://doi.org/10.1079/searchRxiv.2023.00219
- ClinicalTrials.gov: https://doi.org/10.1079/searchRxiv.2023.00218

CADTH Grey Matters

All websites included in the Health Economics category of CADTH Grey Matters were searched or browsed for relevant records:

- Centre for Health Economics and Policy Analysis (McMaster University)
- Centre for Reviews and Dissemination (University of York)
- Economic Burden of Illness in Canada (Public Health Agency of Canada)
- Health Economics Research Unit (University of Aberdeen)
- IDEAS/RePEc database
- IHE publications (Institute of Health Economics, Canada)
- National Quality Measures Clearinghouse (Agency for Healthcare Research and Quality, U.S.)
- OHE publications (Office of Health Economics, United Kingdom)
- Ontario Case Costing Initiative (Ontario Ministry of Health and Long-Term Care)
- THETA Publications and Knowledge Translation to Policy (KT) Activities (Toronto Health Economics and Technology Assessment Collaborative)

If the website had a search feature, the search was as follows: ("long-term care" OR "long term care")

If there were too many results, it was narrowed by adding the following: AND (financing OR funding)

Results: 34 (38 minus 4 duplicates)

Data extraction form

General information

Study ID
Enter the Covidence record number here.

Example: #34

Title
Title of paper/abstract/report

Last name of lead author

Characteristics of study
Publication type
If publication type is "other" please specify the details below.
☐Journal article
☐Dissertation/thesis
☐Report
☐Other Click or tap here to enter text.

Aim/purpose/objective of study
Enter the research question or hypothesis.

Study design
Studies might not always explicitly state the design, in which case select the best option based on the methods section. If unclear, please include any relevant information in the notes section below.
☐Randomized controlled trial
☐Non-randomized experimental study
☐Quasi-experimental design

☐Cohort study
☐Cross-sectional study
☐Case–control study
☐Case series
☐Case report
☐Economic evaluation
☐Comparative case study
☐Policy analysis
☐Qualitative research (specific approach)
☐Other Click or tap here to enter text.

Country/setting
If the study is comparing countries/settings, ensure all the countries are listed, separated by semicolons. If the study does not mention the country but mentions a region, enter the region.

Population description
Describe the overall population. For more than one population start a new paragraph.

Gender
Enter the breakdown of population gender if reported.

Sexual orientation
Enter the breakdown of population sexuality if reported.

Race
Enter the breakdown of population racial composition if reported.

Age
Enter the overall age of population if reported. If age is reported as a range (e.g. 35–50 years) enter the relevant range and accompanying value with a colon in between (e.g. 60–79: 50%). If there are multiple ranges, separate using a semicolon (e.g. 60–79: 50%; 80–99: 50%).

Place of residence

Enter the breakdown of places of residence (e.g. rural, urban, suburban, long-term care [LTC] congregate setting, etc.) if reported.

Occupation
Enter the breakdown of occupations if reported.

Religious backgrounds
Enter the breakdown of religious backgrounds if reported.

Education
Enter the breakdown of educational backgrounds if reported.

Socioeconomic status
Enter the breakdown of socioeconomic status if reported.

Social capital
Social capital refers to social relationships, networks and social support. Enter information related to the social capital of the population if reported.

Disability
Enter the breakdown of the population disability categories if reported.

LTC financing
LTC system description
Describe the overall LTC system. For more than one system start a new paragraph.

Financing description
Describe how the LTC system is financed. For more than one system start a new paragraph.

Funding mechanism
Enter the details of the funding mechanism(s) in the relevant column. Enter NA in boxes where the funding mechanism is not applicable. Include the percentage mix for each funding mechanism if reported. If not reported, enter NR.

Funding mechanism	Details	Percentage mix
Guarantees		
Insurance		

Fees for service		
Loans		
Repayable contributions		
Result-based financing		
Equity		
Bonds		
Charitable donations		
Local government subsidies		
Taxes		
Co-payment by users		
Central government subsidies		
Other		

Service description
Describe the services covered under the programme policy reform or initiative if reported.

Coverage
How does the initiative/programme/policy/reform address LTC coverage? Enter NA if not addressed.

Quality
How does the initiative/programme/policy/reform address LTC quality? Enter NA if not addressed.

Financial protection
How does the initiative/programme/policy/reform address LTC financial protection? Enter NA if not addressed.

Financial sustainability
How does the initiative/program/policy/reform address LTC financial sustainability? Enter NA if not addressed.

Empirical evaluation
Intervention
What is the initiative/programme/policy/reform?

Comparator

What is the initiative/programme/policy/reform being compared to? If there is no comparator enter NA.

Intervention time frame
What is the time frame of the intervention?
Examples: policy put in place in June of 2017; pilot project from January 2017 to August 2019.

Data sources
Enter the sources of data. If there are multiple sources separate them with a semi-colon.

Dates of data collection
Enter the start and end date of data collection in yyyyy/mm format. If data sources are distinct years enter all years of data collection separated by commas.
Examples: 2017/11–2018/03; 2014; 2019; 2021.

Key findings and/or outcomes
Enter the key findings related to LTC financing.

Author's overall conclusion
Enter the authors' conclusions as it relates to LTC financing.

Reviewer notes
Enter any relevant notes or comments that are not captured elsewhere.
