# Supplementary material for: Recent innovations in long-term care coverage and financing: a rapid scoping review
Source: BMJ Open. 2024 Feb 22;14(2):e077309. doi: 10.1136/bmjopen-2023-077309 (PMC10884182; doi:10.1136/bmjopen-2023-077309)
Supplement: Author's manuscript [file bmjopen-2023-077309.draft_revisions.pdf]

BMJ Open is committed to open peer review. As part of this commitment we make the peer review history of every article we publish publicly available.

When an article is published we post the peer reviewers' comments and the authors' responses online. We also post the versions of the paper that were used during peer review. These are the versions that the peer review comments apply to.

The versions of the paper that follow are the versions that were submitted during the peer review process. They are not the versions of record or the final published versions. They should not be cited or distributed as the published version of this manuscript.

BMJ Open is an open access journal and the full, final, typeset and author-corrected version of record of the manuscript is available on our site with no access controls, subscription charges or pay-per-view fees (http://bmjopen.bmj.com).

If you have any questions on BMJ Open's open peer review process please email info.bmjopen@bmj.com

BMJ Open

Recent Innovations in Long-Term Care Coverage and Financing: A Rapid Scoping Review

Journal:	*BMJ Open*
Manuscript ID	bmjopen-2023-077309
Article Type:	Original research
Date Submitted by the Author:	04-Jul-2023

Complete List of Authors:	Macdonald, Marilyn; Dalhousie University, Aligning Health Needs and Evidence for Transformative Change (AH-NET-C): A JBI Centre of Excellence; Dalhousie University, School of Nursing<br>Weeks, Lori; Dalhousie University, Aligning Health Needs and Evidence for Transformative Change (AH-NET-C): A JBI Centre of Excellence; Dalhousie University, School of Nursing<br>Langman, Erin; Dalhousie University, Aligning Health Needs and Evidence for Transformative Change (AH-NET-C): A JBI Centre of Excellence; Dalhousie University, School of Nursing<br>Caruso, Julie; Dalhousie University, Aligning Health Needs and Evidence for Transformative Change (AH-NET-C): A JBI Centre of Excellence; Dalhousie University, School of Nursing<br>Tricco, Andrea; Unity Health Toronto, St. Michael's Hospital, Li Ka Shing Knowledge Institute; University of Toronto, Dalla Lana School of Public Health, Epidemiology Division and Institute of Health Policy, Management, and Evaluation<br>Pham, Ba; University of Toronto, (retired)<br>Straus, Sharon; Unity Health Toronto, St. Michael's Hospital, Li Ka Shing Knowledge Institute; University of Toronto, Institute of Health Policy, Management and Evaluation<br>Mishra, Sujata; University of Toronto, Institute of Health Policy, Management and Evaluation; University of Toronto Dalla Lana School of Public Health<br>Isaranuwatchai, Wanrudee; Health Intervention and Technology Assessment Program, (HITAP); Unity Health Toronto<br>Cormack, Gordon; University of Waterloo, David R. Cheriton School of Computer Science<br>Grossman, Maura; University of Waterloo, David R. Cheriton School of Computer Science; York University, Osgoode Hall Law School<br>Yakubovich, Alexa R; Dalhousie University, Department of Community Health and Epidemiology; St Michael's Hospital, Li Ka Shing Knowledge Institute<br>Mojbafan, Arezoo; Dalhousie University, Aligning Health Needs and Evidence for Transformative Change (AH-NET-C): A JBI Centre of Excellence; Dalhousie University, School of Nursing<br>Ignaczak, Melissa; Dalhousie University, Aligning Health Needs and Evidence for Transformative Change (AH-NET-C): A JBI Centre of Excellence; Dalhousie University, School of Nursing<br>Leid, Caron; SPOR Evidence Alliance, Patient and public partner<br>Watt, Jennifer; University of Toronto<br>Stevens, Susan; Nova Scotia Health Authority

Khan, Tayaba; SPOR Evidence Alliance, Patient and public partner
Curran, Janet; Dalhousie University, Aligning Health Needs and Evidence for Transformative Change (AH-NET-C): A JBI Centre of Excellence; Dalhousie University, School of Nursing
Moody, Elaine; Dalhousie University, Aligning Health Needs and Evidence for Transformative Change (AH-NET-C): A JBI Centre of Excellence; Dalhousie University, School of Nursing
Rodrigues, Ricardo ; Universidade de Lisboa, SOCIUS/CSG, ISEG (Lisbon School of Economics & Management)
Roach, Sheri; Dalhousie University, Aligning Health Needs and Evidence for Transformative Change (AH-NET-C): A JBI Centre of Excellence; Dalhousie University, School of Nursing
MacNeil, Morgan; Dalhousie University, . Aligning Health Needs and Evidence for Transformative Change (AH-NET-C): A JBI Centre of Excellence; Dalhousie University, School of Nursing

Keywords: Aging, Health Services Accessibility, Health Services for the Aged

SCHOLARONE™
Manuscripts

I, the Submitting Author has the right to grant and does grant on behalf of all authors of the Work (as defined in the below author licence), an exclusive licence and/or a non-exclusive licence for contributions from authors who are: i) UK Crown employees; ii) where BMJ has agreed a CC-BY licence shall apply, and/or iii) in accordance with the terms applicable for US Federal Government officers or employees acting as part of their official duties; on a worldwide, perpetual, irrevocable, royalty-free basis to BMJ Publishing Group Ltd ("BMJ") its licensees and where the relevant Journal is co-owned by BMJ to the co-owners of the Journal, to publish the Work in this journal and any other BMJ products and to exploit all rights, as set out in our licence.

The Submitting Author accepts and understands that any supply made under these terms is made by BMJ to the Submitting Author unless you are acting as an employee on behalf of your employer or a postgraduate student of an affiliated institution which is paying any applicable article publishing charge ("APC") for Open Access articles. Where the Submitting Author wishes to make the Work available on an Open Access basis (and intends to pay the relevant APC), the terms of reuse of such Open Access shall be governed by a Creative Commons licence – details of these licences and which Creative Commons licence will apply to this Work are set out in our licence referred to above.

Other than as permitted in any relevant BMJ Author's Self Archiving Policies, I confirm this Work has not been accepted for publication elsewhere, is not being considered for publication elsewhere and does not duplicate material already published. I confirm all authors consent to publication of this Work and authorise the granting of this licence.

For peer review only

TITLE: Recent Innovations in Long-Term Care Coverage and Financing: A Rapid Scoping Review

AUTHORS: Marilyn Macdonald[1,2] (marilyn.macdonald@dal.ca), Lori E. Weeks[1,2] (lori.weeks@dal.ca), Erin Langman[1,2] (erin.langman@dal.ca), Julie Caruso[1,2] (julie.caruso@dal.ca), Andrea C. Tricco[3,4,5] (andrea.tricco@unityhealth.to), Ba' Pham[6] (ba.pham@theta.utoronto.ca), Sharon Straus[3,7] (sharon.straus@utoronto.ca), Sujata Mishra[6] (sujata.mishra@mail.utoronto.ca), Wanrudee Isaranuwatchai[7,8,9] (wanrudee.i@hitap.net), Gordon V. Cormack[10] (gvcormack@uwaterloo.ca), Maura R. Grossman[10,11] (maura.grossman@uwaterloo.ca), Alexa R. Yakubovich[3,12] (alexa.yakubovich@dal.ca), Arezoo Mojbafan[1,2] (arezoo.mojbafan@dal.ca), Melissa Ignaczak[1,2] (melissa.ignaczak@dal.ca), Caron Leid[13] (caronleid@gmail.com), Jennifer Watt[6] (jennifer.watt@utoronto.ca), Susan Stevens[14] (susan.stevens@nshealth.ca), Tayaba Khan[13] (drtayabakhan@gmail.com), Janet Curran[1,2] (jacurran@dal.ca), Elaine Moody[1,2] (elaine.moody@dal.ca), Ricardo Rodrigues[15] (ricardo.rodrigues@iseg.ulisboa.pt), Sheri Roach[1,2] (sheri.roach@dal.ca), Morgan X. MacNeil[1,2] (morgan.macneil@dal.ca)

CORRESPONDING AUTHOR: Marilyn Macdonald, Room 130, Forrest Bldg., Dalhousie University, PO Box 15000, 5869 University Avenue, Halifax NS B3H 4R2, 902-494-2433

1. Aligning Health Needs and Evidence for Transformative Change (AH-NET-C): A JBI Centre of Excellence, Halifax, Nova Scotia, Canada; 2. School of Nursing, Dalhousie University, Halifax, Nova Scotia, Canada; 3. Li Ka Shing Knowledge Institute, St. Michael's Hospital, Unity Health Toronto, Toronto, Canada; 4. Epidemiology Division and Institute of Health Policy, Management, and Evaluation, Dalla Lana School of Public Health, University of Toronto, Toronto, Ontario, Canada; 5. Queen's Collaboration for Health Care Quality: A JBI Centre of Excellence, Queen's University School of Nursing, Kingston, Ontario, Canada; 6. University of Toronto, Toronto, Ontario, Canada; 7. Institute of Health Policy, Management and Evaluation, University of Toronto, Toronto, Canada; 8. Unity Health Toronto, Toronto, Ontario, Canada; 9. Health Intervention and Technology Assessment Program (HITAP), Thailand; 10. David R. Cheriton School of Computer Science, University of Waterloo, Waterloo, Ontario, Canada; 11. Osgoode Hall Law School, York University, Toronto, Ontario, Canada; 12. Department of Community Health and Epidemiology, Dalhousie University; 13. Patient and public partner; 14. Nova Scotia Health, Nova Scotia, Canada; 15. SOCIUS/CSG, ISEG (Lisbon School of Economics & Management), Universidade de Lisboa, Lisboa, Portugal

For peer review only

KEYWORDS: Long-Term Care; Aging; Health Care Costs; Sustainable Development

WORD COUNT: 4,700

ABSTRACT

Objectives: The objective of this scoping review was to identify, chart and analyze the literature on recent initiatives to improve long-term care (LTC) coverage, financial protection and financial sustainability.

Methods: Four databases and four sources of grey literature were searched for reports published between 2017 and 2022. After using a supervised machine learning tool to rank titles and abstracts from most to least likely to be of interest, two reviewers independently screened sources against inclusion criteria. Data were extracted using a form designed to answer the review questions, and analyzed using descriptive qualitative content analysis, where data were categorized according to a pre-specified framework designed to capture the outcomes of interest.

Results: Of 24 reports, 22 were published in peer-reviewed journals, and two were grey literature sources. Study designs included quasi-experimental, policy analysis or comparison, qualitative research, comparative case study, cross-sectional study, systematic literature review, economic evaluation, and survey. The reports covered initiatives in China, the United States, Singapore, Taiwan, and the Netherlands. Included studies addressed coverage, which varied based on level of disability, income, rural/urban residence, employment, and citizenship. Studies also addressed financial protection, including out-of-pocket (OOP) expenditures, co-payments, and risk of poverty related to costs of care. Lastly, the reports addressed challenges to financial sustainability such as lack of service coordination and system integration, insufficient economic development, and inadequate funding models.

For peer review only

Conclusions: Initiatives where LTC insurance is mandatory and accompanied by a commensurate funding scheme are situated to facilitate ageing in place. Efforts to expand population coverage are common across the initiatives, with the potential for wider economic benefits. In addition, initiatives that enable older people to access the services needed while avoiding OOP-induced poverty contribute to improved health and well-being. Preserving health in older people longer may alleviate downstream costs and contribute to financial sustainability.

ARTICLE SUMMARY

Strengths and Limitations of this Study

- This study uses the WHO definition of long-term care, which includes care across the continuum for older adults and does not exclude studies based on the type of care provided.
- This study identifies gaps in the existing literature and suggests areas for improvement, such as the importance of capturing equity measures.
- Due to the design of the review, long-established LTC programs and new initiatives that are yet to be evaluated may not be captured, as well as proprietary reports and government and health authority internal reports.
- Some of the included reports analyzed textual data from policy data in the absence of implementation data.

INTRODUCTION

The proportion of the global population aged 65 years and above is projected to grow from 10% in 2022 to 16% in 2050. (1) Due to rapidly aging populations, a greater prevalence of chronic conditions including dementia, gains in life expectancy, changing family dynamics and living arrangements, and shortages of paid and unpaid caregivers, the demand for long-term care (LTC) services continues to expand. (2) Governments worldwide are challenged to create and

For peer review only

adapt public programs to ensure that older adults have access to high quality LTC services. Accordingly, many governments undertook LTC system reforms.

In most countries, LTC services comprise a fraction of all healthcare expenditures. (3) The bulk of costs is covered by governments and social insurance with most people cared for in their own homes. (4) In general, systems of public LTC coverage can be clustered into two main types: 1) universal publicly funded system, and 2) a means-tested, safety-net system where public funding is available for those who cannot afford to pay for LTC. Universal LTC is available in many countries, including Japan, Germany, Luxembourg, the Netherlands, South Korea, Denmark, Finland, Norway, and Sweden. (5) Other countries, such as England and the United States, rely heavily on a means-tested, safety-net system. (5) The two main types of public LTC coverage can also be used in combination, as in the case of Canada, Austria, Australia, France, Greece, Ireland, Italy, Spain, Poland, New Zealand, and Switzerland. (5) A systematic review (6) noted that each country features its own distinct public LTC insurance (LTCI) implementation techniques and that public LTCI in all countries must continuously adapt to local conditions to function sustainably.

Researchers have noted the substantial variations in LTC funding approaches between low- and high-income regions, as well as the difficulty in finding relevant indicators and measures that can be adapted to different settings. Through a scoping review, Seyede and colleagues identified several indicators (e.g., OOP expenditures, geographic coverage, and proportion of older people receiving LTC) to measure progress towards Universal Health Coverage in the context of population aging but they noted that many of these indicators would not be feasible in low- and middle-income countries. (7)

For peer review only

For peer review only - http://bmjopen.bmj.com/site/about/guidelines.xhtml

For the purposes of this review LTC includes service provision in the community, such as homecare and support, rehabilitative care, palliative care, residential care homes, assisted living facilities, nursing homes, as well as any other institutional setting. (8) LTC coverage not only includes care provided in facilities, it also includes programs such as cash benefits, community services in kind, day programs, residential facility care and home care. (8) According to WHO, "financial protection is achieved when direct payments made to obtain health services do not expose people to financial hardship and do not threaten living standards." (9 para.1) Financial protection via Universal Health Coverage is also a WHO Sustainable Development goal. (10) Financial sustainability "refers to the extent to which a given set of fiscal policies for LTC does not shift too large a financial burden on future generations (i.e., intergenerational fiscal equity) and ensures that "ends meet." (11)

The focus of this scoping review is LTC funding, specifically, the knowledge gap regarding what is known about recent LTC initiatives that directly address financing. Addressing the complex needs of the world's rapidly aging population will require a focus on improving aspects of LTC service coverage, financial protection, and financial sustainability. The primary objective of this scoping review is to identify, chart and analyze the literature on recent public initiatives (i.e., reforms, policies and programs) that have been undertaken to improve LTC coverage, financial protection and financial sustainability.

Review Question

What recent initiatives have been implemented to improve service coverage, financial protection and financial sustainability in the LTC sector for persons 60 years and older?

METHODS

For peer review only

This rapid scoping review was conducted in accordance with the JBI methodology for scoping reviews (12) and adheres to the reporting standards outlined in the Preferred Reporting Items for Systematic Reviews and Meta-Analyses extension for scoping reviews (PRISMA-ScR). (13) The WHO guide on rapid reviews (14) was consulted to help tailor the rapid review methods to decision-makers' needs. The Strategy for Patient Oriented Research – Evidence Alliance (SPOR-EA) processes for integrated knowledge translation were followed. (15-18) The WHO Centre for Health Development (WHO Kobe Centre) contracted this review. An agreement of work plan was provided to the contractor in lieu of a published protocol.

Search Strategy

After a limited search for seed articles in MEDLINE, two librarians (EL, JC) created an initial search strategy for MEDLINE that was peer-reviewed by a third librarian using Peer Review of Electronic Search Strategies (PRESS). (19) A librarian translated the search strategy for CINAHL, EMBASE and EconLit. Sources of grey literature included ClinicalTrials.gov, WHO International Clinical Trial Registry Platform (ICTRP) and ProQuest Dissertations and Theses. Canada's Drug and Health Technology Agency (CADTH) Grey Matters database was also used to identify additional sources of grey literature under the category of "health economics." All search strategies are available in the online supplemental file and are reported using Preferred Reporting Items for Systematic reviews and Meta-Analyses literature search extension (PRISMA-S). (20)

Inclusion Criteria

Studies published in any language, from January 2017 to August 25, 2022, that captured recent LTC initiatives for people aged 60 and older, involved evaluation and directly addressed financing were included. Eligible study designs included randomized control trials (RCT), non-

For peer review only

randomized controlled studies, before and after studies, interrupted time-series, quasi-experimental studies, qualitative studies, mixed methods, economic evaluation, systematic reviews and policy analysis reports.

Study Screening and Data Extraction

Bibliographic records were identified for screening using the Continuous Active Learning® (CAL®) tool (21, 22) which uses supervised machine learning to rank titles and abstracts from most to least likely to be of interest. Results of the database searches were uploaded to Covidence (Veritas Health Innovation, Melbourne, Australia) and duplicates were removed. Three reviewers independently reviewed the first 1500 titles and abstracts. Potentially relevant full-text articles were then screened by two team members. All discrepancies were resolved by discussion or a third reviewer. The results of the search and the screening process are reported in full and presented in a Preferred Reporting Items for Systematic Reviews and Meta-Analyses (PRISMA) 2020 flow diagram. (23)

A data-extraction form was developed, and pilot-tested using a random sample of five included reports. Data were extracted on study characteristics, outcomes, and PROGRESS-Plus (24, 25) characteristics, where possible (see the online supplemental file). Reports written in languages other than English were reviewed and data extracted by team members fluent in those languages.

Quality Appraisal

Although quality appraisal is typically not conducted for a scoping review (14), it was conducted at the request of the commissioner. The appraisal was conducted by one team member and independently verified by a second team member. JBI methodological quality assessment tools (26-28) were selected based on the designs of included reports. Review articles were

For peer review only

appraised using the Health Evidence™ Quality Appraisal Tool. (29) Discrepancies were resolved through team discussion.

Analysis

Using descriptive qualitative content analysis, (12) data was categorized according to the key outcomes of interest, coverage, financial protection and financial sustainability in LTC. Supplemental Table 1 outlines study characteristics, including initiatives and quality appraisal of included reports.

Patient and Public Involvement

Two public partners, co-authors, attended regular team meetings over a six-month period and provided input into the design of the study. They also reviewed and commented on report drafts, tools, and manuscript drafts, as well as dissemination activities.

RESULTS

The search retrieved 51,766 electronic database and grey literature records. Following screening at the title/abstract level, 100 reports were identified for full-text review (Figure 1). Of these, 24 reports met the inclusion criteria for this rapid scoping review.

Characteristics of Included Studies

Of the 24 reports, 22 were published in peer-reviewed journals, and two were grey literature sources. (30, 31) Just over half of the reports addressed initiatives in China (n=13). (32-44) The remainder originated in the United States (U.S., n=3) (45-47), Singapore (n=2) (48, 49), Taiwan (n=2) (50, 51), and the Netherlands (n=1). (52) Three reports covered multiple countries. (30, 31, 53) All 24 reports were published between 2019 and 2022. The study designs included quasi-experimental (n=7) (32, 34, 35, 41, 43, 44, 48), policy analysis or comparison (n=6) (31, 38, 42, 49, 51, 53), qualitative research (n=3) (40, 47, 50), comparative case study (n=2) (30,

For peer review only

45), cross-sectional study (n=2) (33, 37), systematic literature review (n=2) (36, 39), economic evaluation (n=1) (52), and survey (n=1) (46) (see Supplemental Table 1).

Included studies analyzed secondary data (n=10) (30-33, 37, 41, 43, 44, 48, 52), policy documents (n=6) (36, 38, 39, 42, 51, 53), interviews (n=6) (33, 40, 45, 49, 50, 53) and survey data (n=3). (35, 46, 47) One study had data from both policy documents and interviews. (53)

All included sources focused on people aged 60 and above, but not all included data collected from older adults. Six reports included data from LTC stakeholders (40, 45-47, 50, 53). For example, Beauregard and Miller (45) conducted interviews with bureaucrats, consumer and provider advocacy groups, consultants and policy experts. For these six studies, we have indicated both who the data concerned and who provided the data (see Supplemental Table 1). Of the remaining 18 reports, nine (32-35, 37, 41, 43, 44, 48) reported findings from a LTC population and nine (30, 31, 36, 38, 39, 42, 49, 51, 52) focused on analyzing policy, comparing LTC systems across countries, or analyzing government spending on LTC (see Supplemental Table 1).

Quality Appraisal

Twenty-one out of 24 reports underwent critical appraisal. The three reports excluded from appraisal included two (30, 31) summarizing and interpreting existing studies and the third (42) used a design that approximated a quality appraisal. Overall studies were appraised at moderate to high in quality (see Supplemental Table 1).

Long-Term Care Initiatives

The LTC initiatives are described in Table 1.

Table 1: LTC Initiatives

Initiative	Country	Author Year	Description

For peer review only

LTCI Pilots	China	Chen et al. 2020 (33)<br>Chen & Ning 2022 (41)<br>Dai et al. 2022 (39)<br>Feng 2021 (38)<br>Han & Shen 2022 (40)<br>Lei et al. 2022 (32)<br>Liu & Hu 2022 (44)<br>Peng et al. 2022 (42)<br>Tang et al. 2022 (43)<br>Wu et al. 2020 (37)<br>Zhang & Yu 2019 (35)<br>Zhang et al. 2020 (34)<br>Zhou & Dai 2021 (36)	• Nearly universal health insurance coverage yet did not include community LTC coverage before the pilots<br>• LTCI pilots instituted to learn and plan how best to deliver LTC<br>• Began in 2016 with 16 cities and expanded to 49 cities by 2020<br>• Pilot cities explored multiple sources of financing and customized benefits packages based on their unique needs<br>• Aimed to promote formal (paid) care, while also encouraging informal care
CareShield Life	Singapore	Fong & Borowski 2022 (49)	• Legislated LTC social insurance<br>• Coverage for citizens and permanent residents born on or after January 1, 1980; entry age is 30; inclusive of people with severe disabilities and the economically disadvantaged<br>• Assessed for eligibility by the Ministry of Health (i.e., disability in performing at least three ADLs)<br>• Lifetime coverage; a claim can be made at any age<br>• Benefits increase annually by 2% for the first five years; premiums stop at age 67; the later a claim is made the higher the monthly payout
LTC 2.0 Reform	Taiwan	Chen & Fu 2020 (51)<br>Chiu et al. 2019 (50)	• Expansion of LTC to target aging in place<br>• Eligibility determined by assessing ADL, IADL, cognition, behavioural changes, rehabilitation, home situation, and caregiver stress<br>• Eligible populations: people ≥65 with ADL limitations as well as frailty; 2. people with dementia aged ≥50<br>• Changes included a reduction of co-payments, a simplified contract system for service providers, modified care worker service fees, government subsidies for rural areas, family caregiver support centres, dementia support centres, and community care stations
Community First Choice	U.S.	Beauregard & Miller 2021 (45)	• The federal government program allowing states to expand Medicaid home and community-based services through the Affordable Care Act<br>• 6% federal increase in matching payments to shift spending from institutional to home-based care<br>• Provides individuals considered to be at an institutional level of care with ADL and IADL assistance<br>• Agency-delivered or self-directed (clients take responsibility for hiring, supervising, and de-hiring workers)
LTC Reform	The Netherlands	Alders & Shut 2022 (52)<br>Hashiguchi & Llena-Nozal 2020 (30)<br>Kotschy & Bloom, 2022 (31)	• Reform initiated in response to high institutional care costs and focused on intensive home care as a substitute for institutional care<br>• Social care responsibility transferred to municipalities

LTCI=long-term care insurance; ADL=activities of daily living; IADL=instrumental activities of daily living

For peer review only

Equity

Although the term "gender" was used consistently, only biological sex (female-male) was reported. Nine studies reported gender (32-34, 37, 40, 41, 43, 44, 48) (42.3% male). Place of residence was occasionally unclear. Education level and socio-economic status, and age were reported in multiple ways based on the study design and variables included. Table 2 reports the PROGRESS-Plus characteristics reported by study.

Table 2: PROGRESS-Plus characteristics reported by study

Author	P	G/S	E	SE	SC	D	A	Author	P	G/S	E	SE	SC	D	A
Alders 2022 (52)	✓			✓		✓	✓	Koumoutzis 2021 (47)							
Beauregard 2021 (45)								Koumoutzis 2022 (46)							
Chen 2020 (33)	✓	✓	✓	✓	✓	✓	✓	Lei 2022 (32)	✓	✓		✓	✓		✓
Chen 2020 (51)	✓						✓	Liu 2022 (44)	✓	✓	✓	✓	✓	✓	✓
Chen 2022 (41)	✓	✓	✓	✓	✓	✓	✓	Noda 2021 (53)							
Chiu 2019 (50)								Peng 2022 (42)	✓					✓	✓
Dai 2022 (39)	✓						✓	Sum 2022 (48)	✓	✓	✓		✓	✓	✓
Feng 2021 (38)	✓						✓	Tang 2022 (43)		✓	✓	✓	✓	✓	✓
Fong 2022 (49)						✓	✓	Wu 2020 (37)	✓	✓				✓	✓
Han 2022 (40)	✓	✓				✓	✓	Zhang 2019 (35)	✓	✓		✓	✓	✓	✓
Hashiguchi 2020 (30)	✓			✓			✓	Zhang 2020 (34)	✓	✓	✓	✓	✓		✓
Kotschy 2022 (54)	✓					✓		Zhou 2021 (36)							

P=Place of residence; G=Gender/Sex; E=Education; SE=Socio-economic status; SC=Social capital; D=Disability; A=Age

LTC Insurance Coverage

The LTCI pilots in China represented LTC in the home setting and in institutions. The countries involved in the remaining initiatives have been delivering LTC in home care and have moved to either enhance initiatives or to reform existing initiatives. Table 3 outlines the five initiatives, associated studies and a summary of coverage by initiative.

Table 3: Coverage by Country and Initiative

Initiative	Country	Author Year	Coverage	Coverage Effect

For peer review only

LTCI pilots	China	Dai et al. 2022 (39) Feng et al. 2021 (38) Han & Shen 2022 (40) Lei et al. 2022 (32) Peng et al. 2022 (42) Zhou & Dai 2021 (36)	Eligible populations: seven pilot cities provided coverage to UEBM participants only. Eight cities expanded coverage to include: both urban and non-employees of UEBMI and URBMI (n=2); urban employees enrolled in UEBMI and rural and urban residents aged 60 and over enrolled in URBMI and NRCMS (n=1); all rural and urban participants in UEBM, UEBMI, and URBMI (n=5)	Eligibility criteria is determined by assessment of disability (assessment tools varied). People with severe disability were eligible in all pilot cities (32, 40), with some cities including coverage for moderate/mild disability (n=3) (32) and dementia (n=6) (40)
CareShield Life	Singapore	Fong & Borowski 2022 (49)	Eligible population: citizens and permanent residents born on or after 1 January 1980 who are ≥ age 30.	The overall assessment indicates benefits are modest and that depth of coverage is sacrificed to breadth.
LTC 2.0	Taiwan	Chen & Fu 2020 (51)	Eligible populations: people ≥65 with ADL limitations as well as frailty, and people with dementia aged ≥50.	Eligibility criteria determined by assessing ADL, IADL, cognition, behavioural changes, rehabilitation, home situation, and caregiver stress.<br>LTC includes health prevention and supportive community services; excludes institutional care.
Community First Choice	U.S.	Beauregard & Miller 2021 (45)	Eligible populations: Individuals who are Medicaid-eligible. States who adopt the program must provide services state-wide to everyone eligible.	Beauregard and Miller (45) found that states were concerned that this level of coverage would cause budgetary issues, and that "the added cost of CFC was not something that state officials were able or willing to shoulder."p.184
LTCI Reform	The Netherlands	Alders & Shut 2022 (52)	Eligible populations: Age 60 years and above. Social care transferred to municipalities, institutional care a federal responsibility, strict rules for eligibility.	Municipalities with low economic solvency directed those eligible to institutional care

UEBMI=Urban Employee Basic Medical Insurance; URBMI=Urban Resident Basic Medical Insurance; NRCMS=New Rural Cooperative Medical Scheme; CFC=Community First Choice; ADLs = activities of daily living; IADLs = instrumental activities of daily living

Initiative – LTC Pilots China

Six reports (32, 36, 38-40, 42) addressed coverage (Table 3) where near-universal health insurance coverage is provided through three social health schemes: the Urban Employee Basic Medical Insurance (UEBMI), the Urban Resident Basic Medical Insurance (URBMI) and the New Rural Cooperative Medical Scheme (NRCMS) for rural residents. The pilots across 15

For peer review only

cities in China were funded through these existing schemes and largely covered the severely disabled. (32, 36, 40) Unmet care needs related to ADL and IADL were common in the absence of LTCI. (32) Seven of the pilot cities provided coverage for urban residents only, while eight expanded to varying degrees to provide coverage to rural areas as well as the non-employed. (39) Eligible participants are covered for both institutional and home care, however, in some cities, coverage for institutional care was more comprehensive than for home care. (39, 40)

Initiative – CareShield Life Singapore

In 2019, Singapore implemented a mandatory social insurance scheme for citizens and permanent residents aged 30 and older born on or after January 1, 1980. Eligibility is determined by disability assessment (disability in performing at least three ADLs). CareShield Life (49) provides lifetime coverage, a claim can be made at any age, and premiums stop at age 67.

Initiative – Taiwan LTC 2.0 Plan

Taiwan's publicly funded LTC 2.0 Plan (51) is directed to aging in place, and institutional care is not included in the initiative. The program's successes included expanding coverage through the reduction of co-payments from 30% to 16% and waiving these for low-income users. The plan also includes family caregiver support centres as well as dementia support centres for rapid diagnosis and access to services. Community care stations offering healthy living programs focused on exercise and health self-management are part of the program. (51)

Community First Choice – U.S.

Medicaid-supported LTC services and supports excludes many older people with a disability, and that only 11% of older adults are Medicaid eligible (qualifiers include citizenship or permanent residency, income, household size, disability, family status and other factors). (47)

For peer review only

Therefore, communities continuously fundraise to provide services to older people who do not qualify for Medicaid and cannot afford to purchase LTC services. (47) One recent initiative is the CFC Program (45) through which the federal government provides participating states with matching funding targeted at providing the services needed in the home setting to offset the need for institutional care. Many states are ideologically opposed to added government oversight (45) and/or may be fiscally unable to meet matching funding to maintain the program.

Initiative – Netherlands LTC Reform

The Netherlands has a long history of comprehensive public coverage of LTC (52), and a recent reform to address increasing costs involved shifting the financing of home care away from the central government to municipalities, while maintaining the financing of institutional care. Municipalities where the fiscal status is sound are well situated to manage the shift, while those that are not find themselves in the position of directing people with home care needs to institutional care which is still financed by the central government. This was an unintended effect of the reform.

Financial protection in LTC

Eleven reports (30-33, 40, 41, 43, 48, 49, 51, 52) addressed financial protection, two of which focused on OOP expenses. One (38) additional report contributed points relevant to financial protection. Table 4 outlines financial protection in relation to the five initiatives.

Table 4: Financial Protection Outcomes by Country and Initiative

Initiative	Country	Author Year	Financial protection outcomes
LTCI pilots	China	Chen & Ning 2022 (41)<br>Feng et al. 2021 (38)<br>Han & Shen 2022 (40)<br>Lei et al. 2022 (32)	• LTCI significantly reduced inpatient OOP expenditure, but not outpatient OOP expenditure. Individuals reporting fair health had higher OOP compared to those with poor health. (41)<br>• Existing medical schemes are being used to finance the pilots; shifting of funds to LTCI could drive up OOP costs for URBMI and NRCMS enrolees. (38)<br>• LTCI pilots do not financially protect rural residents, migrant workers, and employees in new forms of

For peer review only

		Tang et al. 2022 (43)	employment. Across pilots funding criteria vary with geography, occupation, and age. LTC institutions offer greater financial protection than home care.<br>• For each additional year of LTCI coverage, OOP medical expenses were reduced by 23.5%. (40)<br>• Implementation of LTCI decreased outpatient expenses (22.82%) and inpatient expenses (19.8%) in pilot cites compared to non-pilot cities. (43)
CareShield Life	Singapore	Fong & Borowski 2022 (49)	Includes lifetime protection against catastrophic spending associated with severe disability. Although the scheme is described as providing good basic LTC care needs protection there is an expectation that over time the depth of the protection will develop based on need.
LTC 2.0	Taiwan	Chen & Fu, 2020 (51)	Changes to the LTC system included a reduction of co-payments, reducing out-of-pocket spending for older adults.
Community First Choice	U.S.	Beauregard & Miller 2021 (45)<br>Hashiguchi & Llena-Nozal 2020 (30)	In California, the costs of home care for older people with median income, no net wealth, and low care needs is not covered. It is also estimated that the public social protection system would not reduce the risk of income poverty for those earning a medium income with moderate care needs. (30)
LTCI reform	The Netherlands	Alders & Shut 2022 (52)<br>Hashiguchi & Llena-Nozal 2020 (30)<br>Kotschy & Bloom 2022 (31)	The use of asset-testing means that those without net wealth would have lower out-of-pocket costs than the median income for older people. Those with net wealth would still be able to afford out-of-pocket costs from their incomes alone. Public support only covers help with ADLs – for help with IADLs or social activities out of pocket costs can be up to 100%, depending on income and net wealth. (30)

LTCI = long term care insurance; OOP = out of pocket; URBMI = Urban Resident Basic Medical Insurance; NRCMS = New Rural Cooperative Medical Scheme; ADLs = activities of daily living; IADLs = instrumental activities of daily living

Initiative – LTCI Pilots China

Five reports directly (32, 33, 40, 41, 43) and one report indirectly (38) addressed financial protection in relation to the LTCI Pilots in China. These reports addressed (OOP) health expenditures (32, 41), the relationship between LTCI and the presence of LTC services (33); population groups without protection (40); and LTCI impact on medical services utilization (43). OOP health expenditures were statistically significantly reduced (23.5%) for inpatients but not for outpatients because OOP rates favor the institutional setting. (43) Existing medical schemes are being used to finance the pilots; shifting of funds to LTCI could drive up OOP costs for enrolees. (38)

For peer review only - http://bmjopen.bmj.com/site/about/guidelines.xhtml

Filial responsibility in China traditionally meant that family members fulfilled caregiving roles across the lifespan at rates bordering on 80%. (33) The initiation of LTCI is tied directly to the availability of LTC services for ADLs and IADLs in both rural and urban settings. (33) There was variability in who was considered eligible for LTCI. Groups such as migrant workers, individuals working in new forms of employment and rural residents were often excluded. (40) Over time, there has been a gradual expansion to include these groups.

Cities piloting LTCI have experienced a decrease in medical services utilization due to a decrease in accessing both inpatient and outpatient services. Outpatient services use decreased by 22.8%, and inpatient services by 19.8%. (43) At the same time, non-pilot cities saw these percentages rise. These reductions in medical system utilization in the presence of LTCI hold promise for more optimal usage of the medical system, support for LTCI, and thus a strategic shifting of the costs from medical to social care.

Initiative – CareShield Life Singapore's LTCI

This initiative is an example of a universally publicly funded LTC scheme that extends to an entire population. (49) The scheme includes protection against catastrophic spending associated with severe disability and financial difficulty and nominal cash benefits with regular increases aimed at fostering consumer choice and flexibility, which can complement or substitute in-kind benefits and services. Although the scheme is described as providing good basic LTC care needs protection, there is an expectation that over time the depth of the protection will develop based on need.

Initiative – LTC 2.0 Taiwan

The reform of LTC undertaken in Taiwan (51) was targeted toward assisting the population of older people to age in place. The reform focused on the expansion of services that

For peer review only

included but were not limited to dementia care, linking services such as adult daycare and respite services, family caregiver support centres, community health preventive care, links to discharge plans from hospitals and to home-based medical care. The reduction in insurance co-payments, availability of LTC support services and the subsidies provided to rural areas demonstrate the attention paid to financial protection.

Community First Choice – U.S.

In recognition of the number of people ineligible for LTC via Medicaid in the U.S., the CFC Program (45) was conceptualized as an opportunity for individual states to consider protecting more of their older residents with social care needs from income poverty. Hashiguchi found that in California, even with social care systems in place, "the out-of-pocket costs for older people with mean net wealth would be higher than their incomes" (p. 32), and that the costs of home care for those with median income, no net wealth, and low care needs would not be covered, highlighting the risk of income poverty among older adults. (30)

Initiative – Netherlands LTC Reform

The LTC insurance system in the Netherlands guarantees no older person experiences income poverty, despite OOP costs for low care needs (30), because copayments are means-tested to reduce the economic burden for the poor. (31) Municipalities that were unable to fund home-care recipients over the long term directed them to institutional care. Although institutional care would financially protect care recipients, those choosing to remain in their home risk their financial protection. To counteract this, municipalities have been able to negotiate substantial reductions in maximum co-payments since 2019. (52)

Financial sustainability in LTC

For peer review only

Seven reports (34, 36, 40, 42, 45, 49, 52) directly address financial sustainability and three (38, 39, 50) peripherally. Table 5 portrays financial sustainability in relation to the five initiatives.

Table 5: Financial Sustainability by Country and Initiative

Initiative	Country	Author, Year	Financial sustainability findings
LTCI pilot	China	Dai et al. 2022 (39)<br>Feng et al. 2021 (38)<br>Han & Shen 2022 (40)<br>Peng et al. 2022 (42)<br>Zhang et al., 2020 (34)<br>Zhou & Dai 2021[38]	• Integrating LTCI into the existing medical insurance framework is favored to reduce administrative costs, address basic LTC needs and gradually grow programs as financing permits. "The financial sustainability of the LTCI programmes has been questionable. A main reason for this is that the value allowed to be extracted from medical insurance foundations is limited." (39 p.196)<br>• Diversification of funding sources such as cash benefits to support family caregivers and in-kind services may be necessary based on pilot funding in some cities. Such diversification is offered as a path toward sustainability. (39)<br>• Glinskaya et al. advocates for the gradual increase in individual contributions in tandem with increased financial support from central and local governments to attain sustainability. (38)<br>• Funding sources affect sustainability and most cities have more than one source. Primary reliance is on transfers from the medical insurance fund, other sources include individual contributions, organizational contributions, financial subsidies, and social donations. (40)<br>• The Policy Modelling Consistency index was employed to measure the interaction coupling coordination degree between LTCI policy, population aging and economic development to assess the sustainability of the LTC pilot system. Shanghai reached the excellent level, Nantong achieved good coordination and the remaining cities' coordination degree was basic. The coordination degree between LTCI policy, population aging and economic development was reported as low or basic and suggests that economic development is not where it needs to be to sustain LTC financing. Sustained economic growth in tandem with population ageing and modifiable LTCI policies are essential to the financial sustainability of LTCI. (42)<br>• A general lack of system integration was identified and believed to place financial sustainability in jeopardy. (36)<br>• LCTI reduced informal/family caregiver care (34)<br>• Satisfaction with formal care was high. (34)<br>• Continued expansion of LTCI is favoured (34)
CareShield Life	Singapore	Fong & Borowski 2022 (49)	Policy analysis reported CareShield Life represents a collectivist (everyone contributes) approach to LTC Social Insurance; financial sustainability is guarded by rigorous claims criteria and eligibility assessments; inclusion of younger age groups contributes to affordability (coverage begins at age 30); and depth in service provision is sacrificed for breadth. Financial forecasting of the scheme is recommended to address sustainability.
LTC 2.0	Taiwan	Chiu et al. 2019 (50)	Examined difficulty with implementation to LTC 2.0 and challenges to sustainability.

For peer review only

Initiative	Country	Author, Year	Financial sustainability findings
LTCI reform	The Netherlands	Alders & Shut 2022 (52)	LTC reform initiated to address high institutional care costs. Reform focused on intensive home care as a substitute for nursing homes/institutional care. Social care was transferred to municipalities. The unanticipated effect was a cost-shifting by municipalities to the central government, which funds Public LTCI (specifically institutional care). This economic evaluation study examined cost shifting with a focus on the solvency rate of municipalities. Results: Municipalities with low solvency (20%) had 3.79 more admissions to Public LTCI per 10,000 population over age 65, equivalent to 2.5% more admissions than average. Urban municipalities with low financial solvency, a higher population over age 80 living alone or with limitations, and with a lower income had a significantly higher number of admissions to the Public LTCI system. Authors suggest shifting can be addressed by risk adjusting their social support funding based on the proportion of older people by municipality and on service needs. Such approaches optimize the use of funding sources and location of care and contribute to system sustainability.
Community First Choice	U.S.	Beauregard & Miller 2021 (45)	CFC was developed as an alternative to long-term institutional care. Participating states receive a 6% additional federal match to transition from Medicaid home and community-based services benefits. This comparative case study (2 states with CFC and 1 without) examined factors that influenced adoption. Factors included leadership from the state Medicaid office, ability to meet the 6% matching, LTC advocacy, ideology related to federal government involvement.<br>A state government advocate stated, "we have heard states really express concerns about the budgetary implications of this and whether it be sustainable even with the 6% increased FMAP [Federal Medical Assistance Percentage]."[p. 184]

LTCI=long-term care insurance; CFC=Community First Choice

Initiative – LTC Pilots China

Several studies (36, 40, 42) indicated challenges to the sustainability of China's LTCI pilots. The challenges reported were a lack of system integration (36), reliance on transfers from the medical insurance fund (39, 40), and a need for policy planning to address population aging and aligns with economic development. (42) Feng et al. (38) advocated for the gradual increase in individual contributions in tandem with increased financial support from central and local governments to attain sustainability. In addition, Zhang et al. (34) conducted a survey of care recipients and their family caregivers in Shanghai to determine the impact of LTCI on informal care hours provided by family members. Informal/family caregiver care was reduced by 0.47 hours. Across the 407 families, a weekly average decrease of 12.36 hours of informal care was

For peer review only

reported. Satisfaction with formal care was 72%. These results speak favorably for the LTCI pilots to alleviate the burden on carers and the continued expansion of LTCI.

Initiative – CareShield Life Singapore's LTCI

A policy analysis (49) of CareShield Life which is a mandatory scheme spreads risk across the age cohorts, while including a pre-funding component in which enrolees pay forward their future LTC needs, thus contributing to sustainability. It is estimated that less than 0.1% of the population aged 30-40 years have severe disabilities, therefore this insurance scheme is projected to not only meet LTC needs but also the needs of younger persons who may require services. These authors recognized the collectivist approach to this mandatory LTCI scheme, noting however that depth is sacrificed for breadth, and the need to conduct financial forecasting of the scheme to address sustainability.

Initiative – LTC 2.0 Taiwan

Chiu et al. addresses coverage peripherally in a qualitative descriptive study of the implementation of LTC 2.0. (50) They found that the mechanisms for service integration needed to be fully implemented and also recommended building trust between service delivery agencies and government, clarifying roles and responsibilities of government care managers and agency care managers, the development of an integrated information system, and the establishment of a budget tracking system.

Initiative – LTCI Reform the Netherlands

LTC reform (in 2015–2019) was initiated in response to high institutional care costs and focused on intensive home care as a substitute for nursing home/institutional care. Social care responsibility in the home was transferred to municipalities. Alders and Schut (52) conducted an economic evaluation of municipal-level data and estimated a random-effects model to explain

For peer review only

variation across municipalities and to report the numbers of people admitted to LTC institutions annually. The examination of municipality solvency was part of the evaluation. Municipalities with low solvency (20%) had 3.79 more admissions to Public LTCI per 10,000 population over age 65, equivalent to 2.5% more admissions than average. The authors suggest that shifting can be addressed by risk-adjusting their social support funding based on the proportion of older people by municipality and service needs. Such approaches optimize the use of funding sources and location of care and contribute to system sustainability.

Initiative – Community First Choice program in the U.S.

The CFC program offers a 6% federal increase in matching payments to shift spending from institutional to home-based care for individuals considered to be at an institutional level of care. Adoption and sustainability of the program are contingent upon federal home and community-based services policy that considers implications at the state level. Higher federal match rates and reducing the state share of Medicaid spending would create the conditions for expansion and sustainability. (46) A case study of two states with the CFC program (Maryland and Texas) and one state without (Oklahoma) (45) reported factors influencing the adoption of the CFC program included state ideology, fiscal status, advocacy for LTC, Medicaid leadership and the strength of existing home and community-based services.

DISCUSSION

We found that, apart from the CFC program, all initiatives either provided coverage to the entire population of the country or were working incrementally toward providing coverage to the entire population. Also common was the quest across initiatives to discern how to fund them. Older people with substantive care needs were the most likely to be eligible for services across all initiatives.

For peer review only

Although initiatives differed across the various countries, most entailed an increase in coverage. Strategies to increase coverage included expansion of eligibility criteria, reducing or waiving co-payments based on income, and simplifying processes for home support service providers. These strategies support aging in place as do initiatives aimed at health promotion that assist older people in maintaining if not improving their physical and cognitive abilities.

The findings related to financial protection include strategies such as tackling OOP expenses and providing home support services to support aging at home, mandating LTC, providing caregiver support and striking a balance between meeting LTC needs and how to fund them. These strategies saw a decrease in accessing both outpatient and inpatient medical services and access to LTC across the lifespan. A strategy that may help in the short and medium term is to develop an equitable LTC recipient dose sliding scale using ADLs, IADLs, cognition, behavioural changes, rehabilitation, home situation and caregiver stress. A sliding scale approach with individual contextualized person-focused assessments represents a strategy to push toward universalism, advance defamilization and restrain marketization (55) through optimal care to client matching. A sliding-scale approach calls for LTC systems that are dynamic and that can increase or decrease supports and services based on changes in individual needs.

Financial sustainability was a goal across all initiatives. Evidence of this was found in the evaluation of the LTCI pilots that stressed the need for system integration, dedicated funding, controlling OOP costs, and projecting LTC needs in relation to population aging and economic development. Furthermore, Singapore mandated LTC and structured the scheme to enroll everyone at age 30, paying premiums until age 67 and ensuring sustainability. Lastly the Netherlands reform altered the funding plan to cost share with municipalities. In doing so, they

For peer review only

discovered they needed to adjust the proportion of cost sharing based on the proportion of older people per municipality and service needs to address sustainability.

The findings of this review call forth the need to reflect on traditional approaches to LTC such as home care and institutional care. An alternate model could focus on enhanced services to support older adults to remain living in their homes and communities. A continuum that begins with remaining at home all the way to institutional care with more home-approximating options such as co-housing (56) may support optimal independence over a longer timeframe. This thinking is conceptually underpinned by the notion of providing the right support, in the right amount, at the right time to delay decline and to push down projections on the amount of care required, thereby contributing to financial sustainability. Such a continuum could contribute to positively addressing age-related inflation, which occurs when a group consumes more goods and services than it produces as they age. (57, 58) The control of the need for services combined with an increasing workforce dampens inflation and improves the economic growth required to sustain health and social initiatives. Health promotion can slow the decline in health thus delaying the need for supports and services.

LIMITATIONS

This rapid scoping review was limited to identifying initiatives (programs, policies or reforms) implemented and evaluated in the past five years, in LTC for people aged 60 and above. Countries need to include mechanisms within their initiatives to capture evaluation data. In the evidence assessed, LTC initiatives were focused on launching as opposed to evaluation, and we expect to see more evaluation including quality addressed in future research. Undoubtedly there are initiatives that do not appear in this report because they did not meet all the inclusion criteria. In addition, there is commonly a lag between initiatives, their evaluation and subsequent

For peer review only

publication. There is always the possibility that, despite the number of database searches conducted, the keywords used in the searches may not have been used in a relevant publication. Many countries have long-established LTC programs; however, unless a major modification occurred and met the inclusion criteria for this review, it would not have been captured. Some of the included reports analyzed textual data from policy data in the absence of implementation data.

RESEARCH GAPS

We believe there are recent LTC initiatives in place that have not been evaluated or have been evaluated but not published, and that such evidence needs to be published for all countries/sub-nations to benefit from. The number of economic evaluations of LTC initiatives was limited, and such evaluations should be encouraged to inform evolution in LTC. In addition to evaluation implementation, data from the initiatives is important to know what works and where improvements are needed to contribute to sustainability.

Although it may have been the intent of some researchers to capture equity measures, this was not necessarily made explicit in their reports. Absent from the data are race, religion, and identification of any gender minority groups. Discussion relating the characteristics to study results/findings was limited. Consistent use of a conceptual framework to compare policies such as that proposed by Noda et al. (53), is suggested to inform comparative evaluation studies or single initiative evaluation.

CONCLUSION

The initiatives related to coverage, financial protection and financial sustainability identified in this review came from high, upper-middle, lower-middle and low-income[13] and hold hope for the growth of existing initiatives and their expansion within and beyond their respective

For peer review only

countries. The ones pertaining to lower-middle and low-income economies in particular fill a gap in a body of literature that has focused on high-income countries. Initiatives where LTCI is mandatory and accompanied by a commensurate funding scheme are situated to facilitate older people ageing in place. Efforts to expand population coverage are common across the initiatives, with the potential for wider economic benefits. In addition, initiatives that enable older people to access services while avoiding OOP-induced poverty contribute to improved health and well-being. Preserving health in older people longer may alleviate downstream costs and contribute to financial sustainability. The relevance and timeliness of LTC initiatives cannot be understated because by 2050, 80% of older people will live in low and middle-income countries. (59)

ETHICS STATEMENTS

Patient consent for publication

Not applicable.

Ethics approval

Ethics approval not required in this rapid scoping review.

DATA AVAILABILITY STATEMENT

All search strategies are available via searchRxiv (see online supplemental file). All additional data relevant to the study are included as supplementary information.

ACKNOWLEDGEMENTS

The authors are grateful to Dalhousie University Librarian Louise Gillis, who completed the peer review of the search strategy.

For peer review only

References

1. United Nations Department of Economic and Social Affairs. World population prospects 2022: Summary of results. 2022. https://www.un.org/development/desa/pd/sites/www.un.org.development.desa.pd/files/undesa_pd_2022_wpp_key-messages.pdf.

2. Costa-Font J, Courbage C, Swartz K. Financing long-term care: Ex ante, ex post or both? Health Econ 2015;24(S1):45-57. doi: 10.1002/hec.3152

3. Grignon M, Spencer BG. The funding of long-term care in Canada: What do we know, what should we know? Can J Aging 2018;37(2):110-20. doi: 10.1017/S0714980818000028

4. OECD. Spending on long-term care. 2020. https://www.oecd.org/health/health-systems/Spending-on-long-term-care-Brief-November-2020.pdf.

5. Feng Z, Glinskaya E. Aiming Higher: Advancing public social insurance for longterm care to meet the global aging challenge; Comment on "Financing long-term care: Lessons from Japan". Int J Health Policy Manag 2020;9(8):356-59. doi: 10.15171/ijhpm.2019.121

6. Chen L, Zhang L, Xu X. Review of evolution of the public long-term care insurance (LTCI) system in different countries: Influence and challenge. BMC Health Services Research 2020;20:1057. doi: 10.1186/s12913-020-05878-z

7. Hosseini Jebeli SS, Aziz R, Megumi R, et al. Measuring universal health coverage to ensure continuing care for older people: A scoping review with specific implications for the Iranian context. Eastern Mediterranean Health Journal 2021;27(8):806-17. https://apps.who.int/iris/handle/10665/353217.

8. World Health Organization. Framework for countries to achieve an integrated continuum of long-term care. 2021. https://apps.who.int/iris/handle/10665/349911.

9. World Health Organization. Financial protection. https://www.who.int/health-topics/financial-protection.

10. World Health Organization. SDG Target 3.8: Achieve universal health coverage, including financial risk protection, access to quality essential health-care services and access to safe, effective, quality and affordable essential medicines and vaccines for all. https://www.who.int/data/gho/data/themes/topics/indicator-groups/indicator-group-details/GHO/sdg-target-3.8-achieve-universal-health-coverage-(uhc)-including-financial-risk-protection.

11. Colombo F, Llena-Nozal A, Mercier J, et al. Help wanted? Providing and paying for long-term care. 2011. https://www.oecd-ilibrary.org/content/publication/9789264097759-en.

For peer review only

12. Peters M, Godfrey C, McInerney P, et al. Chapter 11: Scoping Reviews (2020 version). In: Aromataris E, Munn, Z, eds. JBI Manual for Evidence Synthesis 2020. doi: 10.46658/JBIMES-20-12

13. Tricco A, Lillie, E, Zarin, W, et al. PRISMA extension for scoping reviews (PRISMA-ScR): Checklist and explanation. Annals of Internal Medicine 2018;169(7):467-73. doi: 10.7326/m18-0850

14. Tricco AC, Langlois EV, Straus SE. Rapid reviews to strengthen health policy and systems: A practical guide. Geneva: World Health Organization 2017. https://apps.who.int/iris/bitstream/handle/10665/258698/9789241512763-eng.pdf.

15. Tricco AC, Zarin W, Clement F, et al. Introducing the Strategy for Patient Oriented Research (SPOR) Evidence Alliance: A partnership between researchers, patients and health system decision-makers to support rapid-learning and responsive health systems in Canada and beyond. Facets 2022;7:639-53. doi: 10.1139/facets-2021-0127

16. Lunny C, Zarin W, Chaudhry S, et al. An inclusive and diverse governance structure of the strategy for patient-oriented research (SPOR) Evidence Alliance. Facets 2022;7:435-47. doi: 10.1139/facets-2021-0129

17. Zarin W, Lunny C, Chaudhry S, et al. A Canadian model for providing high-quality, timely and relevant evidence to meet health system decision-maker needs: The SPOR Evidence Alliance. Facets 2022;7:420-34. doi: 10.1139/facets-2021-0132

18. Li LC, Hoens AM, Wilhelm L, et al. Patient engagement in the SPOR Evidence Alliance: Reflection and learnings. Facets 2022;7:126-38. doi: 10.1139/facets-2021-0133

19. McGowan J, Sampson M, Salzwedel DM, et al. PRESS peer review of electronic search strategies: 2015 guideline statement. Journal of Clinical Epidemiology 2016;75:40-6. doi: 10.1016/j.jclinepi.2016.01.021

20. Rethlefsen ML, Kirtley S, Waffenschmidt S, et al. PRISMA-S: An extension to the PRISMA statement for reporting literature searches in systematic reviews. Syst Rev 2021;10(1):39. doi: 10.1186/s13643-020-01542-z

21. Pham B, Rios P, Radhakrishnan A, et al. Comparative-effectiveness research of COVID-19 treatment: A rapid scoping review. BMJ Open 2022;12(6):e045115. doi: 10.1136/bmjopen-2020-045115

22. Rios P, Radhakrishnan A, Williams C, et al. Preventing the transmission of COVID-19 and other coronaviruses in older adults aged 60 years and above living in long-term care: A rapid review. Syst Rev 2020;9(1):218. doi: 10.1186/s13643-020-01486-4

23. Page MJ, McKenzie JE, Bossuyt PM, et al. The PRISMA 2020 statement: An updated guideline for reporting systematic reviews. BMJ 2021;372:n71. doi: 10.1136/bmj.n71

For peer review only

24. The Campbell and Cochrane Equity Methods Group. PROGRESS-Plus. https://methods.cochrane.org/equity/projects/evidence-equity/progress-plus.

25. O'Neill J, Tabish H, Welch V, et al. Applying an equity lens to interventions: Using PROGRESS ensures consideration of socially stratifying factors to illuminate inequities in health. Journal of Clinical Epidemiology 2014;67(1):56-64. doi: 10.1016/j.jclinepi.2013.08.005

26. Gomersall JS, Jadotte YT, Xue Y, et al. Conducting systematic reviews of economic evaluations. Int J Evid Based Healthc 2015;13(3):170-78. doi: 10.1097/xeb.0000000000000063

27. Moola S, Munn Z, Tufanaru C, et al. Chapter 7: Systematic reviews of etiology and risk. In: Aromataris E, Munn, Z eds. JBI Manual for Evidence Synthesis 2020. https://synthesismanual.jbi.global.

28. Tufanaru C, Munn, Z, Aromataris, E, Campbell, J, Hopp, L. Chapter 3: Systematic reviews of effectiveness. In: Aromataris E, Munn, Z eds. JBI Manual for Evidence Synthesis 2020. https://synthesismanual.jbi.global.

29. National Collaborating Centre for Methods and Tools. Health evidence quality assessment tool. 2018. https://www.nccmt.ca/knowledge-repositories/search/275.

30. Hashiguchi TCO, Llena-Nozal A. The effectiveness of social protection for long-term care in old age: Is social protection reducing the risk of poverty associated with care needs? OECD Publishing 2020. https://EconPapers.repec.org/RePEc:oec:elsaad:117-en.

31. Kotschy R, Bloom D. A comparative perspective on long-term care systems. 2022; 75(3-4): 47-69. doi: 10.1111/issr.12307

32. Lei X, Bai C, Hong J, et al. Long-term care insurance and the well-being of older adults and their families: Evidence from China. Social Science & Medicine 2022;296:114745. doi: 10.1016/j.socscimed.2022.114745

33. Chen L, Zhang X, Xu X. Health insurance and long-term care services for the disabled elderly in China: Based on CHARLS data. Risk Management and Healthcare Policy 2020;13:155-62. doi: 10.2147/RMHP.S233949

34. Zhang H, Zhang X, Zhao Y, et al. Impact of formal care use on informal care from children after the launch of long-term care insurance in Shanghai, China. International Journal of Environmental Research and Public Health 2020;17(8): 2938. doi: 10.3390/ijerph17082938

35. Zhang Y, Yu X. Evaluation of long-term care insurance policy in Chinese pilot cities. International Journal of Environmental Research and Public Health 2019;16(20): 3826. doi: 10.3390/ijerph16203826

For peer review only

36. Zhou W, Dai W. Shifting from fragmentation to integration: A systematic analysis of long-term care insurance policies in China. International Journal of Integrated Care 2021;21(3):11. doi: 10.5334/ijic.5676

37. Wu J, Chen S, Wen H, et al. Health status, care needs, and assessment for beneficiaries with or without dementia in a public long-term care insurance pilot in Guangzhou, China. BMC Health Services Research 2020;20(1):1127. doi: 10.1186/s12913-020-05965-1

38. Feng Z, Zhuang X, Glinskaya E. Understanding China's long-term care insurance pilots: Key features, challenges and prospects. China: An International Journal 2021;19(3):168-87. doi: 10.1353/chn.2021.0034

39. Dai W, Li Y, Shen J. The pilot programme of long-term care insurance in China: Fragmentation and policy implications. China: An International Journal 2022;20(2):186-206. doi: 10.1353/chn.2022.0010

40. Han Y, Shen T. Long-term care insurance pilot programme in China: Policy evaluation and optimization options-taking the pilot programme in the northeast of China as an example. International Journal of Environmental Research and Public Health 2022;19(7): 4298. doi: 10.3390/ijerph19074298

41. Chen H, Ning J. The impacts of long-term care insurance on health care utilization and expenditure: Evidence from China. Health Policy and Planning 2022;37(6):717-27. doi: 10.1093/heapol/czac003

42. Peng R, Deng X, Xia Y, et al. Assessing the sustainability of long-term care insurance systems based on a policy-population-economy complex System: The case study of China. International Journal of Environmental Research and Public Health 2022;19(11):6554. doi: 10.3390/ijerph19116554

43. Tang Y, Chen T, Zhao Y, et al. The Impact of the long-term care insurance on the medical expenses and health status in China. Frontiers in Public Health 2022;10:847822. doi: 10.3389/fpubh.2022.847822

44. Liu H, Hu T. Evaluating the long-term care insurance policy from medical expenses and health security equity perspective: Evidence from China. Archives of Public Health 2022;80:3. doi: 10.1186/s13690-021-00761-7

45. Beauregard LK, Miller EA. A comparative analysis of state adoption of the Community First Choice program. Home Health Care Services Quarterly 2021;40(3):177-91. doi: 10.1080/01621424.2021.1947926

46. Koumoutzis A, Heston-Mullins J, Mayberry PS, et al. If you ask them, they will support: A national study of local initiatives developed to provide social care to older adults in the community. Journal of Applied Gerontology 2022;41(8):1860-69. doi: 10.1177/07334648221090945

For peer review only

47. Koumoutzis A, Stemen SE, Maharjan R, et al. Local initiatives to fund services for older americans: community recognition of the importance of social care. Journal of Applied Gerontology 2021;40(9):980-84. doi: 10.1177/0733464820944325

48. Sum G, Kadir MM, Ho SH, et al. Cost analysis of a Patient-Centred Medical Home for community-dwelling older adults with complex needs in Singapore. Annals Academy of Medicine Singapore 2022;51(9):553-66. doi: 10.47102/annals-acadmedsg.2022165

49. Fong JH, Borowski A. Long-term care insurance reform in Singapore. Journal of Aging & Social Policy 2022;34(1):73-90. doi: 10.1080/08959420.2021.1926867

50. Chiu T-Y, Yu H-W, Goto R, et al. From fragmentation toward integration: A preliminary study of a new long-term care policy in a fast-aging country. BMC Geriatrics 2019;19(1):159. doi: 10.1186/s12877-019-1172-5

51. Chen C-F, Fu T-H. Policies and transformation of long-term care system in Taiwan. Annals of Geriatric Medicine and Research 2020;24(3):187-94. doi: 10.4235/agmr.20.0038

52. Alders P, Schut FT. Strategic cost-shifting in long-term care: Evidence from the Netherlands. Health Policy 2022;126(1):43-48. doi: 10.1016/j.healthpol.2021.11.008

53. Noda S, Hernandez PMR, Sudo K, et al. Service delivery reforms for Asian ageing societies: A cross-country study between Japan, South Korea, China, Thailand, Indonesia, and the Philippines. International Journal of Integrated Care 2021;21(2):1. doi: 10.5334/ijic.4739

54. Kotschy R, Bloom D. A comparative perspective on long-term care systems [Working paper 29951]. National Bureau of Economic Research 2022. doi: 10.3386/w29951

55. Ranci C, Pavolini E. Not all that glitters is gold: Long-term care reforms in the last two decades in Europe. Journal of European Social Policy 2015;25(3):270-85. doi: 10.1177/0958928715588704

56. Canadian Cohousing Network. Home. https://cohousing.ca/.

57. Petersen T, López L. Demographics and foreign trade: What are the effects of an aging population on the current account balance of an economy? Bertelsmann Stiftung 2019. https://globaleurope.eu/globalization/effects-of-aging-population/.

58. Yoshino N, Kim CJ, Sirivunnabood P. Aging population and its impacts on fiscal sustainability. T20 Japan 2019. https://t20japan.org/policy-brief-aging-population-impacts-fiscal-sustainability/.

59. World Health Organization. Ageing and Health 2022. https://www.who.int/news-room/fact-sheets/detail/ageing-and-health.

For peer review only

FOOTNOTES

Contributors: Design of the review: MM, LEW, ACT, EL, BP, GVC, MRG, BP, SS, CL, TK, ARY; Data collection: MM, LEW, EL, AM, MI, BP, JC; Critical appraisal: MM, LEW, BP; Data analysis and interpretation: MM; Drafting the article: MM, EL, JC, SR, MXM; Critical revisions of the article: MM, LEW, EL, JC, ACT, BP, SS, SM, WI, GVC, MRG, ARY, AM, MI, CL, JW, SS (NSH), TK, DL, JC, EM, RR, SR, MXM. All authors approved the final version to be published.

Funding: This research was supported by the World Health Organization Centre for Health Development (WHO Kobe Centre – WKC: K22005).

Competing interests statement: LEW is the co-owner of an assisted living home.

Patient and public involvement: Public partners attended team meetings and provided input into the design of the study. They also provided input and feedback on drafts of tools, reports, and the manuscript, as well as dissemination activities.

Figure 1: PRISMA flow diagram of article selection

[1] Three reviewers initially independently screened 500 records using CAL® and a combined screening sample of 1970 for a total screening sample of 3470 records. With an estimate of duplicate records of 8.4% among the first 1500, we estimate humans screened a total of 3179 records using CAL®.

For peer review only

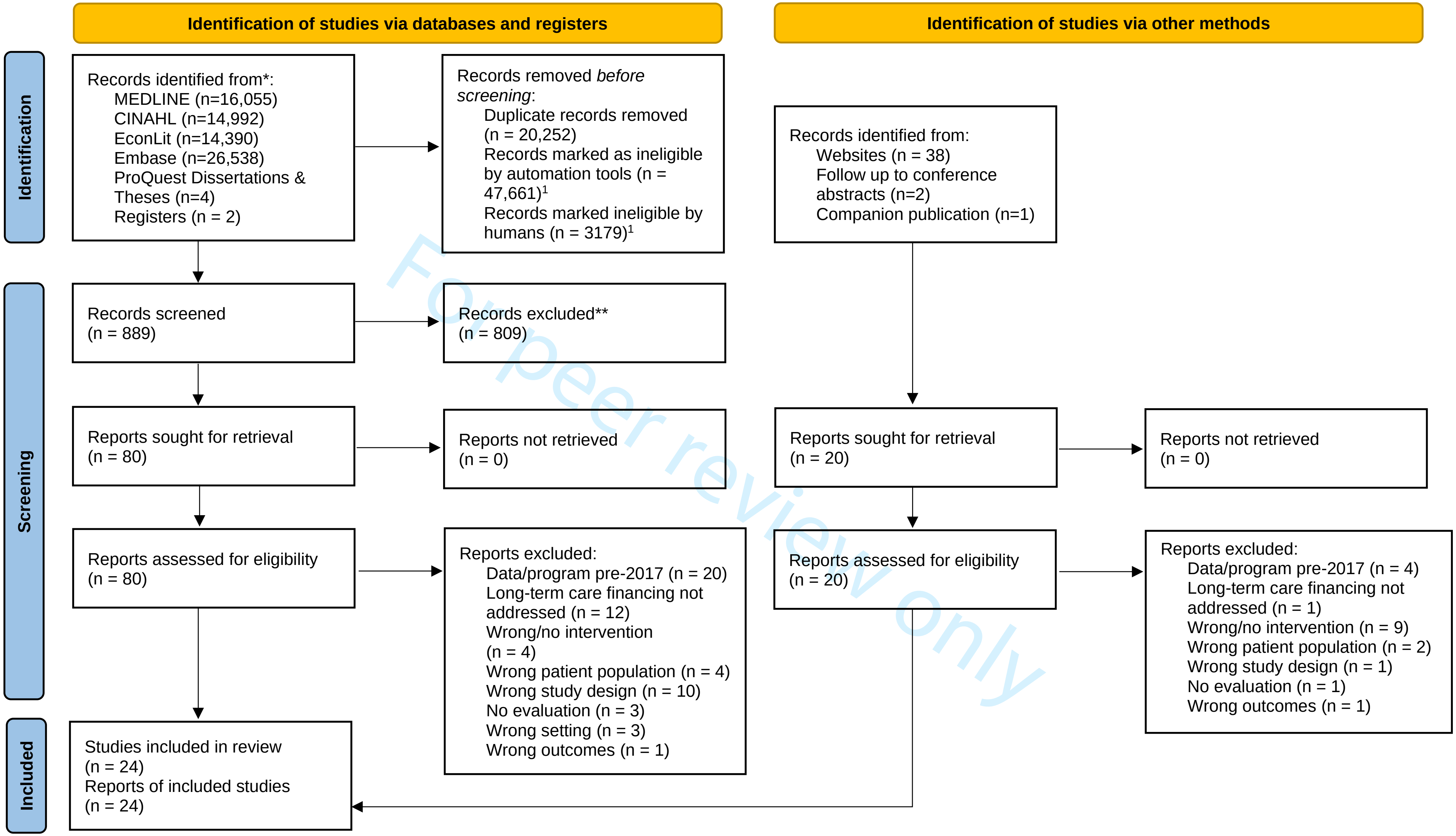

For peer review only

Table 1: Study Characteristics

Author Year	Country	Initiative	Design	Aim	Population and Data Sources	Quality Appraisal
Alders & Shut 2022(54)	The Netherlands	LTCI reform	Economic evaluation	To discern if municipalities with a low solvency rate (<20 percent) are more likely to engage in strategic cost shifting and if this resulted in an overall upward trend in admission rates to LTC services covered by public LTC services.	People aged 65+ in the Netherlands; data from 327 out of 355 municipalities from 2014–2019.<br><br>Data source(s): Monitor Long-term Care (Monitor Langdurige Zorg); Online dashboard by the Ministry of the Interior; Statistics Netherlands	Medium
Beauregard & Miller 2021(47)	U.S.	Community First Choice program	Comparative case study (qualitative)	To understand state processes related to the adoption of the Medicaid Community First Choice (CFC) Program and to identify factors that influenced states' decisions to adopt CFC.	LTC population: Medicaid-eligible older adults in Texas, Oklahoma, Maryland, and nationally (U.S.).<br><br>Study population: Data collected from n=46 individuals in roles as follows: federal and state bureaucrats, consumer and provider advocacy groups, consultants, and policy experts. Maryland (n=14), Texas (n=14), Oklahoma (n=9), and national (n=9) level participants.<br><br>Data source(s): Interviews	Medium
Chen et al. 2020(35)	China	LTCI pilots	Cross-sectional study	To evaluate the influence of health insurance and other factors on the availability of LTC services.	n=7787; Urban: n=3945; Rural: n=3842; CHARLS (2015) database adults aged 45 and up; the sample is aged 60 and up; CHARLS (2015) covered more than 10,000 families and 17,500 individuals from 150 districts of 28 provinces.<br><br>Data source(s): China Health and Retirement Longitudinal Study (CHARLS) database, 2015	Medium
Chen & Fu 2020(53)	Taiwan	LTC 2.0 reform	Policy analysis	To review the development of LTC policies, present strategies for expanding LTC, and outline policy suggestions and implications.	Older adults in Taiwan. The proportion of the population aged 65 years and over in Taiwan has doubled from 7% in 1993 to 14% in 2018. The number of older adults receiving LTC services increased from 9,148 in 2008 to	High

For peer review only

Author Year	Country	Initiative	Design	Aim	Population and Data Sources	Quality Appraisal
					258,351 in 2019, which only accounts for 41.47% of the population needing LTC.<br><br>Data source(s): Policy documents; government data	
Chen & Ning 2022(43)	China	LTCI pilot	Quasi-experimental design	To examine the policy treatment effect of long-term care insurance (LTCI) on health care utilization and OOP health expenditure (OOP) in China.	N=20,814<br>Participants in the China Health and Retirement Longitudinal Study (CHARLS).<br><br>Data source(s): China Health and Retirement Longitudinal Study (CHARLS) longitudinal study, 2018	High
Chiu et al. 2019(52)	Taiwan	LTC 2.0 reform	Qualitative research	To examine the experiences and challenges of integration for LTC plan 2.0 agencies in Taiwan	LTC population: Older adults in 20 counties where LTC Plan 2.0 was being piloted in Taiwan in early 2017.<br><br>Study population: Interviews with 6 people working in 3 LTC Plan 2.0 Tier A agencies in north, west, and central Taiwan, including a chief executive officer (CEO) or executive director (ED) and a case manager at each agency.<br><br>Data source(s): Interviews	Medium
Dai et al. 2022(41)	China	LTCI pilot	Systematic literature review of LTC policies and implementation	This study examines the LTCI pilot in China and compares it to other LTCI programs in Germany, Japan, and South Korea through a systematic literature review and discusses the implications of its implementation in a national framework.	Adults in one of the 49 expanded pilot cities in China.<br><br>Data source(s): National and local government websites; China National Knowledge Infrastructure, Baidu Scholar and international databases	High
Feng et al. 2021(40)	China	LTCI pilot	Policy analysis	To provide a timely review and assessment of China's ongoing LTCI programs	People over 60 who meet the eligibility requirement for the LTCI pilot and who live in one of the 15 pilot cities.	High

For peer review only - http://bmjopen.bmj.com/site/about/guidelines.xhtml

Author Year	Country	Initiative	Design	Aim	Population and Data Sources	Quality Appraisal
				across the initial 15 pilot cities. The focus is on synthesizing key LTCI program features across all pilot sites, not on profiling pilot sites, not on profiling and analyzing individual pilots in depth.	Data source(s): Policy documents; published research articles; grey literature; LTCI Policy Database, School of Public Health, Peking University	
Fong & Borowski 2022(51)	Singapore	CareShield Life	Policy analysis	To analyze the 2019 long-term care social insurance program, including successes and challenges, goals, design, features, financing arrangements, and initial assessment.	Older adults in Singapore. The proportion of those aged 65 and older increased x3 from 4.9% in 1980 to 14.5% in 2019.<br><br>Data source(s): Not reported. The reference list includes government documents and websites.	High
Han & Shen 2022(42)	China	LTCI pilot	Qualitative research	To explore the common problems in policy practices and to provide recommendations for further expansion of the LTCI pilot.	LTC population: China's adult population ≥60, which accounts for 18.7% of the total population.<br><br>Study population: n=10 participants, including managers (n=4), caregivers (n=2), a medical staff member (n=1), and older adults with disabilities (n=3)<br><br>Data source(s): In-depth interviews	High
Hashiguchi & Llena-Nozal 2020(32)	25 out of 41 OECD countries and EU Member States*	Public social protection systems for LTC	Comparative case study	To provide a novel set of comprehensive and internationally comparable estimates of the adequacy, equity and efficiency of public social protection systems for LTC in old age in OECD countries and EU Member States.	People 65 and older in the selected 25 OECD countries and EU Member States*.<br><br>Data source(s): Survey of Health, Ageing and Retirement in Europe (SHARE) Wave 7	N/A

For peer review only

Author Year	Country	Initiative	Design	Aim	Population and Data Sources	Quality Appraisal
Kotschy & Bloom 2022(33)	Germany, Israel, Japan, the Netherlands, South Korea	LTC systems comparison	Policy analysis	To investigate the challenges that population aging poses to long-term care.	Adults aged 65+ in Germany, Israel, Japan, the Netherlands, and South Korea<br><br>Data source(s): Survey of Health, Ageing and Retirement in Europe (SHARE) wave 7 and 8; Health and Retirement Study (HRS) wave 13; English Longitudinal Study of Ageing (ELSA) wave 8	N/A
Koumoutzis et al. 2021(49)	U.S.	Local initiatives to fund social care services	Qualitative research	To examine how communities across the nation are utilizing local funding streams to support aging services.	LTC population: Adults 60+ in the U.S.<br><br>Study population: NR<br><br>Data source(s): 2019 Advancing States national survey of State Units on Aging; telephone calls (State Units on Aging, Area Agencies on Aging, secretary of state's offices, county clerk's offices, and other officials involved in local funding efforts); web searches	Medium
Koumoutzis et al. 2022(48)	U.S.	Community LTC initiatives to support ageing	Survey	To study community initiatives developed to provide services to older adults through locally generated tax revenues and provide insight into their caregiver support efforts.	LTC population: Older adults in the U.S.<br><br>Study population: Representatives from 228 organizations across 15 states, delivering LTC services.<br><br>Data source(s): Phase 1: 2019 Advancing States national survey of state units on aging; telephone calls (telephone calls to state units on aging, area agencies on aging, aging services providers, county auditor offices, and Secretary of State offices); online reviews and web searches. Phase 2: Qualtrics survey to local funding organizations	High
Lei et al. 2022(34)	China	LTCI pilot	Quasi-experimental design	To evaluate the impact of LTCI on the covered populations in the LTCI pilots across different cities from 2015 to 2017, and to study	Older adults (aged 65+) from 152 cities: n=3,423<br>Treatment group (coverage): n=187<br>Control group (no coverage): n=3,236.	High

For peer review only

Author Year	Country	Initiative	Design	Aim	Population and Data Sources	Quality Appraisal
				various outcomes (including older adults' ADL-related need for care, family burden, medical expenditures, and some important health outcomes) among covered older adults and their families.	Data source(s): Chinese Longitudinal Healthy Longevity Survey (CLHLS)	
Liu & Hu 2022(46)	China	LTCI pilot	Quasi-experimental design	To investigate the impact of the policy pilot on the income fairness of the group system and to comprehensively analyze the institutional effect of the LTCI policy pilot.	People aged 60 and over living in one of the 15 LTCI pilot areas (n=25,063) included in CHARLS.<br><br>Data source(s): China Health and Retirement Longitudinal Study (CHARLS) database, 2013, 2015, 2018 (valid samples with a 3-year period)	High
Noda et al. 2021(55)	Japan, Korea, Thailand, China, Indonesia, the Philippines	Healthcare service delivery reform policies for the ageing	Policy comparison	To compare healthcare service delivery reform policies for older adults in Japan, Korea, Thailand, China, Indonesia and the Philippines.	LTC population: Older adults in Japan, Korea, Thailand, China, Indonesia, and the Philippines.<br><br>Study population: Health officials of provincial health and welfare departments, and service providers such as doctors and LTC providers (no n provided).<br><br>Data source(s): Document reviews; interviews with key informants	Medium
Peng et al. 2022(44)	China	LTCI pilot	Policy analysis	To assess the sustainability of the LTCI system in China using a policy population economics (PPE) system.	Population of 14 pilot cities in China. Due to a lack of data, one pilot city, Shihezi, was excluded.<br><br>Data source(s): Policy documents; China Statistical Yearbook 2020; the China City Statistical Yearbook; the Chinese National Economy and Social Development Statistics Bulletin	N/A

For peer review only

Author Year	Country	Initiative	Design	Aim	Population and Data Sources	Quality Appraisal
Sum et al. 2022(50)	Singapore	Integrated Patient-Centred Medical Home (PCMH) model	Quasi-experimental design	To address gaps in the literature on the evaluation of costs associated with the implementation of an integrated PCMH model for community-dwelling older adults with complex needs.	Patients with high biopsychosocial health risk, aged ≥40, who resided in Whampoa (n=165)<br><br>Data source(s): Client Service Receipt Inventory (CSRI) survey	High
Tang et al. 2022(45)	China	LTCI pilot	Quasi-experimental design	To evaluate the implementation effect of the Long-Term Care Insurance (LTCI) policy, and its impact on the medical expenses and the health status of the middle-aged and elder population.	Families with people middle-aged (≥45) and older in any of the 12 cities included in the CHARLS database (n=42,591)<br><br>Data source(s): China Health and Retirement Longitudinal Study (CHARLS)	Medium
Wu et al. 2020(39)	China	LTCI pilot	Retrospective cross-sectional study	To evaluate the LTCI pilot by exploring the characteristics and care needs of claimants, and performance of the assessment tool.	Claimants: n=4810; Individuals who received benefits: n=4582;<br>Aged ≥60 years: n=4357;<br>Beneficiaries without dementia: n=3791;<br>Beneficiaries with dementia: n=791;<br>Unsuccessful claimants without dementia: n=206;<br>Unsuccessful claimants with dementia: n=22<br><br>Data source(s): Claims data	Medium
Zhang & Yu 2019(37)	China	LTCI pilot	Quasi-experimental design	To explore the outcomes and evaluate the performance of the LTCI policy in the Chinese pilot cities and estimate the willingness of Chinese citizens to expand the formal implementation of LTCI policy in China.	Older adults engaged in the implementation process of the LTCI pilots in 15 pilot cities (n=1,167)<br>Elite politicians (n=5)<br><br>Data source(s): Survey questionnaire; interviews	Medium

For peer review only

Author Year	Country	Initiative	Design	Aim	Population and Data Sources	Quality Appraisal
Zhang et al. 2020(36)	China	LTCI pilot	Quasi-experimental design	To examine how the use of formal care impacts the use of informal care in Shanghai, which was one of China's first long-term insurance pilots in 2016.	Families in Shanghai that include an older adult (≥60) who had used formal care provided by LTCI for 1 to 3 months and a child who is primarily responsible for daily informal care.<br>Care recipients: n=407<br>Caregivers n=407<br><br>Data source(s): Interview-based surveys	Medium
Zhou & Dai 2021(38)	China	LTCI pilot	Systematic literature review of LTC policies	To review and assess the performance and effectiveness of the LTCI policy regime in China.	Older adults in one of the 15 pilot cities.<br><br>Data source(s): Policy documents	Medium

*The countries and subnational areas covered include Austria (Vienna region), Belgium (Flanders), Canada (Ontario), Croatia, Czech Republic, England, Estonia (Tallinn), Finland, France, Germany, Hungary, Iceland (Reykjavik), Ireland, Italy (South Tyrol), Japan, Korea, Latvia, Lithuania, Luxembourg, Slovak Republic, Slovenia, Spain, Sweden, the Netherlands, the United States of America (Illinois and California).
Quality appraisal (score % as per # of tool items): high quality=80-100%; medium quality=50-79%; low quality= <50%

For peer review only

Search Strategies

Search strategies are available via searchRxiv for the following information sources:

- MEDLINE: https://doi.org/10.1079/searchRxiv.2023.00213
- CINAHL: https://doi.org/10.1079/searchRxiv.2023.00214
- EconLit: https://doi.org/10.1079/searchRxiv.2023.00216
- Embase: https://doi.org/10.1079/searchRxiv.2023.00217
- ProQuest Dissertations and Theses: https://doi.org/10.1079/searchRxiv.2023.00220
- WHO ICTRP: https://doi.org/10.1079/searchRxiv.2023.00219
- ClinicalTrials.gov: https://doi.org/10.1079/searchRxiv.2023.00218

CADTH Grey Matters

All websites included in the Health Economics category of CADTH Grey Matters were searched or browsed for relevant records:

- Centre for Health Economics and Policy Analysis (McMaster University)
- Centre for Reviews and Dissemination (University of York)
- Economic Burden of Illness in Canada (Public Health Agency of Canada)
- Health Economics Research Unit (University of Aberdeen)
- IDEAS/RePEc database
- IHE publications (Institute of Health Economics, Canada)
- National Quality Measures Clearinghouse (Agency for Healthcare Research and Quality, U.S.)
- OHE publications (Office of Health Economics, United Kingdom)
- Ontario Case Costing Initiative (Ontario Ministry of Health and Long-Term Care)
- THETA Publications and Knowledge Translation to Policy (KT) Activities (Toronto Health Economics and Technology Assessment Collaborative)

If the website had a search feature, the search was as follows: ("long-term care" OR "long term care")

If there were too many results, it was narrowed by adding the following: AND (financing OR funding)

Results: 34 (38 minus 4 duplicates)

For peer review only

Data extraction form

General information

Study ID

Enter the Covidence record number here.

Example: #34

Title

Title of paper/abstract/report

Last name of lead author

Characteristics of study

Publication type

If publication type is "other" please specify the details below.

☐Journal article

☐Dissertation/thesis

☐Report

☐Other Click or tap here to enter text.

Aim/purpose/objective of study

For peer review only

Enter the research question or hypothesis.

Study design

Studies might not always explicitly state the design, in which case select the best option based on the methods section. If unclear, please include any relevant information in the notes section below.

☐Randomized controlled trial

☐Non-randomized experimental study

☐Quasi-experimental design

☐Cohort study

☐Cross-sectional study

☐Case–control study

☐Case series

☐Case report

☐Economic evaluation

☐Comparative case study

☐Policy analysis

☐Qualitative research (specific approach)

☐Other Click or tap here to enter text.

Country/setting

For peer review only

If the study is comparing countries/settings, ensure all the countries are listed, separated by semicolons. If the study does not mention the country but mentions a region, enter the region.

Population description

Describe the overall population. For more than one population start a new paragraph.

Gender

Enter the breakdown of population gender if reported.

Sexual orientation

Enter the breakdown of population sexuality if reported.

Race

Enter the breakdown of population racial composition if reported.

Age

Enter the overall age of population if reported. If age is reported as a range (e.g. 35–50 years) enter the relevant range and accompanying value with a colon in between (e.g. 60–79: 50%). If there are multiple ranges, separate using a semicolon (e.g. 60–79: 50%; 80–99: 50%).

Place of residence

Enter the breakdown of places of residence (e.g. rural, urban, suburban, long-term care [LTC] congregate setting, etc.) if reported.

For peer review only

Occupation

Enter the breakdown of occupations if reported.

Religious backgrounds

Enter the breakdown of religious backgrounds if reported.

Education

Enter the breakdown of educational backgrounds if reported.

Socioeconomic status

Enter the breakdown of socioeconomic status if reported.

Social capital

Social capital refers to social relationships, networks and social support. Enter information related to the social capital of the population if reported.

Disability

Enter the breakdown of the population disability categories if reported.

LTC financing

LTC system description

Describe the overall LTC system. For more than one system start a new paragraph.

For peer review only

Financing description

Describe how the LTC system is financed. For more than one system start a new paragraph.

Funding mechanism

Enter the details of the funding mechanism(s) in the relevant column. Enter NA in boxes where the funding mechanism is not applicable. Include the percentage mix for each funding mechanism if reported. If not reported, enter NR.

Funding mechanism	Details	Percentage mix
Guarantees		
Insurance		
Fees for service		
Loans		
Repayable contributions		
Result-based financing		
Equity		
Bonds		
Charitable donations		
Local government subsidies		
Taxes		
Co-payment by users		
Central government subsidies		

For peer review only

Other		

Service description

Describe the services covered under the programme policy reform or initiative if reported.

Coverage

How does the initiative/programme/policy/reform address LTC coverage? Enter NA if not addressed.

Quality

How does the initiative/programme/policy/reform address LTC quality? Enter NA if not addressed.

Financial protection

How does the initiative/programme/policy/reform address LTC financial protection? Enter NA if not addressed.

Financial sustainability

How does the initiative/program/policy/reform address LTC financial sustainability? Enter NA if not addressed.

Empirical evaluation

Intervention

What is the initiative/programme/policy/reform?

For peer review only

Comparator

What is the initiative/programme/policy/reform being compared to? If there is no comparator enter NA.

Intervention time frame

What is the time frame of the intervention?

Examples: policy put in place in June of 2017; pilot project from January 2017 to August 2019.

Data sources

Enter the sources of data. If there are multiple sources separate them with a semi-colon.

Dates of data collection

Enter the start and end date of data collection in yyyyy/mm format. If data sources are distinct years enter all years of data collection separated by commas.

Examples: 2017/11–2018/03; 2014; 2019; 2021.

Key findings and/or outcomes

Enter the key findings related to LTC financing.

Author's overall conclusion

Enter the authors' conclusions as it relates to LTC financing.

Reviewer notes

For peer review only

Enter any relevant notes or comments that are not captured elsewhere.

For peer review only

Preferred Reporting Items for Systematic reviews and Meta-Analyses extension for Scoping Reviews (PRISMA-ScR) Checklist

SECTION	ITEM	PRISMA-ScR CHECKLIST ITEM	REPORTED ON PAGE #
**TITLE**			
Title	1	Identify the report as a scoping review.	1
**ABSTRACT**			
Structured summary	2	Provide a structured summary that includes (as applicable): background, objectives, eligibility criteria, sources of evidence, charting methods, results, and conclusions that relate to the review questions and objectives.	1-2
**INTRODUCTION**			
Rationale	3	Describe the rationale for the review in the context of what is already known. Explain why the review questions/objectives lend themselves to a scoping review approach.	3-5
Objectives	4	Provide an explicit statement of the questions and objectives being addressed with reference to their key elements (e.g., population or participants, concepts, and context) or other relevant key elements used to conceptualize the review questions and/or objectives.	5
**METHODS**			
Protocol and registration	5	Indicate whether a review protocol exists; state if and where it can be accessed (e.g., a Web address); and if available, provide registration information, including the registration number.	Agreement of workplan provided to the contractor
Eligibility criteria	6	Specify characteristics of the sources of evidence used as eligibility criteria (e.g., years considered, language, and publication status), and provide a rationale.	6
Information sources*	7	Describe all information sources in the search (e.g., databases with dates of coverage and contact with authors to identify additional sources), as well as the date the most recent search was executed.	6
Search	8	Present the full electronic search strategy for at least 1 database, including any limits used, such that it could be repeated.	supplementary file
Selection of sources of evidence†	9	State the process for selecting sources of evidence (i.e., screening and eligibility) included in the scoping review.	6-7
Data charting process‡	10	Describe the methods of charting data from the included sources of evidence (e.g., calibrated forms or forms that have been tested by the team before their use, and whether data charting was done independently or in duplicate) and any processes for obtaining and confirming data from investigators.	7
Data items	11	List and define all variables for which data were sought and any assumptions and simplifications made.	7 and supplementary file
Critical appraisal of individual	12	If done, provide a rationale for conducting a critical appraisal of included sources of evidence;	7

For peer review only - http://bmjopen.bmj.com/site/about/guidelines.xhtml

SECTION	ITEM	PRISMA-ScR CHECKLIST ITEM	REPORTED ON PAGE #
sources of evidence§		describe the methods used and how this information was used in any data synthesis (if appropriate).	
Synthesis of results	13	Describe the methods of handling and summarizing the data that were charted.	7
**RESULTS**			
Selection of sources of evidence	14	Give numbers of sources of evidence screened, assessed for eligibility, and included in the review, with reasons for exclusions at each stage, ideally using a flow diagram.	Figure 1
Characteristics of sources of evidence	15	For each source of evidence, present characteristics for which data were charted and provide the citations.	8-9
Critical appraisal within sources of evidence	16	If done, present data on critical appraisal of included sources of evidence (see item 12).	Supplementary file, Table 1
Results of individual sources of evidence	17	For each included source of evidence, present the relevant data that were charted that relate to the review questions and objectives.	9-21
Synthesis of results	18	Summarize and/or present the charting results as they relate to the review questions and objectives.	9-21
**DISCUSSION**			
Summary of evidence	19	Summarize the main results (including an overview of concepts, themes, and types of evidence available), link to the review questions and objectives, and consider the relevance to key groups.	21-23
Limitations	20	Discuss the limitations of the scoping review process.	23
Conclusions	21	Provide a general interpretation of the results with respect to the review questions and objectives, as well as potential implications and/or next steps.	22-23
**FUNDING**			
Funding	22	Describe sources of funding for the included sources of evidence, as well as sources of funding for the scoping review. Describe the role of the funders of the scoping review.	31

JBI = Joanna Briggs Institute; PRISMA-ScR = Preferred Reporting Items for Systematic reviews and Meta-Analyses extension for Scoping Reviews.
* Where *sources of evidence* (see second footnote) are compiled from, such as bibliographic databases, social media platforms, and Web sites.
† A more inclusive/heterogeneous term used to account for the different types of evidence or data sources (e.g., quantitative and/or qualitative research, expert opinion, and policy documents) that may be eligible in a scoping review as opposed to only studies. This is not to be confused with *information sources* (see first footnote).
‡ The frameworks by Arksey and O'Malley (6) and Levac and colleagues (7) and the JBI guidance (4, 5) refer to the process of data extraction in a scoping review as data charting.
§ The process of systematically examining research evidence to assess its validity, results, and relevance before using it to inform a decision. This term is used for items 12 and 19 instead of "risk of bias" (which is more applicable to systematic reviews of interventions) to include and acknowledge the various sources of evidence that may be used in a scoping review (e.g., quantitative and/or qualitative research, expert opinion, and policy document).

From: Tricco AC, Lillie E, Zarin W, O'Brien KK, Colquhoun H, Levac D, et al. PRISMA Extension for Scoping Reviews (PRISMAScR): Checklist and Explanation. Ann Intern Med. 2018;169:467–473. doi: 10.7326/M18-0850.

For peer review only - http://bmjopen.bmj.com/site/about/guidelines.xhtml

BMJ Open

Recent Innovations in Long-Term Care Coverage and Financing: A Rapid Scoping Review

Journal:	*BMJ Open*
Manuscript ID	bmjopen-2023-077309.R1
Article Type:	Original research
Date Submitted by the Author:	02-Jan-2024

Complete List of Authors:	Macdonald, Marilyn; Dalhousie University, Aligning Health Needs and Evidence for Transformative Change (AH-NET-C): A JBI Centre of Excellence; Dalhousie University, School of Nursing<br>Weeks, Lori; Dalhousie University, Aligning Health Needs and Evidence for Transformative Change (AH-NET-C): A JBI Centre of Excellence; Dalhousie University, School of Nursing<br>Langman, Erin; Dalhousie University, Aligning Health Needs and Evidence for Transformative Change (AH-NET-C): A JBI Centre of Excellence; Dalhousie University, School of Nursing<br>Roach, Sheri; Dalhousie University, Aligning Health Needs and Evidence for Transformative Change (AH-NET-C): A JBI Centre of Excellence; Dalhousie University, School of Nursing<br>MacNeil, Morgan; Dalhousie University, . Aligning Health Needs and Evidence for Transformative Change (AH-NET-C): A JBI Centre of Excellence; Dalhousie University, School of Nursing<br>Caruso, Julie; Dalhousie University, Aligning Health Needs and Evidence for Transformative Change (AH-NET-C): A JBI Centre of Excellence; Dalhousie University, School of Nursing<br>Tricco, Andrea; Unity Health Toronto, St. Michael's Hospital, Li Ka Shing Knowledge Institute; University of Toronto, Dalla Lana School of Public Health, Epidemiology Division and Institute of Health Policy, Management, and Evaluation<br>Pham, Ba; University of Toronto, (retired)<br>Straus, Sharon; Unity Health Toronto, St. Michael's Hospital, Li Ka Shing Knowledge Institute; University of Toronto, Institute of Health Policy, Management and Evaluation<br>Mishra, Sujata; University of Toronto, Institute of Health Policy, Management and Evaluation; University of Toronto Dalla Lana School of Public Health<br>Isaranuwatchai, Wanrudee; Health Intervention and Technology Assessment Program, (HITAP); Unity Health Toronto<br>Cormack, Gordon; University of Waterloo, David R. Cheriton School of Computer Science<br>Grossman, Maura; University of Waterloo, David R. Cheriton School of Computer Science and School of Public Health Sciences; York University, Osgoode Hall Law School<br>Yakubovich, Alexa R; Dalhousie University, Department of Community Health and Epidemiology; St Michael's Hospital, Li Ka Shing Knowledge Institute<br>Mojbafan, Arezoo; Dalhousie University, Aligning Health Needs and Evidence for Transformative Change (AH-NET-C): A JBI Centre of

	Excellence; Dalhousie University, School of Nursing<br>Ignaczak, Melissa; Dalhousie University, Aligning Health Needs and Evidence for Transformative Change (AH-NET-C): A JBI Centre of Excellence; Dalhousie University, School of Nursing<br>Leid, Caron; SPOR Evidence Alliance, Patient and public partner<br>Watt, Jennifer; University of Toronto<br>Stevens, Susan; Nova Scotia Health Authority<br>Khan, Tayaba; SPOR Evidence Alliance, Patient and public partner<br>Curran, Janet; Dalhousie University, Aligning Health Needs and Evidence for Transformative Change (AH-NET-C): A JBI Centre of Excellence; Dalhousie University, School of Nursing<br>Moody, Elaine; Dalhousie University, Aligning Health Needs and Evidence for Transformative Change (AH-NET-C): A JBI Centre of Excellence; Dalhousie University, School of Nursing<br>Rodrigues, Ricardo ; Universidade de Lisboa, SOCIUS/CSG, ISEG (Lisbon School of Economics & Management)
**Primary Subject Heading**:	Geriatric medicine
Secondary Subject Heading:	Health policy
Keywords:	Aging, Health Services Accessibility, Health Services for the Aged

SCHOLARONE™
Manuscripts

I, the Submitting Author has the right to grant and does grant on behalf of all authors of the Work (as defined in the below author licence), an exclusive licence and/or a non-exclusive licence for contributions from authors who are: i) UK Crown employees; ii) where BMJ has agreed a CC-BY licence shall apply, and/or iii) in accordance with the terms applicable for US Federal Government officers or employees acting as part of their official duties; on a worldwide, perpetual, irrevocable, royalty-free basis to BMJ Publishing Group Ltd ("BMJ") its licensees and where the relevant Journal is co-owned by BMJ to the co-owners of the Journal, to publish the Work in this journal and any other BMJ products and to exploit all rights, as set out in our licence.

The Submitting Author accepts and understands that any supply made under these terms is made by BMJ to the Submitting Author unless you are acting as an employee on behalf of your employer or a postgraduate student of an affiliated institution which is paying any applicable article publishing charge ("APC") for Open Access articles. Where the Submitting Author wishes to make the Work available on an Open Access basis (and intends to pay the relevant APC), the terms of reuse of such Open Access shall be governed by a Creative Commons licence – details of these licences and which Creative Commons licence will apply to this Work are set out in our licence referred to above.

Other than as permitted in any relevant BMJ Author's Self Archiving Policies, I confirm this Work has not been accepted for publication elsewhere, is not being considered for publication elsewhere and does not duplicate material already published. I confirm all authors consent to publication of this Work and authorise the granting of this licence.

For peer review only

TITLE: Recent Innovations in Long-Term Care Coverage and Financing: A Rapid Scoping Review

AUTHORS: Marilyn Macdonald[1,2] (marilyn.macdonald@dal.ca), Lori E. Weeks[1,2] (lori.weeks@dal.ca), Erin Langman[1,2] (erin.langman@dal.ca), Sheri Roach[1,2] (sheri.roach@dal.ca), Morgan X. MacNeil[1,2] (morgan.macneil@dal.ca), Julie Caruso[1,2] (julie.caruso@dal.ca), Andrea C. Tricco[3,4,5] (andrea.tricco@unityhealth.to), Ba' Pham[6] (ba.pham@theta.utoronto.ca), Sharon Straus[3,7] (sharon.straus@utoronto.ca), Sujata Mishra[6] (sujata.mishra@mail.utoronto.ca), Wanrudee Isaranuwatchai[7,8,9] (wanrudee.i@hitap.net), Gordon V. Cormack[10] (gvcormack@uwaterloo.ca), Maura R. Grossman[10,11] (maura.grossman@uwaterloo.ca), Alexa R. Yakubovich[3,12] (alexa.yakubovich@dal.ca), Arezoo Mojbafan[1,2] (arezoo.mojbafan@dal.ca), Melissa Ignaczak[1,2] (melissa.ignaczak@dal.ca), Caron Leid[13] (caronleid@gmail.com), Jennifer Watt[6] (jennifer.watt@utoronto.ca), Susan Stevens[14] (susan.stevens@nshealth.ca), Tayaba Khan[13] (drtayabakhan@gmail.com), Janet Curran[1,2] (jacurran@dal.ca), Elaine Moody[1,2] (elaine.moody@dal.ca), Ricardo Rodrigues[15] (ricardo.rodrigues@iseg.ulisboa.pt)

CORRESPONDING AUTHOR: Marilyn Macdonald, Room 130, Forrest Bldg., Dalhousie University, PO Box 15000, 5869 University Avenue, Halifax NS B3H 4R2, 902-494-2433

1. Aligning Health Needs and Evidence for Transformative Change (AH-NET-C): A JBI Centre of Excellence, Halifax, Nova Scotia, Canada; 2. School of Nursing, Dalhousie University, Halifax, Nova Scotia, Canada; 3. Li Ka Shing Knowledge Institute, St. Michael's Hospital, Unity Health Toronto, Toronto, Canada; 4. Epidemiology Division and Institute of Health Policy, Management, and Evaluation, Dalla Lana School of Public Health, University of Toronto, Toronto, Ontario, Canada; 5. Queen's Collaboration for Health Care Quality: A JBI Centre of Excellence, Queen's University School of Nursing, Kingston, Ontario, Canada; 6. University of Toronto, Toronto, Ontario, Canada; 7. Institute of Health Policy, Management and Evaluation, University of Toronto, Toronto, Canada; 8. Unity Health Toronto, Toronto, Ontario, Canada; 9. Health Intervention and Technology Assessment Program (HITAP), Thailand; 10. David R. Cheriton School of Computer Science and School of Public Health Sciences, University of Waterloo, Waterloo, Ontario, Canada; 11. Osgoode Hall Law School, York University, Toronto, Ontario, Canada; 12. Department of Community Health and Epidemiology, Dalhousie University; 13. Patient and public partner; 14. Nova Scotia Health, Nova Scotia, Canada; 15. SOCIUS/CSG, ISEG (Lisbon School of Economics & Management), Universidade de Lisboa, Lisboa, Portugal

For peer review only

KEYWORDS: Long-Term Care; Aging; Health Care Costs; Sustainable Development

WORD COUNT: 4,782

ABSTRACT

Objectives: To identify, chart, and analyze the literature on recent initiatives to improve long-term care (LTC) coverage, financial protection and financial sustainability for persons aged 60 and older.

Design: Rapid scoping review

Data sources: Four databases and four sources of grey literature were searched for reports published between 2017 and 2022. After using a supervised machine learning tool to rank titles and abstracts, two reviewers independently screened sources against inclusion criteria.

Eligibility criteria: Studies published 2017-2022 in any language that captured recent LTC initiatives for people aged 60 and older, involved evaluation and directly addressed financing were included.

Data extraction and analysis: Data were extracted using a form designed to answer the review questions, and analyzed using descriptive qualitative content analysis, with data categorized according to a pre-specified framework to capture the outcomes of interest.

Results: Of 24 reports, 22 were published in peer-reviewed journals, and two were grey literature sources. Study designs included quasi-experimental, policy analysis or comparison, qualitative description, comparative case study, cross-sectional study, systematic literature review, economic evaluation, and survey. Studies addressed coverage based on level of disability, income, rural/urban residence, employment, and citizenship. Studies also addressed financial protection, including out-of-pocket (OOP) expenditures, co-payments, and risk of poverty related to costs of care. The reports addressed challenges to financial sustainability such

For peer review only

as lack of service coordination and system integration, insufficient economic development, and inadequate funding models.

Conclusions: Initiatives where LTC insurance is mandatory and accompanied by commensurate funding are situated to facilitate aging in place. Efforts to expand population coverage are common across the initiatives, with the potential for wider economic benefits. Initiatives that enable older people to access the services needed while avoiding OOP-induced poverty contribute to improved health and well-being. Preserving health in older people longer may alleviate downstream costs and contribute to financial sustainability.

ARTICLE SUMMARY

Strengths and Limitations of this Study

- This study uses the WHO definition of long-term care, which includes care across the continuum for older adults and does not exclude studies based on the type of care provided.
- This study identifies gaps in the existing literature and suggests areas for improvement, such as the importance of capturing equity measures.
- Due to the design of the review, long-established LTC programs and new initiatives that are yet to be evaluated may not be captured, as well as proprietary reports and government and health authority internal reports.
- Some of the included reports analyzed textual data from policy data in the absence of implementation data.

INTRODUCTION

The proportion of the global population aged 65 years and above is projected to grow from 10% in 2022 to 16% in 2050. (1) Due to rapidly aging populations, a greater prevalence of chronic conditions including dementia, gains in life expectancy, changing family dynamics and

For peer review only

living arrangements, and shortages of paid and unpaid caregivers, the demand for long-term care (LTC) services continues to expand. (2) Governments worldwide are challenged to create and adapt public programs to ensure that older adults have access to high quality LTC services. Accordingly, many governments undertook LTC system reforms.

In most countries, LTC services comprise a fraction of all healthcare expenditures. (3) The bulk of costs is covered by governments and social insurance with most people cared for in their own homes. (4) In general, systems of public LTC coverage can be clustered into two main types: 1) universal publicly funded system, and 2) a means-tested, safety-net system where public funding is available for those who cannot afford to pay for LTC. Universal LTC is available in many countries, including Japan, Germany, Luxembourg, the Netherlands, South Korea, Denmark, Finland, Norway, and Sweden. (5) Other countries, such as England and the United States, rely heavily on a means-tested, safety-net system. (5) The two main types of public LTC coverage can also be used in combination, as in the case of Canada, Austria, Australia, France, Greece, Ireland, Italy, Spain, Poland, New Zealand, and Switzerland. (5) A systematic review (6) noted that each country features its own distinct public LTC insurance (LTCI) implementation techniques and that public LTCI in all countries must continuously adapt to local conditions to function sustainably.

Researchers have noted the substantial variations in LTC funding approaches between low- and high-income regions, as well as the difficulty in finding relevant indicators and measures that can be adapted to different settings. Through a scoping review, Seyede and colleagues identified several indicators (e.g., OOP expenditures, geographic coverage, and proportion of older people receiving LTC) to measure progress towards Universal Health

For peer review only

Coverage in the context of population aging but they noted that many of these indicators would not be feasible in low- and middle-income countries. (7)

For the purposes of this review LTC includes service provision in the community, such as homecare and support, rehabilitative care, palliative care, residential care homes, assisted living facilities, nursing homes, as well as any other institutional setting. (8) LTC coverage not only includes care provided in facilities, it also includes programs such as cash benefits, community services in kind, day programs, residential facility care and home care. (8) According to WHO, "financial protection is achieved when direct payments made to obtain health services do not expose people to financial hardship and do not threaten living standards." (9 para.1) Financial protection via Universal Health Coverage is also a WHO Sustainable Development goal. (10) Financial sustainability "refers to the extent to which a given set of fiscal policies for LTC does not shift too large a financial burden on future generations (i.e., intergenerational fiscal equity) and ensures that "ends meet." (11)

The focus of this scoping review is LTC funding, specifically, the knowledge gap regarding what is known about recent LTC initiatives that directly address financing. Addressing the complex needs of the world's rapidly aging population will require a focus on improving aspects of LTC service coverage, financial protection, and financial sustainability. The primary objective of this scoping review is to identify, chart and analyze the literature on recent public initiatives (i.e., reforms, policies and programs) that have been undertaken to improve LTC coverage, financial protection and financial sustainability.

Review Question

What recent initiatives have been implemented to improve service coverage, financial protection and financial sustainability in the LTC sector for persons 60 years and older?

For peer review only - http://bmjopen.bmj.com/site/about/guidelines.xhtml

METHODS

This rapid scoping review was conducted in accordance with the JBI methodology for scoping reviews (12) and adheres to the reporting standards outlined in the Preferred Reporting Items for Systematic Reviews and Meta-Analyses extension for scoping reviews (PRISMA-ScR). (13) The WHO guide on rapid reviews (14) was consulted to help tailor the rapid review methods to decision-makers' needs. The Strategy for Patient Oriented Research – Evidence Alliance (SPOR-EA) processes for integrated knowledge translation were followed. (15-18) The WHO Centre for Health Development (WHO Kobe Centre) contracted this review. An agreement of work plan was provided to the contractor in lieu of a published protocol.

Search Strategy

After a limited search for seed articles in MEDLINE, two librarians (EL, JC) created an initial search strategy for MEDLINE that was peer-reviewed by a third librarian using Peer Review of Electronic Search Strategies (PRESS). (19) A librarian translated the search strategy for CINAHL, EMBASE and EconLit. Sources of grey literature included ClinicalTrials.gov, WHO International Clinical Trial Registry Platform (ICTRP) and ProQuest Dissertations and Theses. Canada's Drug and Health Technology Agency (CADTH) Grey Matters database was also used to identify additional sources of grey literature under the category of "health economics." All search strategies are available in the online supplemental file and are reported using Preferred Reporting Items for Systematic reviews and Meta-Analyses literature search extension (PRISMA-S). (20)

Inclusion and Exclusion Criteria

Studies published in any language, from January 2017 to August 25, 2022, that captured recent LTC initiatives for people aged 60 and older, involved evaluation and directly addressed

For peer review only

financing were included. Eligible study designs included randomized control trials (RCT), non-randomized controlled studies, before and after studies, interrupted time-series, quasi-experimental studies, qualitative studies, mixed methods, economic evaluation, systematic reviews and policy analysis reports. Reasons for exclusion of literature sources included failure to address coverage, quality, financial protection, and financial sustainability; no evaluation component, and study populations under age 60.

Study Screening and Data Extraction

Bibliographic records were identified for screening using the Continuous Active Learning® (CAL®) tool (21, 22) which uses supervised machine learning to rank titles and abstracts from most to least likely to be of interest. Results of the database searches were uploaded to Covidence (Veritas Health Innovation, Melbourne, Australia) and duplicates were removed. Three reviewers independently reviewed the first 1500 titles and abstracts. Potentially relevant full-text articles were then screened by two team members. All discrepancies were resolved by discussion or a third reviewer. The results of the search and the screening process are reported in full and presented in a Preferred Reporting Items for Systematic Reviews and Meta-Analyses (PRISMA) 2020 flow diagram. (23)

A data-extraction form was developed, and pilot-tested using a random sample of five included reports. Data were extracted on study characteristics, outcomes, and PROGRESS-Plus (24, 25) characteristics, where possible (see the online supplemental file). Reports written in languages other than English were reviewed and data extracted by team members fluent in those languages.

Quality Appraisal

For peer review only

Although quality appraisal is typically not conducted for a scoping review (14), it was conducted at the request of the commissioner. The appraisal was conducted by one team member and independently verified by a second team member. JBI methodological quality assessment tools (26-28) were selected based on the designs of included reports. Review articles were appraised using the Health Evidence™ Quality Appraisal Tool. (29) Discrepancies were resolved through team discussion.

Analysis

Using descriptive qualitative content analysis, (12) data was categorized according to the key outcomes of interest, coverage, financial protection and financial sustainability in LTC. Supplemental Table 1 outlines study characteristics, including initiatives and quality appraisal of included reports.

Patient and Public Involvement

Two public partners, co-authors, attended regular team meetings over a six-month period and provided input into the design of the study. They also reviewed and commented on report drafts, tools, and manuscript drafts, as well as dissemination activities.

RESULTS

The search retrieved 51,766 electronic database and grey literature records. Following screening at the title/abstract level, 100 reports were identified for full-text review (Figure 1). Of these, 24 reports met the inclusion criteria for this rapid scoping review.

Characteristics of Included Studies

Of the 24 reports, 22 were published in peer-reviewed journals, and two were grey literature sources. (30, 31) Just over half of the reports addressed initiatives in China (n=13). (32-44) The remainder originated in the United States (U.S., n=3) (45-47), Singapore (n=2) (48, 49),

For peer review only

Taiwan (n=2) (50, 51), and the Netherlands (n=1). (52) Three reports covered multiple countries. (30, 31, 53) All 24 reports were published between 2019 and 2022. The study designs included quasi-experimental (n=7) (32, 34, 35, 41, 43, 44, 48), policy analysis or comparison (n=6) (31, 38, 42, 49, 51, 53), qualitative research (n=3) (40, 47, 50), comparative case study (n=2) (30, 45), cross-sectional study (n=2) (33, 37), systematic literature review (n=2) (36, 39), economic evaluation (n=1) (52), and survey (n=1) (46) (see Supplemental Table 1).

Included studies analyzed secondary data (n=10) (30-33, 37, 41, 43, 44, 48, 52), policy documents (n=6) (36, 38, 39, 42, 51, 53), interviews (n=6) (33, 40, 45, 49, 50, 53) and survey data (n=3). (35, 46, 47) One study had data from both policy documents and interviews. (53)

All included sources focused on people aged 60 and above, but not all included data collected from older adults. Six reports included data from LTC stakeholders (40, 45-47, 50, 53). For example, Beauregard and Miller (45) conducted interviews with bureaucrats, consumer and provider advocacy groups, consultants and policy experts. For these six studies, we have indicated both who the data concerned and who provided the data (see Supplemental Table 1). Of the remaining 18 reports, nine (32-35, 37, 41, 43, 44, 48) reported findings from a LTC population and nine (30, 31, 36, 38, 39, 42, 49, 51, 52) focused on analyzing policy, comparing LTC systems across countries, or analyzing government spending on LTC (see Supplemental Table 1).

Quality Appraisal

Twenty-one out of 24 reports underwent critical appraisal. The three reports excluded from appraisal included two (30, 31) summarizing and interpreting existing studies and the third (42) used a design that approximated a quality appraisal. Overall studies were appraised at moderate to high in quality (see Supplemental Table 1).

For peer review only

Long-Term Care Initiatives

The LTC initiatives are described in Table 1.

Table 1: LTC Initiatives

Initiative	Country	Author Year	Description
LTCI Pilots	China	Chen et al. 2020 (33)<br>Chen & Ning 2022 (41)<br>Dai et al. 2022 (39)<br>Feng 2021 (38)<br>Han & Shen 2022 (40)<br>Lei et al. 2022 (32)<br>Liu & Hu 2022 (44)<br>Peng et al. 2022 (42)<br>Tang et al. 2022 (43)<br>Wu et al. 2020 (37)<br>Zhang & Yu 2019 (35)<br>Zhang et al. 2020 (34)<br>Zhou & Dai 2021 (36)	• Nearly universal health insurance coverage yet did not include community LTC coverage before the pilots<br>• LTCI pilots instituted to learn and plan how best to deliver LTC<br>• Began in 2016 with 16 cities and expanded to 49 cities by 2020<br>• Pilot cities explored multiple sources of financing and customized benefits packages based on their unique needs<br>• Aimed to promote formal (paid) care, while also encouraging informal care
CareShield Life	Singapore	Fong & Borowski 2022 (49)	• Legislated LTC social insurance<br>• Coverage for citizens and permanent residents born on or after January 1, 1980; entry age is 30; inclusive of people with severe disabilities and the economically disadvantaged<br>• Assessed for eligibility by the Ministry of Health (i.e., disability in performing at least three ADLs)<br>• Lifetime coverage; a claim can be made at any age<br>• Benefits increase annually by 2% for the first five years; premiums stop at age 67; the later a claim is made the higher the monthly payout
LTC 2.0 Reform	Taiwan	Chen & Fu 2020 (51)<br>Chiu et al. 2019 (50)	• Expansion of LTC to target aging in place<br>• Eligibility determined by assessing ADL, IADL, cognition, behavioural changes, rehabilitation, home situation, and caregiver stress<br>• Eligible populations: people ≥65 with ADL limitations as well as frailty; 2. people with dementia aged ≥50<br>• Changes included a reduction of co-payments (meaning the premium amount paid for services), a simplified contract system for service providers, modified care worker service fees, government subsidies for rural areas, family caregiver support centres, dementia support centres, and community care stations
Community First Choice	U.S.	Beauregard & Miller 2021 (45)	• The federal government program allowing states to expand Medicaid home and community-based services through the Affordable Care Act<br>• 6% federal increase in matching payments to shift spending from institutional to home-based care<br>• Provides individuals considered to be at an institutional level of care with ADL and IADL assistance

For peer review only - http://bmjopen.bmj.com/site/about/guidelines.xhtml

			• Agency-delivered or self-directed (clients take responsibility for hiring, supervising, and de-hiring workers)
LTC Reform	The Netherlands	Alders & Shut 2022 (52)<br>Hashiguchi & Llena-Nozal 2020 (30)<br>Kotschy & Bloom, 2022 (31)	• Reform initiated in response to high institutional care costs and focused on intensive home care as a substitute for institutional care<br>• Social care responsibility transferred to municipalities

LTCI=long-term care insurance; ADL=activities of daily living; IADL=instrumental activities of daily living

Equity

Although the term "gender" was used consistently, only biological sex (female-male) was reported. Nine studies reported gender (32-34, 37, 40, 41, 43, 44, 48) (42.3% male). Place of residence was occasionally unclear. Education level and socio-economic status, and age were reported in multiple ways based on the study design and variables included. Supplemental Table 2 reports the PROGRESS-Plus characteristics reported by study.

For peer review only

LTC Insurance Coverage

The LTCI pilots in China represented LTC in the home setting and in institutions. The countries involved in the remaining initiatives have been delivering LTC in home care and have moved to either enhance initiatives or to reform existing initiatives. Table 2 outlines the five initiatives, associated studies and a summary of coverage by initiative.

Table 2: Coverage by Country and Initiative

Initiative	Country	Author Year	Coverage	Coverage Effect
LTCI pilots	China	Dai et al. 2022 (39)<br>Feng et al. 2021 (38)<br>Han & Shen 2022 (40)<br>Lei et al. 2022 (32)<br>Peng et al. 2022 (42)<br>Zhou & Dai 2021 (36)	Eligible populations: seven pilot cities provided coverage to UEBM participants only. Eight cities expanded coverage to include: both urban and non-employees of UEBMI and URBMI (n=2); urban employees enrolled in UEBMI and rural and urban residents aged 60 and over enrolled in URBMI and NRCMS (n=1); all rural and urban participants in UEBM, UEBMI, and URBMI (n=5)	Eligibility criteria is determined by assessment of disability (assessment tools varied). People with severe disability were eligible in all pilot cities (32, 40), with some cities including coverage for moderate/mild disability (n=3) (32) and dementia (n=6) (40)

CareShield Life	Singapore	Fong & Borowski 2022 (49)	Eligible population: citizens and permanent residents born on or after 1 January 1980 who are ≥ age 30.	The overall assessment indicates benefits are modest and that depth of coverage is sacrificed to breadth.
LTC 2.0	Taiwan	Chen & Fu 2020 (51)	Eligible populations: people ≥65 with ADL limitations as well as frailty, and people with dementia aged ≥50.	Eligibility criteria determined by assessing ADL, IADL, cognition, behavioural changes, rehabilitation, home situation, and caregiver stress.<br>LTC includes health prevention and supportive community services; excludes institutional care.
Community First Choice	U.S.	Beauregard & Miller 2021 (45)	Eligible populations: Individuals who are Medicaid-eligible. States who adopt the program must provide services state-wide to everyone eligible.	Beauregard and Miller (45) found that states were concerned that this level of coverage would cause budgetary issues, and that "the added cost of CFC was not something that state officials were able or willing to shoulder."p.184
LTCI Reform	The Netherlands	Alders & Shut 2022 (52)	Eligible populations: Age 60 years and above. Social care transferred to municipalities, institutional care a federal responsibility, strict rules for eligibility.	Municipalities with low economic solvency directed those eligible to institutional care

UEBMI=Urban Employee Basic Medical Insurance; URBMI=Urban Resident Basic Medical Insurance; NRCMS=New Rural Cooperative Medical Scheme; CFC=Community First Choice; ADLs = activities of daily living; IADLs = instrumental activities of daily living

Initiative – LTC Pilots China

Six reports (32, 36, 38-40, 42) addressed coverage (Table 2) where near-universal health insurance coverage is provided through three social health schemes: the Urban Employee Basic Medical Insurance (UEBMI), the Urban Resident Basic Medical Insurance (URBMI) and the New Rural Cooperative Medical Scheme (NRCMS) for rural residents. The pilots across 15 cities in China were funded through these existing schemes and largely covered the severely disabled. (32, 36, 40) Unmet care needs related to ADL and IADL were common in the absence of LTCI. (32) Seven of the pilot cities provided coverage for urban residents only, while eight expanded to varying degrees to provide coverage to rural areas as well as the non-employed. (39) Eligible participants are covered for both institutional and home care, however, in some cities, coverage for institutional care was more comprehensive than for home care. (39, 40)

For peer review only

Initiative – CareShield Life Singapore

In 2019, Singapore implemented a mandatory social insurance scheme for citizens and permanent residents aged 30 and older born on or after January 1, 1980. Eligibility is determined by disability assessment (disability in performing at least three ADLs). CareShield Life (49) provides lifetime coverage, a claim can be made at any age, and premiums stop at age 67.

Initiative – Taiwan LTC 2.0 Plan

Taiwan's publicly funded LTC 2.0 Plan (51) is directed to aging in place, and institutional care is not included in the initiative. The program's successes included expanding coverage through the reduction of co-payments from 30% to 16% and waiving these for low-income users. The plan also includes family caregiver support centres as well as dementia support centres for rapid diagnosis and access to services. Community care stations offering healthy living programs focused on exercise and health self-management are part of the program. (51)

Community First Choice – U.S.

Medicaid-supported LTC services and supports excludes many older people with a disability, and that only 11% of older adults are Medicaid eligible (qualifiers include citizenship or permanent residency, income, household size, disability, family status and other factors). (47) Therefore, communities continuously fundraise to provide services to older people who do not qualify for Medicaid and cannot afford to purchase LTC services. (47) One recent initiative is the CFC Program (45) through which the federal government provides participating states with matching funding targeted at providing the services needed in the home setting to offset the need for institutional care. Many states are ideologically opposed to added government oversight (45) and/or may be fiscally unable to meet matching funding to maintain the program.

For peer review only

263 Initiative – Netherlands LTC Reform

264 The Netherlands has a long history of comprehensive public coverage of LTC (52), and a recent reform to address increasing costs involved shifting the financing of home care away from the central government to municipalities, while maintaining the financing of institutional care. Municipalities where the fiscal status is sound are well situated to manage the shift, while those that are not find themselves in the position of directing people with home care needs to institutional care which is still financed by the central government. This was an unintended effect of the reform.

271 **Financial protection in LTC**

272 Eleven reports (30-33, 40, 41, 43, 48, 49, 51, 52) addressed financial protection, two of which focused on OOP expenses. One (38) additional report contributed points relevant to financial protection. Table 3 outlines financial protection in relation to the five initiatives.

275 **Table 3: Financial Protection Outcomes by Country and Initiative**

Initiative	Country	Author Year	Financial protection outcomes
LTCI pilots	China	Chen & Ning 2022 (41)<br>Feng et al. 2021 (38)<br>Han & Shen 2022 (40)<br>Lei et al. 2022 (32)<br>Tang et al. 2022 (43)	• LTCI significantly reduced inpatient OOP expenditure, but not outpatient OOP expenditure. Individuals reporting fair health had higher OOP compared to those with poor health. (41)<br>• Existing medical schemes are being used to finance the pilots; shifting of funds to LTCI could drive up OOP costs for URBMI and NRCMS enrolees. (38)<br>• LTCI pilots do not financially protect rural residents, migrant workers, and employees in new forms of employment. Across pilots funding criteria vary with geography, occupation, and age. LTC institutions offer greater financial protection than home care.<br>• For each additional year of LTCI coverage, OOP medical expenses were reduced by 23.5%. (40)<br>• Implementation of LTCI decreased outpatient expenses (22.82%) and inpatient expenses (19.8%) in pilot cites compared to non-pilot cities. (43)
CareShield Life	Singapore	Fong & Borowski 2022 (49)	Includes lifetime protection against catastrophic spending associated with severe disability. Although the scheme is described as providing good basic LTC care needs protection there is an expectation that over time the depth of the protection will develop based on need.

LTC 2.0	Taiwan	Chen & Fu, 2020 (51)	Changes to the LTC system included a reduction of co-payments, reducing out-of-pocket spending for older adults.
Community First Choice	U.S.	Beauregard & Miller 2021 (45) Hashiguchi & Llena-Nozal 2020 (30)	In California, the costs of home care for older people with median income, no net wealth, and low care needs is not covered. It is also estimated that the public social protection system would not reduce the risk of income poverty for those earning a medium income with moderate care needs. (30)
LTCI reform	The Netherlands	Alders & Shut 2022 (52) Hashiguchi & Llena-Nozal 2020 (30) Kotschy & Bloom 2022 (31)	The use of asset-testing means that those without net wealth would have lower out-of-pocket costs than the median income for older people. Those with net wealth would still be able to afford out-of-pocket costs from their incomes alone. Public support only covers help with ADLs – for help with IADLs or social activities out of pocket costs can be up to 100%, depending on income and net wealth. (30)

LTCI = long term care insurance; OOP = out of pocket; URBMI = Urban Resident Basic Medical Insurance; NRCMS = New Rural Cooperative Medical Scheme; ADLs = activities of daily living; IADLs = instrumental activities of daily living

Initiative – LTCI Pilots China

Five reports directly (32, 33, 40, 41, 43) and one report indirectly (38) addressed financial protection in relation to the LTCI Pilots in China. These reports addressed (OOP) health expenditures (32, 41), the relationship between LTCI and the presence of LTC services (33); population groups without protection (40); and LTCI impact on medical services utilization (43). OOP health expenditures were statistically significantly reduced (23.5%) for inpatients but not for outpatients because OOP rates favor the institutional setting. (43) Existing medical schemes are being used to finance the pilots; shifting of funds to LTCI could drive up OOP costs for enrolees. (38)

Filial responsibility in China traditionally meant that family members fulfilled caregiving roles across the lifespan at rates bordering on 80%. (33) The initiation of LTCI is tied directly to the availability of LTC services for ADLs and IADLs in both rural and urban settings. (33) There was variability in who was considered eligible for LTCI. Groups such as migrant workers,

For peer review only - http://bmjopen.bmj.com/site/about/guidelines.xhtml

individuals working in new forms of employment and rural residents were often excluded. (40) Over time, there has been a gradual expansion to include these groups.

Cities piloting LTCI have experienced a decrease in medical services utilization due to a decrease in accessing both inpatient and outpatient services. Outpatient services use decreased by 22.8%, and inpatient services by 19.8%. (43) At the same time, non-pilot cities saw these percentages rise. These reductions in medical system utilization in the presence of LTCI hold promise for more optimal usage of the medical system, support for LTCI, and thus a strategic shifting of the costs from medical to social care.

Initiative – CareShield Life Singapore's LTCI

This initiative is an example of a universally publicly funded LTC scheme that extends to an entire population. (49) The scheme includes protection against catastrophic spending associated with severe disability and financial difficulty and nominal cash benefits with regular increases aimed at fostering consumer choice and flexibility, which can complement or substitute in-kind benefits and services. Although the scheme is described as providing good basic LTC care needs protection, there is an expectation that over time the depth of the protection will develop based on need.

Initiative – LTC 2.0 Taiwan

The reform of LTC undertaken in Taiwan (51) was targeted toward assisting the population of older people to age in place. The reform focused on the expansion of services that included but were not limited to dementia care, linking services such as adult daycare and respite services, family caregiver support centres, community health preventive care, links to discharge plans from hospitals and to home-based medical care. The reduction in insurance co-payments,

For peer review only

availability of LTC support services and the subsidies provided to rural areas demonstrate the attention paid to financial protection.

Community First Choice – U.S.

In recognition of the number of people ineligible for LTC via Medicaid in the U.S., the CFC Program (45) was conceptualized as an opportunity for individual states to consider protecting more of their older residents with social care needs from income poverty. Hashiguchi found that in California, even with social care systems in place, “the out-of-pocket costs for older people with mean net wealth would be higher than their incomes” (p. 32), and that the costs of home care for those with median income, no net wealth, and low care needs would not be covered, highlighting the risk of income poverty among older adults. (30)

Initiative – Netherlands LTC Reform

The LTC insurance system in the Netherlands guarantees no older person experiences income poverty, despite OOP costs for low care needs (30), because copayments are means-tested to reduce the economic burden for the poor. (31) Municipalities that were unable to fund home-care recipients over the long term directed them to institutional care. Although institutional care would financially protect care recipients, those choosing to remain in their home risk their financial protection. To counteract this, municipalities have been able to negotiate substantial reductions in maximum co-payments since 2019. (52)

Financial sustainability in LTC

Seven reports (34, 36, 40, 42, 45, 49, 52) directly address financial sustainability and three (38, 39, 50) peripherally. Table 4 portrays financial sustainability in relation to the five initiatives.

Table 4: Financial Sustainability by Country and Initiative

Initiative	Country	Author, Year	Financial sustainability findings
LTCI pilot	China	Dai et al. 2022 (39)<br>Feng et al. 2021 (38)<br>Han & Shen 2022 (40)<br>Peng et al. 2022 (42)<br>Zhang et al., 2020 (34)<br>Zhou & Dai 2021[38]	• Integrating LTCI into the existing medical insurance framework is favored to reduce administrative costs, address basic LTC needs and gradually grow programs as financing permits. "The financial sustainability of the LTCI programmes has been questionable. A main reason for this is that the value allowed to be extracted from medical insurance foundations is limited." (39 p.196)<br>• Diversification of funding sources such as cash benefits to support family caregivers and in-kind services may be necessary based on pilot funding in some cities. Such diversification is offered as a path toward sustainability. (39)<br>• Glinskaya et al. advocates for the gradual increase in individual contributions in tandem with increased financial support from central and local governments to attain sustainability. (38)<br>• Funding sources affect sustainability and most cities have more than one source. Primary reliance is on transfers from the medical insurance fund, other sources include individual contributions, organizational contributions, financial subsidies, and social donations. (40)<br>• The Policy Modelling Consistency index was employed to measure the interaction coupling coordination degree between LTCI policy, population aging and economic development to assess the sustainability of the LTC pilot system. Shanghai reached the excellent level, Nantong achieved good coordination and the remaining cities' coordination degree was basic. The coordination degree between LTCI policy, population aging and economic development was reported as low or basic and suggests that economic development is not where it needs to be to sustain LTC financing. Sustained economic growth in tandem with population ageing and modifiable LTCI policies are essential to the financial sustainability of LTCI. (42)<br>• A general lack of system integration was identified and believed to place financial sustainability in jeopardy. (36)<br>• LCTI reduced informal/family caregiver care (34)<br>• Satisfaction with formal care was high. (34)<br>• Continued expansion of LTCI is favoured (34)
CareShield Life	Singapore	Fong & Borowski 2022 (49)	Policy analysis reported CareShield Life represents a collectivist (everyone contributes) approach to LTC Social Insurance; financial sustainability is guarded by rigorous claims criteria and eligibility assessments; inclusion of younger age groups contributes to affordability (coverage begins at age 30); and depth in service provision is sacrificed for breadth. Financial forecasting of the scheme is recommended to address sustainability.
LTC 2.0	Taiwan	Chiu et al. 2019 (50)	Examined difficulty with implementation to LTC 2.0 and challenges to sustainability.
LTCI reform	The Netherlands	Alders & Shut 2022 (52)	LTC reform initiated to address high institutional care costs. Reform focused on intensive home care as a substitute for nursing homes/institutional care. Social care was transferred to municipalities. The unanticipated effect was a cost-shifting by municipalities to the central government, which funds Public LTCI (specifically institutional care). This economic evaluation

Initiative	Country	Author, Year	Financial sustainability findings
			study examined cost shifting with a focus on the solvency rate of municipalities. Results: Municipalities with low solvency (20%) had 3.79 more admissions to Public LTCI per 10,000 population over age 65, equivalent to 2.5% more admissions than average. Urban municipalities with low financial solvency, a higher population over age 80 living alone or with limitations, and with a lower income had a significantly higher number of admissions to the Public LTCI system. Authors suggest shifting can be addressed by risk adjusting their social support funding based on the proportion of older people by municipality and on service needs. Such approaches optimize the use of funding sources and location of care and contribute to system sustainability.
Community First Choice	U.S.	Beauregard & Miller 2021 (45)	CFC was developed as an alternative to long-term institutional care. Participating states receive a 6% additional federal match to transition from Medicaid home and community-based services benefits. This comparative case study (2 states with CFC and 1 without) examined factors that influenced adoption. Factors included leadership from the state Medicaid office, ability to meet the 6% matching, LTC advocacy, ideology related to federal government involvement.<br>A state government advocate stated, "we have heard states really express concerns about the budgetary implications of this and whether it be sustainable even with the 6% increased FMAP [Federal Medical Assistance Percentage]."p. 184

LTCI=long-term care insurance; CFC=Community First Choice

Initiative – LTC Pilots China

Several studies (36, 40, 42) indicated challenges to the sustainability of China's LTCI pilots. The challenges reported were a lack of system integration (36), reliance on transfers from the medical insurance fund (39, 40), and a need for policy planning to address population aging and aligns with economic development. (42) Feng et al. (38) advocated for the gradual increase in individual contributions in tandem with increased financial support from central and local governments to attain sustainability. In addition, Zhang et al. (34) conducted a survey of care recipients and their family caregivers in Shanghai to determine the impact of LTCI on informal care hours provided by family members. Informal/family caregiver care was reduced by 0.47 hours. Across the 407 families, a weekly average decrease of 12.36 hours of informal care was reported. Satisfaction with formal care was 72%. These results speak favorably for the LTCI pilots to alleviate the burden on carers and the continued expansion of LTCI.

For peer review only

Initiative – CareShield Life Singapore's LTCI

A policy analysis (49) of CareShield Life which is a mandatory scheme spreads risk across the age cohorts, while including a pre-funding component in which enrolees pay forward their future LTC needs, thus contributing to sustainability. It is estimated that less than 0.1% of the population aged 30-40 years have severe disabilities, therefore this insurance scheme is projected to not only meet LTC needs but also the needs of younger persons who may require services. These authors recognized the collectivist approach to this mandatory LTCI scheme, noting however that depth is sacrificed for breadth, and the need to conduct financial forecasting of the scheme to address sustainability.

Initiative – LTC 2.0 Taiwan

Chiu et al. addresses coverage peripherally in a qualitative descriptive study of the implementation of LTC 2.0. (50) They found that the mechanisms for service integration needed to be fully implemented and also recommended building trust between service delivery agencies and government, clarifying roles and responsibilities of government care managers and agency care managers, the development of an integrated information system, and the establishment of a budget tracking system.

Initiative – LTCI Reform the Netherlands

LTC reform (in 2015–2019) was initiated in response to high institutional care costs and focused on intensive home care as a substitute for nursing home/institutional care. Social care responsibility in the home was transferred to municipalities. Alders and Schut (52) conducted an economic evaluation of municipal-level data and estimated a random-effects model to explain variation across municipalities and to report the numbers of people admitted to LTC institutions annually. The examination of municipality solvency was part of the evaluation. Municipalities

with low solvency (20%) had 3.79 more admissions to Public LTCI per 10,000 population over age 65, equivalent to 2.5% more admissions than average. The authors suggest that shifting can be addressed by risk-adjusting their social support funding based on the proportion of older people by municipality and service needs. Such approaches optimize the use of funding sources and location of care and contribute to system sustainability.

Initiative – Community First Choice program in the U.S.

The CFC program offers a 6% federal increase in matching payments to shift spending from institutional to home-based care for individuals considered to be at an institutional level of care. Adoption and sustainability of the program are contingent upon federal home and community-based services policy that considers implications at the state level. Higher federal match rates and reducing the state share of Medicaid spending would create the conditions for expansion and sustainability. (46) A case study of two states with the CFC program (Maryland and Texas) and one state without (Oklahoma) (45) reported factors influencing the adoption of the CFC program included state ideology, fiscal status, advocacy for LTC, Medicaid leadership and the strength of existing home and community-based services.

DISCUSSION

We found that, apart from the CFC program, all initiatives either provided coverage to the entire population of the country or were working incrementally toward providing coverage to the entire population. Also common was the quest across initiatives to discern how to fund them. Older people with substantive care needs were the most likely to be eligible for services across all initiatives.

Although initiatives differed across the various countries, most entailed an increase in coverage. Strategies to increase coverage included expansion of eligibility criteria, reducing or

For peer review only

waiving co-payments based on income, and simplifying processes for home support service providers. These strategies support aging in place as do initiatives aimed at health promotion that assist older people in maintaining if not improving their physical and cognitive abilities.

The findings related to financial protection include strategies such as tackling OOP expenses and providing home support services to support aging at home, mandating LTC, providing caregiver support and striking a balance between meeting LTC needs and how to fund them. These strategies saw a decrease in accessing both outpatient and inpatient medical services and access to LTC across the lifespan. A strategy that may help in the short and medium term is to develop an equitable LTC recipient dose sliding scale using ADLs, IADLs, cognition, behavioural changes, rehabilitation, home situation and caregiver stress. A sliding scale approach with individual contextualized person-focused assessments represents a strategy to push toward universalism, advance defamilization and restrain marketization (55) through optimal care to client matching. A sliding-scale approach calls for LTC systems that are dynamic and that can increase or decrease supports and services based on changes in individual needs.

Financial sustainability was a goal across all initiatives. Evidence of this was found in the evaluation of the LTCI pilots that stressed the need for system integration, dedicated funding, controlling OOP costs, and projecting LTC needs in relation to population aging and economic development. Furthermore, Singapore mandated LTC and structured the scheme to enroll everyone at age 30, paying premiums until age 67 and ensuring sustainability. Lastly the Netherlands reform altered the funding plan to cost share with municipalities. In doing so, they discovered they needed to adjust the proportion of cost sharing based on the proportion of older people per municipality and service needs to address sustainability.

For peer review only - http://bmjopen.bmj.com/site/about/guidelines.xhtml

The findings of this review call forth the need to reflect on traditional approaches to LTC such as home care and institutional care. An alternate model could focus on enhanced services to support older adults to remain living in their homes and communities. A continuum that begins with remaining at home all the way to institutional care with more home-approximating options such as co-housing (56) may support optimal independence over a longer timeframe. This thinking is conceptually underpinned by the notion of providing the right support, in the right amount, at the right time to delay decline and to push down projections on the amount of care required, thereby contributing to financial sustainability. Such a continuum could contribute to positively addressing age-related inflation, which occurs when a group consumes more goods and services than it produces as they age. (57, 58) The control of the need for services combined with an increasing workforce dampens inflation and improves the economic growth required to sustain health and social initiatives. Health promotion can slow the decline in health thus delaying the need for supports and services.

STRENGTHS AND LIMITATIONS

This team has breadth and depth in review methodology and the conduct of reviews as well as experts in long-term care. The searches were extensive including four databases and multiple sources of grey literature, and the searches were peer reviewed. There were no language limits imposed on the searches to capture as many titles and abstracts as possible. This rapid scoping review was limited to identifying initiatives (programs, policies or reforms) implemented and evaluated in the past five years, in LTC for people aged 60 and above. Countries need to include mechanisms within their initiatives to capture evaluation data. In the evidence assessed, LTC initiatives were focused on launching as opposed to evaluation, and we expect to see more evaluation including quality addressed in future research. Undoubtedly there

For peer review only

441 are initiatives that do not appear in this report because they did not meet all the inclusion criteria. In addition, there is commonly a lag between initiatives, their evaluation and subsequent publication. There is always the possibility that, despite the number of database searches conducted, the keywords used in the searches may not have been used in a relevant publication. Many countries have long-established LTC programs; however, unless a major modification occurred and met the inclusion criteria for this review, it would not have been captured. Some of the included reports analyzed textual data from policy data in the absence of implementation data.

RESEARCH GAPS

We believe there are recent LTC initiatives in place that have not been evaluated or have been evaluated but not published, and that such evidence needs to be published for all countries/sub-nations to benefit from. The number of economic evaluations of LTC initiatives was limited, and such evaluations should be encouraged to inform evolution in LTC. In addition to evaluation implementation, data from the initiatives is important to know what works and where improvements are needed to contribute to sustainability.

Although it may have been the intent of some researchers to capture equity measures, this was not necessarily made explicit in their reports. Absent from the data are race, religion, and identification of any gender minority groups. Discussion relating the characteristics to study results/findings was limited. Consistent use of a conceptual framework to compare policies such as that proposed by Noda et al. (53), is suggested to inform comparative evaluation studies or single initiative evaluation.

CONCLUSION

For peer review only

The initiatives related to coverage, financial protection and financial sustainability identified in this review came from high, upper-middle, lower-middle and low-income[13] and hold hope for the growth of existing initiatives and their expansion within and beyond their respective countries. The ones pertaining to lower-middle and low-income economies in particular fill a gap in a body of literature that has focused on high-income countries. Initiatives where LTCI is mandatory and accompanied by a commensurate funding scheme are situated to facilitate older people aging in place. Efforts to expand population coverage are common across the initiatives, with the potential for wider economic benefits. In addition, initiatives that enable older people to access services while avoiding OOP-induced poverty contribute to improved health and well-being. Preserving health in older people longer may alleviate downstream costs and contribute to financial sustainability. The relevance and timeliness of LTC initiatives cannot be understated because by 2050, 80% of older people will live in low and middle-income countries. (59)

ETHICS STATEMENTS

Patient consent for publication

Not applicable.

ACKNOWLEDGEMENTS

The authors are grateful to Dalhousie University Librarian Louise Gillis, who completed the peer review of the search strategy.

FOOTNOTES

For peer review only

Contributors: Design of the review: MM, LEW, ACT, EL, BP, GVC, MRG, BP, SS, CL, TK, ARY; Data collection: MM, LEW, EL, AM, MI, BP, JC; Critical appraisal: MM, LEW, BP; Data analysis and interpretation: MM; Drafting the article: MM, EL, JC, SR, MXM; Critical revisions of the article: MM, LEW, EL, JC, ACT, BP, SS, SM, WI, GVC, MRG, ARY, AM, MI, CL, JW, SS (NSH), TK, DL, JC, EM, RR, SR, MXM. All authors approved the final version to be published.

Competing interests: LEW is the co-owner of an assisted living home.

Funding: This research was supported by the World Health Organization Centre for Health Development (WHO Kobe Centre – WKC: K22005).

Data sharing: All search strategies are available via searchRxiv (see online supplemental file). All additional data relevant to the study are included as supplementary information.

Ethics approval: Ethics approval not required in this rapid scoping review.

For peer review only

References

1. United Nations Department of Economic and Social Affairs. World population prospects 2022: Summary of results. 2022. https://www.un.org/development/desa/pd/sites/www.un.org.development.desa.pd/files/undesa_pd_2022_wpp_key-messages.pdf.

2. Costa-Font J, Courbage C, Swartz K. Financing long-term care: Ex ante, ex post or both? Health Econ 2015;24(S1):45-57. doi: 10.1002/hec.3152

3. Grignon M, Spencer BG. The funding of long-term care in Canada: What do we know, what should we know? Can J Aging 2018;37(2):110-20. doi: 10.1017/S0714980818000028

4. OECD. Spending on long-term care. 2020. https://www.oecd.org/health/health-systems/Spending-on-long-term-care-Brief-November-2020.pdf.

5. Feng Z, Glinskaya E. Aiming Higher: Advancing public social insurance for longterm care to meet the global aging challenge; Comment on "Financing long-term care: Lessons from Japan". Int J Health Policy Manag 2020;9(8):356-59. doi: 10.15171/ijhpm.2019.121

6. Chen L, Zhang L, Xu X. Review of evolution of the public long-term care insurance (LTCI) system in different countries: Influence and challenge. BMC Health Services Research 2020;20:1057. doi: 10.1186/s12913-020-05878-z

7. Hosseini Jebeli SS, Aziz R, Megumi R, et al. Measuring universal health coverage to ensure continuing care for older people: A scoping review with specific implications for the Iranian context. Eastern Mediterranean Health Journal 2021;27(8):806-17. https://apps.who.int/iris/handle/10665/353217.

8. World Health Organization. Framework for countries to achieve an integrated continuum of long-term care. 2021. https://apps.who.int/iris/handle/10665/349911.

9. World Health Organization. Financial protection. https://www.who.int/health-topics/financial-protection.

[dataset] 10. World Health Organization. SDG Target 3.8: Achieve universal health coverage, including financial risk protection, access to quality essential health-care services and access to safe, effective, quality and affordable essential medicines and vaccines for all. https://www.who.int/data/gho/data/themes/topics/indicator-groups/indicator-group-details/GHO/sdg-target-3.8-achieve-universal-health-coverage-(uhc)-including-financial-risk-protection.

11. Colombo F, Llena-Nozal A, Mercier J, et al. Help wanted? Providing and paying for long-term care. 2011. https://www.oecd-ilibrary.org/content/publication/9789264097759-en.

12. Peters M, Godfrey C, McInerney P, et al. Chapter 11: Scoping Reviews (2020 version). In: Aromataris E, Munn, Z, eds. JBI Manual for Evidence Synthesis 2020. doi: 10.46658/JBIMES-20-12

For peer review only - http://bmjopen.bmj.com/site/about/guidelines.xhtml

534 13. Tricco A, Lillie, E, Zarin, W, et al. PRISMA extension for scoping reviews (PRISMA-ScR): Checklist and explanation. Annals of Internal Medicine 2018;169(7):467-73. doi: 10.7326/m18-0850

537 14. Tricco AC, Langlois EV, Straus SE. Rapid reviews to strengthen health policy and systems: A practical guide. Geneva: World Health Organization 2017. https://apps.who.int/iris/bitstream/handle/10665/258698/9789241512763-eng.pdf.

540 15. Tricco AC, Zarin W, Clement F, et al. Introducing the Strategy for Patient Oriented Research (SPOR) Evidence Alliance: A partnership between researchers, patients and health system decision-makers to support rapid-learning and responsive health systems in Canada and beyond. Facets 2022;7:639-53. doi: 10.1139/facets-2021-0127

544 16. Lunny C, Zarin W, Chaudhry S, et al. An inclusive and diverse governance structure of the strategy for patient-oriented research (SPOR) Evidence Alliance. Facets 2022;7:435-47. doi: 10.1139/facets-2021-0129

547 17. Zarin W, Lunny C, Chaudhry S, et al. A Canadian model for providing high-quality, timely and relevant evidence to meet health system decision-maker needs: The SPOR Evidence Alliance. Facets 2022;7:420-34. doi: 10.1139/facets-2021-0132

550 18. Li LC, Hoens AM, Wilhelm L, et al. Patient engagement in the SPOR Evidence Alliance: Reflection and learnings. Facets 2022;7:126-38. doi: 10.1139/facets-2021-0133

552 19. McGowan J, Sampson M, Salzwedel DM, et al. PRESS peer review of electronic search strategies: 2015 guideline statement. Journal of Clinical Epidemiology 2016;75:40-6. doi: 10.1016/j.jclinepi.2016.01.021

555 20. Rethlefsen ML, Kirtley S, Waffenschmidt S, et al. PRISMA-S: An extension to the PRISMA statement for reporting literature searches in systematic reviews. Syst Rev 2021;10(1):39. doi: 10.1186/s13643-020-01542-z

558 21. Pham B, Rios P, Radhakrishnan A, et al. Comparative-effectiveness research of COVID-19 treatment: A rapid scoping review. BMJ Open 2022;12(6):e045115. doi: 10.1136/bmjopen-2020-045115

561 22. Rios P, Radhakrishnan A, Williams C, et al. Preventing the transmission of COVID-19 and other coronaviruses in older adults aged 60 years and above living in long-term care: A rapid review. Syst Rev 2020;9(1):218. doi: 10.1186/s13643-020-01486-4

564 23. Page MJ, McKenzie JE, Bossuyt PM, et al. The PRISMA 2020 statement: An updated guideline for reporting systematic reviews. BMJ 2021;372:n71. doi: 10.1136/bmj.n71

566 24. The Campbell and Cochrane Equity Methods Group. PROGRESS-Plus. https://methods.cochrane.org/equity/projects/evidence-equity/progress-plus.

568 25. O'Neill J, Tabish H, Welch V, et al. Applying an equity lens to interventions: Using PROGRESS ensures consideration of socially stratifying factors to illuminate inequities in

For peer review only

health. Journal of Clinical Epidemiology 2014;67(1):56-64. doi: 10.1016/j.jclinepi.2013.08.005

26. Gomersall JS, Jadotte YT, Xue Y, et al. Conducting systematic reviews of economic evaluations. Int J Evid Based Healthc 2015;13(3):170-78. doi: 10.1097/xeb.0000000000000063

27. Moola S, Munn Z, Tufanaru C, et al. Chapter 7: Systematic reviews of etiology and risk. In: Aromataris E, Munn, Z eds. JBI Manual for Evidence Synthesis 2020. https://synthesismanual.jbi.global.

28. Tufanaru C, Munn, Z, Aromataris, E, Campbell, J, Hopp, L. Chapter 3: Systematic reviews of effectiveness. In: Aromataris E, Munn, Z eds. JBI Manual for Evidence Synthesis 2020. https://synthesismanual.jbi.global.

29. National Collaborating Centre for Methods and Tools. Health evidence quality assessment tool. 2018. https://www.nccmt.ca/knowledge-repositories/search/275.

30. Hashiguchi TCO, Llena-Nozal A. The effectiveness of social protection for long-term care in old age: Is social protection reducing the risk of poverty associated with care needs? OECD Publishing 2020. https://EconPapers.repec.org/RePEc:oec:elsaad:117-en.

31. Kotschy R, Bloom D. A comparative perspective on long-term care systems. 2022; 75(3-4): 47-69. doi: 10.1111/issr.12307

32. Lei X, Bai C, Hong J, et al. Long-term care insurance and the well-being of older adults and their families: Evidence from China. Social Science & Medicine 2022;296:114745. doi: 10.1016/j.socscimed.2022.114745

33. Chen L, Zhang X, Xu X. Health insurance and long-term care services for the disabled elderly in China: Based on CHARLS data. Risk Management and Healthcare Policy 2020;13:155-62. doi: 10.2147/RMHP.S233949

34. Zhang H, Zhang X, Zhao Y, et al. Impact of formal care use on informal care from children after the launch of long-term care insurance in Shanghai, China. International Journal of Environmental Research and Public Health 2020;17(8): 2938. doi: 10.3390/ijerph17082938

35. Zhang Y, Yu X. Evaluation of long-term care insurance policy in Chinese pilot cities. International Journal of Environmental Research and Public Health 2019;16(20): 3826. doi: 10.3390/ijerph16203826

36. Zhou W, Dai W. Shifting from fragmentation to integration: A systematic analysis of long-term care insurance policies in China. International Journal of Integrated Care 2021;21(3):11. doi: 10.5334/ijic.5676

37. Wu J, Chen S, Wen H, et al. Health status, care needs, and assessment for beneficiaries with or without dementia in a public long-term care insurance pilot in Guangzhou, China. BMC Health Services Research 2020;20(1):1127. doi: 10.1186/s12913-020-05965-1

For peer review only

38. Feng Z, Zhuang X, Glinskaya E. Understanding China's long-term care insurance pilots: Key features, challenges and prospects. China: An International Journal 2021;19(3):168-87. doi: 10.1353/chn.2021.0034

39. Dai W, Li Y, Shen J. The pilot programme of long-term care insurance in China: Fragmentation and policy implications. China: An International Journal 2022;20(2):186-206. doi: 10.1353/chn.2022.0010

40. Han Y, Shen T. Long-term care insurance pilot programme in China: Policy evaluation and optimization options-taking the pilot programme in the northeast of China as an example. International Journal of Environmental Research and Public Health 2022;19(7): 4298. doi: 10.3390/ijerph19074298

41. Chen H, Ning J. The impacts of long-term care insurance on health care utilization and expenditure: Evidence from China. Health Policy and Planning 2022;37(6):717-27. doi: 10.1093/heapol/czac003

42. Peng R, Deng X, Xia Y, et al. Assessing the sustainability of long-term care insurance systems based on a policy-population-economy complex System: The case study of China. International Journal of Environmental Research and Public Health 2022;19(11):6554. doi: 10.3390/ijerph19116554

43. Tang Y, Chen T, Zhao Y, et al. The Impact of the long-term care insurance on the medical expenses and health status in China. Frontiers in Public Health 2022;10:847822. doi: 10.3389/fpubh.2022.847822

44. Liu H, Hu T. Evaluating the long-term care insurance policy from medical expenses and health security equity perspective: Evidence from China. Archives of Public Health 2022;80:3. doi: 10.1186/s13690-021-00761-7

45. Beauregard LK, Miller EA. A comparative analysis of state adoption of the Community First Choice program. Home Health Care Services Quarterly 2021;40(3):177-91. doi: 10.1080/01621424.2021.1947926

46. Koumoutzis A, Heston-Mullins J, Mayberry PS, et al. If you ask them, they will support: A national study of local initiatives developed to provide social care to older adults in the community. Journal of Applied Gerontology 2022;41(8):1860-69. doi: 10.1177/07334648221090945

47. Koumoutzis A, Stemen SE, Maharjan R, et al. Local initiatives to fund services for older americans: community recognition of the importance of social care. Journal of Applied Gerontology 2021;40(9):980-84. doi: 10.1177/0733464820944325

48. Sum G, Kadir MM, Ho SH, et al. Cost analysis of a Patient-Centred Medical Home for community-dwelling older adults with complex needs in Singapore. Annals Academy of Medicine Singapore 2022;51(9):553-66. doi: 10.47102/annals-acadmedsg.2022165

For peer review only

49. Fong JH, Borowski A. Long-term care insurance reform in Singapore. Journal of Aging & Social Policy 2022;34(1):73-90. doi: 10.1080/08959420.2021.1926867

50. Chiu T-Y, Yu H-W, Goto R, et al. From fragmentation toward integration: A preliminary study of a new long-term care policy in a fast-aging country. BMC Geriatrics 2019;19(1):159. doi: 10.1186/s12877-019-1172-5

51. Chen C-F, Fu T-H. Policies and transformation of long-term care system in Taiwan. Annals of Geriatric Medicine and Research 2020;24(3):187-94. doi: 10.4235/agmr.20.0038

52. Alders P, Schut FT. Strategic cost-shifting in long-term care: Evidence from the Netherlands. Health Policy 2022;126(1):43-48. doi: 10.1016/j.healthpol.2021.11.008

53. Noda S, Hernandez PMR, Sudo K, et al. Service delivery reforms for Asian ageing societies: A cross-country study between Japan, South Korea, China, Thailand, Indonesia, and the Philippines. International Journal of Integrated Care 2021;21(2):1. doi: 10.5334/ijic.4739

54. Kotschy R, Bloom D. A comparative perspective on long-term care systems [Working paper 29951]. National Bureau of Economic Research 2022. doi: 10.3386/w29951

55. Ranci C, Pavolini E. Not all that glitters is gold: Long-term care reforms in the last two decades in Europe. Journal of European Social Policy 2015;25(3):270-85. doi: 10.1177/0958928715588704

56. Canadian Cohousing Network. Home. https://cohousing.ca/.

57. Petersen T, López L. Demographics and foreign trade: What are the effects of an aging population on the current account balance of an economy? Bertelsmann Stiftung 2019. https://globaleurope.eu/globalization/effects-of-aging-population/.

58. Yoshino N, Kim CJ, Sirivunnabood P. Aging population and its impacts on fiscal sustainability. T20 Japan 2019. https://t20japan.org/policy-brief-aging-population-impacts-fiscal-sustainability/.

59. World Health Organization. Ageing and Health 2022. https://www.who.int/news-room/fact-sheets/detail/ageing-and-health.

For peer review only

668

669 **Figure 1: PRISMA flow diagram of article selection**

670 [1] Three reviewers initially independently screened 500 records using CAL® and a combined screening sample of
671 1970 for a total screening sample of 3470 records. With an estimate of duplicate records of 8.4% among the first
672 1500, we estimate humans screened a total of 3179 records using CAL®.

For peer review only

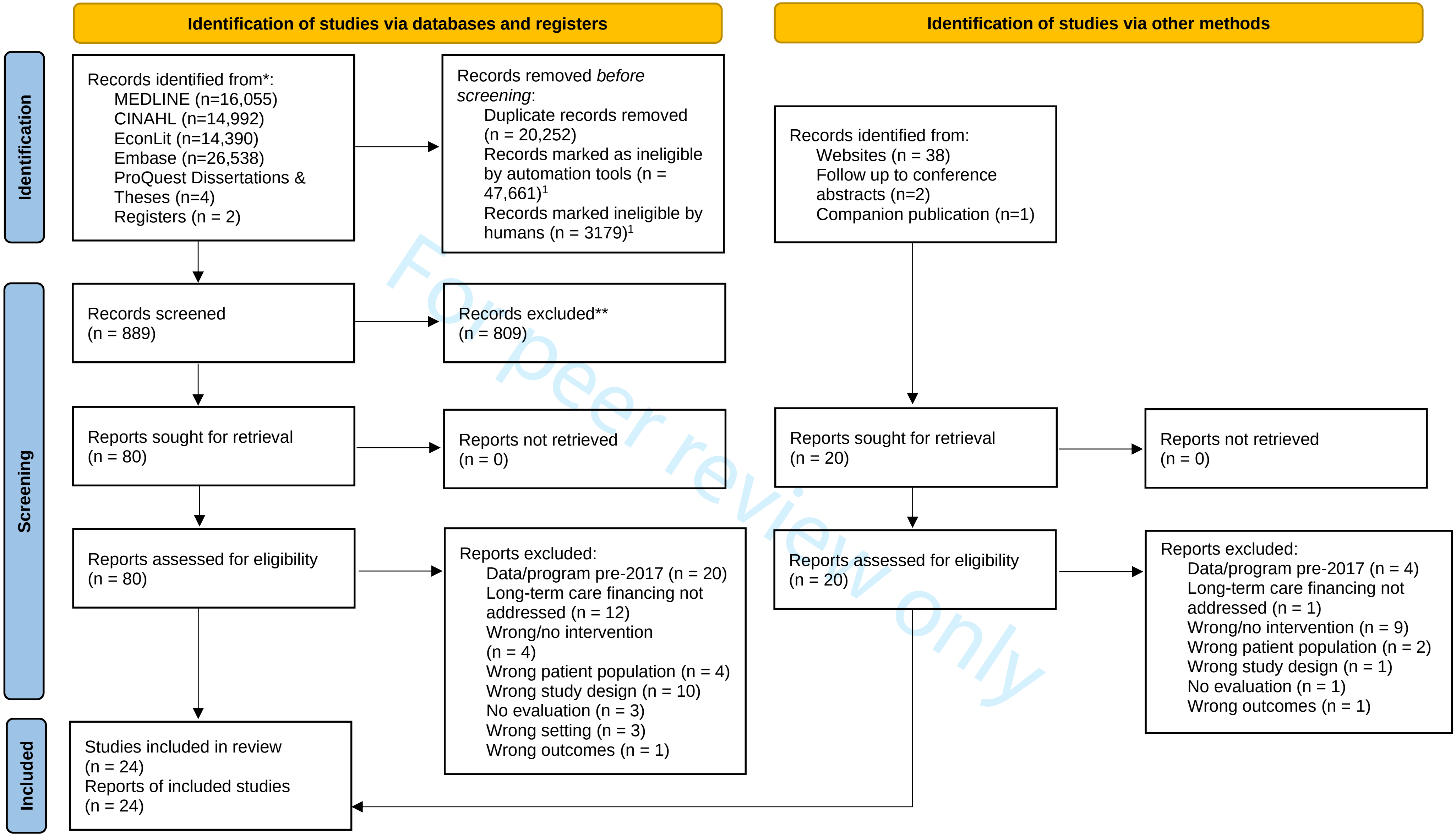

For peer review only

Table 1: Study Characteristics

Author Year	Country	Initiative	Design	Aim	Population and Data Sources	Quality Appraisal
Alders & Shut 2022(54)	The Netherlands	LTCI reform	Economic evaluation	To discern if municipalities with a low solvency rate (<20 percent) are more likely to engage in strategic cost shifting and if this resulted in an overall upward trend in admission rates to LTC services covered by public LTC services.	People aged 65+ in the Netherlands; data from 327 out of 355 municipalities from 2014–2019.<br><br>Data source(s): Monitor Long-term Care (Monitor Langdurige Zorg); Online dashboard by the Ministry of the Interior; Statistics Netherlands	Medium
Beauregard & Miller 2021(47)	U.S.	Community First Choice program	Comparative case study (qualitative)	To understand state processes related to the adoption of the Medicaid Community First Choice (CFC) Program and to identify factors that influenced states' decisions to adopt CFC.	LTC population: Medicaid-eligible older adults in Texas, Oklahoma, Maryland, and nationally (U.S.).<br><br>Study population: Data collected from n=46 individuals in roles as follows: federal and state bureaucrats, consumer and provider advocacy groups, consultants, and policy experts. Maryland (n=14), Texas (n=14), Oklahoma (n=9), and national (n=9) level participants.<br><br>Data source(s): Interviews	Medium
Chen et al. 2020(35)	China	LTCI pilots	Cross-sectional study	To evaluate the influence of health insurance and other factors on the availability of LTC services.	n=7787; Urban: n=3945; Rural: n=3842; CHARLS (2015) database adults aged 45 and up; the sample is aged 60 and up; CHARLS (2015) covered more than 10,000 families and 17,500 individuals from 150 districts of 28 provinces.<br><br>Data source(s): China Health and Retirement Longitudinal Study (CHARLS) database, 2015	Medium
Chen & Fu 2020(53)	Taiwan	LTC 2.0 reform	Policy analysis	To review the development of LTC policies, present strategies for expanding LTC, and outline policy suggestions and implications.	Older adults in Taiwan. The proportion of the population aged 65 years and over in Taiwan has doubled from 7% in 1993 to 14% in 2018. The number of older adults receiving LTC services increased from 9,148 in 2008 to	High

For peer review only

Author Year	Country	Initiative	Design	Aim	Population and Data Sources	Quality Appraisal
					258,351 in 2019, which only accounts for 41.47% of the population needing LTC.<br><br>Data source(s): Policy documents; government data	
Chen & Ning 2022(43)	China	LTCI pilot	Quasi-experimental design	To examine the policy treatment effect of long-term care insurance (LTCI) on health care utilization and OOP health expenditure (OOP) in China.	N=20,814<br>Participants in the China Health and Retirement Longitudinal Study (CHARLS).<br><br>Data source(s): China Health and Retirement Longitudinal Study (CHARLS) longitudinal study, 2018	High
Chiu et al. 2019(52)	Taiwan	LTC 2.0 reform	Qualitative research	To examine the experiences and challenges of integration for LTC plan 2.0 agencies in Taiwan	LTC population: Older adults in 20 counties where LTC Plan 2.0 was being piloted in Taiwan in early 2017.<br><br>Study population: Interviews with 6 people working in 3 LTC Plan 2.0 Tier A agencies in north, west, and central Taiwan, including a chief executive officer (CEO) or executive director (ED) and a case manager at each agency.<br><br>Data source(s): Interviews	Medium
Dai et al. 2022(41)	China	LTCI pilot	Systematic literature review of LTC policies and implementation	This study examines the LTCI pilot in China and compares it to other LTCI programs in Germany, Japan, and South Korea through a systematic literature review and discusses the implications of its implementation in a national framework.	Adults in one of the 49 expanded pilot cities in China.<br><br>Data source(s): National and local government websites; China National Knowledge Infrastructure, Baidu Scholar and international databases	High
Feng et al. 2021(40)	China	LTCI pilot	Policy analysis	To provide a timely review and assessment of China's ongoing LTCI programs	People over 60 who meet the eligibility requirement for the LTCI pilot and who live in one of the 15 pilot cities.	High

For peer review only

Author Year	Country	Initiative	Design	Aim	Population and Data Sources	Quality Appraisal
				across the initial 15 pilot cities. The focus is on synthesizing key LTCI program features across all pilot sites, not on profiling pilot sites, not on profiling and analyzing individual pilots in depth.	Data source(s): Policy documents; published research articles; grey literature; LTCI Policy Database, School of Public Health, Peking University	
Fong & Borowski 2022(51)	Singapore	CareShield Life	Policy analysis	To analyze the 2019 long-term care social insurance program, including successes and challenges, goals, design, features, financing arrangements, and initial assessment.	Older adults in Singapore. The proportion of those aged 65 and older increased x3 from 4.9% in 1980 to 14.5% in 2019.<br><br>Data source(s): Not reported. The reference list includes government documents and websites.	High
Han & Shen 2022(42)	China	LTCI pilot	Qualitative research	To explore the common problems in policy practices and to provide recommendations for further expansion of the LTCI pilot.	LTC population: China's adult population ≥60, which accounts for 18.7% of the total population.<br><br>Study population: n=10 participants, including managers (n=4), caregivers (n=2), a medical staff member (n=1), and older adults with disabilities (n=3)<br><br>Data source(s): In-depth interviews	High
Hashiguchi & Llena-Nozal 2020(32)	25 out of 41 OECD countries and EU Member States*	Public social protection systems for LTC	Comparative case study	To provide a novel set of comprehensive and internationally comparable estimates of the adequacy, equity and efficiency of public social protection systems for LTC in old age in OECD countries and EU Member States.	People 65 and older in the selected 25 OECD countries and EU Member States*.<br><br>Data source(s): Survey of Health, Ageing and Retirement in Europe (SHARE) Wave 7	N/A

For peer review only

Author Year	Country	Initiative	Design	Aim	Population and Data Sources	Quality Appraisal
Kotschy & Bloom 2022(33)	Germany, Israel, Japan, the Netherlands, South Korea	LTC systems comparison	Policy analysis	To investigate the challenges that population aging poses to long-term care.	Adults aged 65+ in Germany, Israel, Japan, the Netherlands, and South Korea<br><br>Data source(s): Survey of Health, Ageing and Retirement in Europe (SHARE) wave 7 and 8; Health and Retirement Study (HRS) wave 13; English Longitudinal Study of Ageing (ELSA) wave 8	N/A
Koumoutzis et al. 2021(49)	U.S.	Local initiatives to fund social care services	Qualitative research	To examine how communities across the nation are utilizing local funding streams to support aging services.	LTC population: Adults 60+ in the U.S.<br><br>Study population: NR<br><br>Data source(s): 2019 Advancing States national survey of State Units on Aging; telephone calls (State Units on Aging, Area Agencies on Aging, secretary of state's offices, county clerk's offices, and other officials involved in local funding efforts); web searches	Medium
Koumoutzis et al. 2022(48)	U.S.	Community LTC initiatives to support ageing	Survey	To study community initiatives developed to provide services to older adults through locally generated tax revenues and provide insight into their caregiver support efforts.	LTC population: Older adults in the U.S.<br><br>Study population: Representatives from 228 organizations across 15 states, delivering LTC services.<br><br>Data source(s): Phase 1: 2019 Advancing States national survey of state units on aging; telephone calls (telephone calls to state units on aging, area agencies on aging, aging services providers, county auditor offices, and Secretary of State offices); online reviews and web searches. Phase 2: Qualtrics survey to local funding organizations	High
Lei et al. 2022(34)	China	LTCI pilot	Quasi-experimental design	To evaluate the impact of LTCI on the covered populations in the LTCI pilots across different cities from 2015 to 2017, and to study	Older adults (aged 65+) from 152 cities: n=3,423<br>Treatment group (coverage): n=187<br>Control group (no coverage): n=3,236.	High

For peer review only

Author Year	Country	Initiative	Design	Aim	Population and Data Sources	Quality Appraisal
				various outcomes (including older adults' ADL-related need for care, family burden, medical expenditures, and some important health outcomes) among covered older adults and their families.	Data source(s): Chinese Longitudinal Healthy Longevity Survey (CLHLS)	
Liu & Hu 2022(46)	China	LTCI pilot	Quasi-experimental design	To investigate the impact of the policy pilot on the income fairness of the group system and to comprehensively analyze the institutional effect of the LTCI policy pilot.	People aged 60 and over living in one of the 15 LTCI pilot areas (n=25,063) included in CHARLS.<br><br>Data source(s): China Health and Retirement Longitudinal Study (CHARLS) database, 2013, 2015, 2018 (valid samples with a 3-year period)	High
Noda et al. 2021(55)	Japan, Korea, Thailand, China, Indonesia, the Philippines	Healthcare service delivery reform policies for the ageing	Policy comparison	To compare healthcare service delivery reform policies for older adults in Japan, Korea, Thailand, China, Indonesia and the Philippines.	LTC population: Older adults in Japan, Korea, Thailand, China, Indonesia, and the Philippines.<br><br>Study population: Health officials of provincial health and welfare departments, and service providers such as doctors and LTC providers (no n provided).<br><br>Data source(s): Document reviews; interviews with key informants	Medium
Peng et al. 2022(44)	China	LTCI pilot	Policy analysis	To assess the sustainability of the LTCI system in China using a policy population economics (PPE) system.	Population of 14 pilot cities in China. Due to a lack of data, one pilot city, Shihezi, was excluded.<br><br>Data source(s): Policy documents; China Statistical Yearbook 2020; the China City Statistical Yearbook; the Chinese National Economy and Social Development Statistics Bulletin	N/A

For peer review only

Author Year	Country	Initiative	Design	Aim	Population and Data Sources	Quality Appraisal
Sum et al. 2022(50)	Singapore	Integrated Patient-Centred Medical Home (PCMH) model	Quasi-experimental design	To address gaps in the literature on the evaluation of costs associated with the implementation of an integrated PCMH model for community-dwelling older adults with complex needs.	Patients with high biopsychosocial health risk, aged ≥40, who resided in Whampoa (n=165)<br><br>Data source(s): Client Service Receipt Inventory (CSRI) survey	High
Tang et al. 2022(45)	China	LTCI pilot	Quasi-experimental design	To evaluate the implementation effect of the Long-Term Care Insurance (LTCI) policy, and its impact on the medical expenses and the health status of the middle-aged and elder population.	Families with people middle-aged (≥45) and older in any of the 12 cities included in the CHARLS database (n=42,591)<br><br>Data source(s): China Health and Retirement Longitudinal Study (CHARLS)	Medium
Wu et al. 2020(39)	China	LTCI pilot	Retrospective cross-sectional study	To evaluate the LTCI pilot by exploring the characteristics and care needs of claimants, and performance of the assessment tool.	Claimants: n=4810; Individuals who received benefits: n=4582;<br>Aged ≥60 years: n=4357;<br>Beneficiaries without dementia: n=3791;<br>Beneficiaries with dementia: n=791;<br>Unsuccessful claimants without dementia: n=206;<br>Unsuccessful claimants with dementia: n=22<br><br>Data source(s): Claims data	Medium
Zhang & Yu 2019(37)	China	LTCI pilot	Quasi-experimental design	To explore the outcomes and evaluate the performance of the LTCI policy in the Chinese pilot cities and estimate the willingness of Chinese citizens to expand the formal implementation of LTCI policy in China.	Older adults engaged in the implementation process of the LTCI pilots in 15 pilot cities (n=1,167)<br>Elite politicians (n=5)<br><br>Data source(s): Survey questionnaire; interviews	Medium

For peer review only

Author Year	Country	Initiative	Design	Aim	Population and Data Sources	Quality Appraisal
Zhang et al. 2020(36)	China	LTCI pilot	Quasi-experimental design	To examine how the use of formal care impacts the use of informal care in Shanghai, which was one of China's first long-term insurance pilots in 2016.	Families in Shanghai that include an older adult (≥60) who had used formal care provided by LTCI for 1 to 3 months and a child who is primarily responsible for daily informal care.<br>Care recipients: n=407<br>Caregivers n=407<br><br>Data source(s): Interview-based surveys	Medium
Zhou & Dai 2021(38)	China	LTCI pilot	Systematic literature review of LTC policies	To review and assess the performance and effectiveness of the LTCI policy regime in China.	Older adults in one of the 15 pilot cities.<br><br>Data source(s): Policy documents	Medium

*The countries and subnational areas covered include Austria (Vienna region), Belgium (Flanders), Canada (Ontario), Croatia, Czech Republic, England, Estonia (Tallinn), Finland, France, Germany, Hungary, Iceland (Reykjavik), Ireland, Italy (South Tyrol), Japan, Korea, Latvia, Lithuania, Luxembourg, Slovak Republic, Slovenia, Spain, Sweden, the Netherlands, the United States of America (Illinois and California).
Quality appraisal (score % as per # of tool items): high quality=80-100%; medium quality=50-79%; low quality= <50%

For peer review only

Table 2: PROGRESS-Plus characteristics reported by study

Author	P	G/S	E	SE	SC	D	A	Author	P	G/S	E	SE	SC	D	A
Alders 2022 (52)	✓			✓		✓	✓	Koumoutzis 2021 (47)							
Beauregard 2021 (45)								Koumoutzis 2022 (46)							
Chen 2020 (33)	✓	✓	✓	✓	✓	✓	✓	Lei 2022 (32)	✓	✓		✓	✓		✓
Chen 2020 (51)	✓						✓	Liu 2022 (44)	✓	✓	✓	✓	✓	✓	✓
Chen 2022 (41)	✓	✓	✓	✓	✓	✓	✓	Noda 2021 (53)							
Chiu 2019 (50)								Peng 2022 (42)	✓					✓	✓
Dai 2022 (39)	✓						✓	Sum 2022 (48)	✓	✓	✓		✓	✓	✓
Feng 2021 (38)	✓						✓	Tang 2022 (43)		✓	✓	✓	✓	✓	✓
Fong 2022 (49)						✓	✓	Wu 2020 (37)	✓	✓				✓	✓
Han 2022 (40)	✓	✓				✓	✓	Zhang 2019 (35)	✓	✓		✓	✓	✓	✓
Hashiguchi 2020 (30)	✓			✓			✓	Zhang 2020 (34)	✓	✓	✓	✓	✓		✓
Kotschy 2022 (54)	✓					✓		Zhou 2021 (36)							

P=Place of residence; G=Gender/Sex; E=Education; SE=Socio-economic status; SC=Social capital; D=Disability; A=Age

For peer review only

Search Strategies

Search strategies are available via searchRxiv for the following information sources:

- MEDLINE: https://doi.org/10.1079/searchRxiv.2023.00214
- CINAHL: https://doi.org/10.1079/searchRxiv.2023.00213
- EconLit: https://doi.org/10.1079/searchRxiv.2023.00216
- Embase: https://doi.org/10.1079/searchRxiv.2023.00217
- ProQuest Dissertations and Theses: https://doi.org/10.1079/searchRxiv.2023.00220
- WHO ICTRP: https://doi.org/10.1079/searchRxiv.2023.00219
- ClinicalTrials.gov: https://doi.org/10.1079/searchRxiv.2023.00218

CADTH Grey Matters

All websites included in the Health Economics category of CADTH Grey Matters were searched or browsed for relevant records:

- Centre for Health Economics and Policy Analysis (McMaster University)
- Centre for Reviews and Dissemination (University of York)
- Economic Burden of Illness in Canada (Public Health Agency of Canada)
- Health Economics Research Unit (University of Aberdeen)
- IDEAS/RePEc database
- IHE publications (Institute of Health Economics, Canada)
- National Quality Measures Clearinghouse (Agency for Healthcare Research and Quality, U.S.)
- OHE publications (Office of Health Economics, United Kingdom)
- Ontario Case Costing Initiative (Ontario Ministry of Health and Long-Term Care)
- THETA Publications and Knowledge Translation to Policy (KT) Activities (Toronto Health Economics and Technology Assessment Collaborative)

If the website had a search feature, the search was as follows: ("long-term care" OR "long term care")

If there were too many results, it was narrowed by adding the following: AND (financing OR funding)

Results: 34 (38 minus 4 duplicates)

For peer review only

Data extraction form

General information

Study ID
Enter the Covidence record number here.

Example: #34

Title
Title of paper/abstract/report

Last name of lead author

Characteristics of study

Publication type
If publication type is "other" please specify the details below.

☐Journal article

☐Dissertation/thesis

☐Report

☐Other Click or tap here to enter text.

Aim/purpose/objective of study
Enter the research question or hypothesis.

Study design
Studies might not always explicitly state the design, in which case select the best option based on the methods section. If unclear, please include any relevant information in the notes section below.

☐Randomized controlled trial

☐Non-randomized experimental study

☐Quasi-experimental design

For peer review only

☐Cohort study
☐Cross-sectional study
☐Case–control study
☐Case series
☐Case report
☐Economic evaluation
☐Comparative case study
☐Policy analysis
☐Qualitative research (specific approach)
☐Other Click or tap here to enter text.

Country/setting
If the study is comparing countries/settings, ensure all the countries are listed, separated by semicolons. If the study does not mention the country but mentions a region, enter the region.

Population description
Describe the overall population. For more than one population start a new paragraph.

Gender
Enter the breakdown of population gender if reported.

Sexual orientation
Enter the breakdown of population sexuality if reported.

Race
Enter the breakdown of population racial composition if reported.

Age
Enter the overall age of population if reported. If age is reported as a range (e.g. 35–50 years) enter the relevant range and accompanying value with a colon in between (e.g. 60–79: 50%). If there are multiple ranges, separate using a semicolon (e.g. 60–79: 50%; 80–99: 50%).

Place of residence

For peer review only

Enter the breakdown of places of residence (e.g. rural, urban, suburban, long-term care [LTC] congregate setting, etc.) if reported.

Occupation
Enter the breakdown of occupations if reported.

Religious backgrounds
Enter the breakdown of religious backgrounds if reported.

Education
Enter the breakdown of educational backgrounds if reported.

Socioeconomic status
Enter the breakdown of socioeconomic status if reported.

Social capital
Social capital refers to social relationships, networks and social support. Enter information related to the social capital of the population if reported.

Disability
Enter the breakdown of the population disability categories if reported.

LTC financing
LTC system description
Describe the overall LTC system. For more than one system start a new paragraph.

Financing description
Describe how the LTC system is financed. For more than one system start a new paragraph.

Funding mechanism
Enter the details of the funding mechanism(s) in the relevant column. Enter NA in boxes where the funding mechanism is not applicable. Include the percentage mix for each funding mechanism if reported. If not reported, enter NR.

Funding mechanism	Details	Percentage mix
Guarantees		
Insurance		

For peer review only

Fees for service		
Loans		
Repayable contributions		
Result-based financing		
Equity		
Bonds		
Charitable donations		
Local government subsidies		
Taxes		
Co-payment by users		
Central government subsidies		
Other		

Service description
Describe the services covered under the programme policy reform or initiative if reported.

Coverage
How does the initiative/programme/policy/reform address LTC coverage? Enter NA if not addressed.

Quality
How does the initiative/programme/policy/reform address LTC quality? Enter NA if not addressed.

Financial protection
How does the initiative/programme/policy/reform address LTC financial protection? Enter NA if not addressed.

Financial sustainability
How does the initiative/program/policy/reform address LTC financial sustainability? Enter NA if not addressed.

Empirical evaluation
Intervention
What is the initiative/programme/policy/reform?

Comparator

For peer review only

What is the initiative/programme/policy/reform being compared to? If there is no comparator enter NA.

Intervention time frame
What is the time frame of the intervention?
Examples: policy put in place in June of 2017; pilot project from January 2017 to August 2019.

Data sources
Enter the sources of data. If there are multiple sources separate them with a semi-colon.

Dates of data collection
Enter the start and end date of data collection in yyyyy/mm format. If data sources are distinct years enter all years of data collection separated by commas.
Examples: 2017/11–2018/03; 2014; 2019; 2021.

Key findings and/or outcomes
Enter the key findings related to LTC financing.

Author's overall conclusion
Enter the authors' conclusions as it relates to LTC financing.

Reviewer notes
Enter any relevant notes or comments that are not captured elsewhere.

For peer review only

Preferred Reporting Items for Systematic reviews and Meta-Analyses extension for Scoping Reviews (PRISMA-ScR) Checklist

SECTION	ITEM	PRISMA-ScR CHECKLIST ITEM	REPORTED ON PAGE #
**TITLE**			
Title	1	Identify the report as a scoping review.	1
**ABSTRACT**			
Structured summary	2	Provide a structured summary that includes (as applicable): background, objectives, eligibility criteria, sources of evidence, charting methods, results, and conclusions that relate to the review questions and objectives.	2-3
**INTRODUCTION**			
Rationale	3	Describe the rationale for the review in the context of what is already known. Explain why the review questions/objectives lend themselves to a scoping review approach.	4-6
Objectives	4	Provide an explicit statement of the questions and objectives being addressed with reference to their key elements (e.g., population or participants, concepts, and context) or other relevant key elements used to conceptualize the review questions and/or objectives.	6
**METHODS**			
Protocol and registration	5	Indicate whether a review protocol exists; state if and where it can be accessed (e.g., a Web address); and if available, provide registration information, including the registration number.	Agreement of workplan provided to the contractor
Eligibility criteria	6	Specify characteristics of the sources of evidence used as eligibility criteria (e.g., years considered, language, and publication status), and provide a rationale.	7
Information sources*	7	Describe all information sources in the search (e.g., databases with dates of coverage and contact with authors to identify additional sources), as well as the date the most recent search was executed.	6
Search	8	Present the full electronic search strategy for at least 1 database, including any limits used, such that it could be repeated.	supplementary file
Selection of sources of evidence†	9	State the process for selecting sources of evidence (i.e., screening and eligibility) included in the scoping review.	7
Data charting process‡	10	Describe the methods of charting data from the included sources of evidence (e.g., calibrated forms or forms that have been tested by the team before their use, and whether data charting was done independently or in duplicate) and any processes for obtaining and confirming data from investigators.	7-8
Data items	11	List and define all variables for which data were sought and any assumptions and simplifications made.	7 and supplementary file
Critical appraisal of individual	12	If done, provide a rationale for conducting a critical appraisal of included sources of evidence;	8

For peer review only - http://bmjopen.bmj.com/site/about/guidelines.xhtml

SECTION	ITEM	PRISMA-ScR CHECKLIST ITEM	REPORTED ON PAGE #
sources of evidence§		describe the methods used and how this information was used in any data synthesis (if appropriate).	
Synthesis of results	13	Describe the methods of handling and summarizing the data that were charted.	8
**RESULTS**			
Selection of sources of evidence	14	Give numbers of sources of evidence screened, assessed for eligibility, and included in the review, with reasons for exclusions at each stage, ideally using a flow diagram.	Figure 1
Characteristics of sources of evidence	15	For each source of evidence, present characteristics for which data were charted and provide the citations.	8-9
Critical appraisal within sources of evidence	16	If done, present data on critical appraisal of included sources of evidence (see item 12).	Supplementary file, Table 1
Results of individual sources of evidence	17	For each included source of evidence, present the relevant data that were charted that relate to the review questions and objectives.	9-22
Synthesis of results	18	Summarize and/or present the charting results as they relate to the review questions and objectives.	9-22
**DISCUSSION**			
Summary of evidence	19	Summarize the main results (including an overview of concepts, themes, and types of evidence available), link to the review questions and objectives, and consider the relevance to key groups.	22-24
Limitations	20	Discuss the limitations of the scoping review process.	24
Conclusions	21	Provide a general interpretation of the results with respect to the review questions and objectives, as well as potential implications and/or next steps.	23-24
**FUNDING**			
Funding	22	Describe sources of funding for the included sources of evidence, as well as sources of funding for the scoping review. Describe the role of the funders of the scoping review.	32

JBI = Joanna Briggs Institute; PRISMA-ScR = Preferred Reporting Items for Systematic reviews and Meta-Analyses extension for Scoping Reviews.
* Where *sources of evidence* (see second footnote) are compiled from, such as bibliographic databases, social media platforms, and Web sites.
† A more inclusive/heterogeneous term used to account for the different types of evidence or data sources (e.g., quantitative and/or qualitative research, expert opinion, and policy documents) that may be eligible in a scoping review as opposed to only studies. This is not to be confused with *information sources* (see first footnote).
‡ The frameworks by Arksey and O'Malley (6) and Levac and colleagues (7) and the JBI guidance (4, 5) refer to the process of data extraction in a scoping review as data charting.
§ The process of systematically examining research evidence to assess its validity, results, and relevance before using it to inform a decision. This term is used for items 12 and 19 instead of "risk of bias" (which is more applicable to systematic reviews of interventions) to include and acknowledge the various sources of evidence that may be used in a scoping review (e.g., quantitative and/or qualitative research, expert opinion, and policy document).

From: Tricco AC, Lillie E, Zarin W, O'Brien KK, Colquhoun H, Levac D, et al. PRISMA Extension for Scoping Reviews (PRISMAScR): Checklist and Explanation. Ann Intern Med. 2018;169:467–473. doi: 10.7326/M18-0850.

For peer review only - http://bmjopen.bmj.com/site/about/guidelines.xhtml

BMJ Open

Recent Innovations in Long-Term Care Coverage and Financing: A Rapid Scoping Review

Journal:	*BMJ Open*
Manuscript ID	bmjopen-2023-077309.R2
Article Type:	Original research
Date Submitted by the Author:	25-Jan-2024

Complete List of Authors:	Macdonald, Marilyn; Dalhousie University, Aligning Health Needs and Evidence for Transformative Change (AH-NET-C): A JBI Centre of Excellence; Dalhousie University, School of Nursing<br>Weeks, Lori; Dalhousie University, Aligning Health Needs and Evidence for Transformative Change (AH-NET-C): A JBI Centre of Excellence; Dalhousie University, School of Nursing<br>Langman, Erin; Dalhousie University, Aligning Health Needs and Evidence for Transformative Change (AH-NET-C): A JBI Centre of Excellence; Dalhousie University, School of Nursing<br>Roach, Sheri; Dalhousie University, Aligning Health Needs and Evidence for Transformative Change (AH-NET-C): A JBI Centre of Excellence; Dalhousie University, School of Nursing<br>MacNeil, Morgan; Dalhousie University, . Aligning Health Needs and Evidence for Transformative Change (AH-NET-C): A JBI Centre of Excellence; Dalhousie University, School of Nursing<br>Caruso, Julie; Dalhousie University, Aligning Health Needs and Evidence for Transformative Change (AH-NET-C): A JBI Centre of Excellence; Dalhousie University, School of Nursing<br>Tricco, Andrea; Unity Health Toronto, St. Michael's Hospital, Li Ka Shing Knowledge Institute; University of Toronto, Dalla Lana School of Public Health, Epidemiology Division and Institute of Health Policy, Management, and Evaluation<br>Pham, Ba; University of Toronto, (retired)<br>Straus, Sharon; Unity Health Toronto, St. Michael's Hospital, Li Ka Shing Knowledge Institute; University of Toronto, Institute of Health Policy, Management and Evaluation<br>Mishra, Sujata; University of Toronto, Institute of Health Policy, Management and Evaluation; University of Toronto Dalla Lana School of Public Health<br>Isaranuwatchai, Wanrudee; Health Intervention and Technology Assessment Program, (HITAP); Unity Health Toronto<br>Cormack, Gordon; University of Waterloo, David R. Cheriton School of Computer Science<br>Grossman, Maura; University of Waterloo, David R. Cheriton School of Computer Science and School of Public Health Sciences; York University, Osgoode Hall Law School<br>Yakubovich, Alexa R; Dalhousie University, Department of Community Health and Epidemiology; St Michael's Hospital, Li Ka Shing Knowledge Institute<br>Mojbafan, Arezoo; Dalhousie University, Aligning Health Needs and Evidence for Transformative Change (AH-NET-C): A JBI Centre of

Excellence; Dalhousie University, School of Nursing
Ignaczak, Melissa; Dalhousie University, Aligning Health Needs and Evidence for Transformative Change (AH-NET-C): A JBI Centre of Excellence; Dalhousie University, School of Nursing
Leid, Caron; SPOR Evidence Alliance, Patient and public partner
Watt, Jennifer; University of Toronto
Stevens, Susan; Nova Scotia Health Authority
Khan, Tayaba; SPOR Evidence Alliance, Patient and public partner
Curran, Janet; Dalhousie University, Aligning Health Needs and Evidence for Transformative Change (AH-NET-C): A JBI Centre of Excellence; Dalhousie University, School of Nursing
Moody, Elaine; Dalhousie University, Aligning Health Needs and Evidence for Transformative Change (AH-NET-C): A JBI Centre of Excellence; Dalhousie University, School of Nursing
Rodrigues, Ricardo ; Universidade de Lisboa, SOCIUS/CSG, ISEG (Lisbon School of Economics & Management)

**Primary Subject Heading**:	Geriatric medicine
Secondary Subject Heading:	Health policy
Keywords:	Aging, Health Services Accessibility, Health Services for the Aged

SCHOLARONE™ Manuscripts

I, the Submitting Author has the right to grant and does grant on behalf of all authors of the Work (as defined in the below author licence), an exclusive licence and/or a non-exclusive licence for contributions from authors who are: i) UK Crown employees; ii) where BMJ has agreed a CC-BY licence shall apply, and/or iii) in accordance with the terms applicable for US Federal Government officers or employees acting as part of their official duties; on a worldwide, perpetual, irrevocable, royalty-free basis to BMJ Publishing Group Ltd ("BMJ") its licensees and where the relevant Journal is co-owned by BMJ to the co-owners of the Journal, to publish the Work in this journal and any other BMJ products and to exploit all rights, as set out in our licence.

The Submitting Author accepts and understands that any supply made under these terms is made by BMJ to the Submitting Author unless you are acting as an employee on behalf of your employer or a postgraduate student of an affiliated institution which is paying any applicable article publishing charge ("APC") for Open Access articles. Where the Submitting Author wishes to make the Work available on an Open Access basis (and intends to pay the relevant APC), the terms of reuse of such Open Access shall be governed by a Creative Commons licence – details of these licences and which Creative Commons licence will apply to this Work are set out in our licence referred to above.

Other than as permitted in any relevant BMJ Author's Self Archiving Policies, I confirm this Work has not been accepted for publication elsewhere, is not being considered for publication elsewhere and does not duplicate material already published. I confirm all authors consent to publication of this Work and authorise the granting of this licence.

For peer review only

TITLE: Recent Innovations in Long-Term Care Coverage and Financing: A Rapid Scoping Review

AUTHORS: Marilyn Macdonald[1,2] (marilyn.macdonald@dal.ca), Lori E. Weeks[1,2] (lori.weeks@dal.ca), Erin Langman[1,2] (erin.langman@dal.ca), Sheri Roach[1,2] (sheri.roach@dal.ca), Morgan X. MacNeil[1,2] (morgan.macneil@dal.ca), Julie Caruso[1,2] (julie.caruso@dal.ca), Andrea C. Tricco[3,4,5] (andrea.tricco@unityhealth.to), Ba' Pham[6] (ba.pham@theta.utoronto.ca), Sharon Straus[3,7] (sharon.straus@utoronto.ca), Sujata Mishra[6] (sujata.mishra@mail.utoronto.ca), Wanrudee Isaranuwatchai[7,8,9] (wanrudee.i@hitap.net), Gordon V. Cormack[10] (gvcormack@uwaterloo.ca), Maura R. Grossman[10,11] (maura.grossman@uwaterloo.ca), Alexa R. Yakubovich[3,12] (alexa.yakubovich@dal.ca), Arezoo Mojbafan[1,2] (arezoo.mojbafan@dal.ca), Melissa Ignaczak[1,2] (melissa.ignaczak@dal.ca), Caron Leid[13] (caronleid@gmail.com), Jennifer Watt[6] (jennifer.watt@utoronto.ca), Susan Stevens[14] (susan.stevens@nshealth.ca), Tayaba Khan[13] (drtayabakhan@gmail.com), Janet Curran[1,2] (jacurran@dal.ca), Elaine Moody[1,2] (elaine.moody@dal.ca), Ricardo Rodrigues[15] (ricardo.rodrigues@iseg.ulisboa.pt)

CORRESPONDING AUTHOR: Marilyn Macdonald, Room 130, Forrest Bldg., Dalhousie University, PO Box 15000, 5869 University Avenue, Halifax NS B3H 4R2, 902-494-2433

1. Aligning Health Needs and Evidence for Transformative Change (AH-NET-C): A JBI Centre of Excellence, Halifax, Nova Scotia, Canada; 2. School of Nursing, Dalhousie University, Halifax, Nova Scotia, Canada; 3. Li Ka Shing Knowledge Institute, St. Michael's Hospital, Unity Health Toronto, Toronto, Canada; 4. Epidemiology Division and Institute of Health Policy, Management, and Evaluation, Dalla Lana School of Public Health, University of Toronto, Toronto, Ontario, Canada; 5. Queen's Collaboration for Health Care Quality: A JBI Centre of Excellence, Queen's University School of Nursing, Kingston, Ontario, Canada; 6. University of Toronto, Toronto, Ontario, Canada; 7. Institute of Health Policy, Management and Evaluation, University of Toronto, Toronto, Canada; 8. Unity Health Toronto, Toronto, Ontario, Canada; 9. Health Intervention and Technology Assessment Program (HITAP), Thailand; 10. David R. Cheriton School of Computer Science and School of Public Health Sciences, University of Waterloo, Waterloo, Ontario, Canada; 11. Osgoode Hall Law School, York University, Toronto, Ontario, Canada; 12. Department of Community Health and Epidemiology, Dalhousie University; 13. Patient and public partner; 14. Nova Scotia Health, Nova Scotia, Canada; 15. SOCIUS/CSG, ISEG (Lisbon School of Economics & Management), Universidade de Lisboa, Lisboa, Portugal

For peer review only

For peer review only - http://bmjopen.bmj.com/site/about/guidelines.xhtml

KEYWORDS: Long-Term Care; Aging; Health Care Costs; Sustainable Development

WORD COUNT: 4,848

ABSTRACT

Objectives: To identify, chart, and analyze the literature on recent initiatives to improve long-term care (LTC) coverage, financial protection and financial sustainability for persons aged 60 and older.

Design: Rapid scoping review

Data sources: Four databases and four sources of grey literature were searched for reports published between 2017 and 2022. After using a supervised machine learning tool to rank titles and abstracts, two reviewers independently screened sources against inclusion criteria.

Eligibility criteria: Studies published 2017-2022 in any language that captured recent LTC initiatives for people aged 60 and older, involved evaluation and directly addressed financing were included.

Data extraction and analysis: Data were extracted using a form designed to answer the review questions, and analyzed using descriptive qualitative content analysis, with data categorized according to a pre-specified framework to capture the outcomes of interest.

Results: Of 24 reports, 22 were published in peer-reviewed journals, and two were grey literature sources. Study designs included quasi-experimental, policy analysis or comparison, qualitative description, comparative case study, cross-sectional study, systematic literature review, economic evaluation, and survey. Studies addressed coverage based on level of disability, income, rural/urban residence, employment, and citizenship. Studies also addressed financial protection, including out-of-pocket (OOP) expenditures, co-payments, and risk of poverty related to costs of care. The reports addressed challenges to financial sustainability such

For peer review only

as lack of service coordination and system integration, insufficient economic development, and inadequate funding models.

Conclusions: Initiatives where LTC insurance is mandatory and accompanied by commensurate funding are situated to facilitate aging in place. Efforts to expand population coverage are common across the initiatives, with the potential for wider economic benefits. Initiatives that enable older people to access the services needed while avoiding OOP-induced poverty contribute to improved health and well-being. Preserving health in older people longer may alleviate downstream costs and contribute to financial sustainability.

ARTICLE SUMMARY

Strengths and Limitations of this Study

- This study uses the WHO definition of long-term care, which includes care across the continuum for older adults and does not exclude studies based on the type of care provided.
- This study identifies gaps in the existing literature and suggests areas for improvement, such as the importance of capturing equity measures.
- Due to the design of the review, long-established LTC programs and new initiatives that are yet to be evaluated may not be captured, as well as proprietary reports and government and health authority internal reports.
- Some of the included reports analyzed textual data from policy data in the absence of implementation data.

INTRODUCTION

The proportion of the global population aged 65 years and above is projected to grow from 10% in 2022 to 16% in 2050. (1) Due to rapidly aging populations, a greater prevalence of chronic conditions including dementia, gains in life expectancy, changing family dynamics and

For peer review only

living arrangements, and shortages of paid and unpaid caregivers, the demand for long-term care (LTC) services continues to expand. (2) Governments worldwide are challenged to create and adapt public programs to ensure that older adults have access to high quality LTC services. Accordingly, many governments undertook LTC system reforms.

In most countries, LTC services comprise a fraction of all healthcare expenditures. (3) The bulk of costs is covered by governments and social insurance with most people cared for in their own homes. (4) In general, systems of public LTC coverage can be clustered into two main types: 1) universal publicly funded system, and 2) a means-tested, safety-net system where public funding is available for those who cannot afford to pay for LTC. Universal LTC is available in many countries, including Japan, Germany, Luxembourg, the Netherlands, South Korea, Denmark, Finland, Norway, and Sweden. (5) Other countries, such as England and the United States, rely heavily on a means-tested, safety-net system. (5) The two main types of public LTC coverage can also be used in combination, as in the case of Canada, Austria, Australia, France, Greece, Ireland, Italy, Spain, Poland, New Zealand, and Switzerland. (5) A systematic review (6) noted that each country features its own distinct public LTC insurance (LTCI) implementation techniques and that public LTCI in all countries must continuously adapt to local conditions to function sustainably.

Researchers have noted the substantial variations in LTC funding approaches between low- and high-income regions, as well as the difficulty in finding relevant indicators and measures that can be adapted to different settings. Through a scoping review, Seyede and colleagues identified several indicators (e.g., OOP expenditures, geographic coverage, and proportion of older people receiving LTC) to measure progress towards Universal Health

For peer review only

Coverage in the context of population aging but they noted that many of these indicators would not be feasible in low- and middle-income countries. (7)

For the purposes of this review LTC includes service provision in the community, such as homecare and support, rehabilitative care, palliative care, residential care homes, assisted living facilities, nursing homes, as well as any other institutional setting. (8) LTC coverage not only includes care provided in facilities, it also includes programs such as cash benefits, community services in kind, day programs, residential facility care and home care. (8) According to WHO, "financial protection is achieved when direct payments made to obtain health services do not expose people to financial hardship and do not threaten living standards." (9 para.1) Financial protection via Universal Health Coverage is also a WHO Sustainable Development goal. (10) Financial sustainability “refers to the extent to which a given set of fiscal policies for LTC does not shift too large a financial burden on future generations (i.e., intergenerational fiscal equity) and ensures that “ends meet.” (11)

The focus of this scoping review is LTC funding, specifically, the knowledge gap regarding what is known about recent LTC initiatives that directly address financing. Addressing the complex needs of the world’s rapidly aging population will require a focus on improving aspects of LTC service coverage, financial protection, and financial sustainability. The primary objective of this scoping review is to identify, chart and analyze the literature on recent public initiatives (i.e., reforms, policies and programs) that have been undertaken to improve LTC coverage, financial protection and financial sustainability.

Review Question

What recent initiatives have been implemented to improve service coverage, financial protection and financial sustainability in the LTC sector for persons 60 years and older?

For peer review only

METHODS

This rapid scoping review was conducted in accordance with the JBI methodology for scoping reviews (12) and adheres to the reporting standards outlined in the Preferred Reporting Items for Systematic Reviews and Meta-Analyses extension for scoping reviews (PRISMA-ScR). (13) The WHO guide on rapid reviews (14) was consulted to help tailor the rapid review methods to decision-makers' needs. The Strategy for Patient Oriented Research – Evidence Alliance (SPOR-EA) processes for integrated knowledge translation were followed. (15-18) The WHO Centre for Health Development (WHO Kobe Centre) contracted this review. An agreement of work plan was provided to the contractor in lieu of a published protocol.

Search Strategy

After a limited search for seed articles in MEDLINE, two librarians (EL, JC) created an initial search strategy for MEDLINE that was peer-reviewed by a third librarian using Peer Review of Electronic Search Strategies (PRESS). (19) A librarian translated the search strategy for CINAHL, EMBASE and EconLit. Sources of grey literature included ClinicalTrials.gov, WHO International Clinical Trial Registry Platform (ICTRP) and ProQuest Dissertations and Theses. Canada's Drug and Health Technology Agency (CADTH) Grey Matters database was also used to identify additional sources of grey literature under the category of "health economics." All search strategies are available in the online supplemental file and are reported using Preferred Reporting Items for Systematic reviews and Meta-Analyses literature search extension (PRISMA-S). (20)

Inclusion and Exclusion Criteria

Studies published in any language, from January 2017 to August 25, 2022, that captured recent LTC initiatives for people aged 60 and older, involved evaluation and directly addressed

For peer review only

financing were included. Eligible study designs included randomized control trials (RCT), non-randomized controlled studies, before and after studies, interrupted time-series, quasi-experimental studies, qualitative studies, mixed methods, economic evaluation, systematic reviews and policy analysis reports. Reasons for exclusion of literature sources included failure to address coverage, quality, financial protection, and financial sustainability; no evaluation component, and study populations under age 60.

Study Screening and Data Extraction

Bibliographic records were identified for screening using the Continuous Active Learning® (CAL®) tool (21, 22) which uses supervised machine learning to rank titles and abstracts from most to least likely to be of interest. Results of the database searches were uploaded to Covidence (Veritas Health Innovation, Melbourne, Australia) and duplicates were removed. Three reviewers independently reviewed the first 1500 titles and abstracts. Potentially relevant full-text articles were then screened by two team members. All discrepancies were resolved by discussion or a third reviewer. The results of the search and the screening process are reported in full and presented in a Preferred Reporting Items for Systematic Reviews and Meta-Analyses (PRISMA) 2020 flow diagram. (23)

A data-extraction form was developed, and pilot-tested using a random sample of five included reports. Data were extracted on study characteristics, outcomes, and PROGRESS-Plus (24, 25) characteristics, where possible (see the online supplemental file). Reports written in languages other than English were reviewed and data extracted by team members fluent in those languages.

Quality Appraisal

For peer review only

Although quality appraisal is typically not conducted for a scoping review (14), it was conducted at the request of the commissioner. The appraisal was conducted by one team member and independently verified by a second team member. JBI methodological quality assessment tools (26-28) were selected based on the designs of included reports. Review articles were appraised using the Health Evidence™ Quality Appraisal Tool. (29) Discrepancies were resolved through team discussion.

Analysis

Using descriptive qualitative content analysis, (12) data was categorized according to the key outcomes of interest, coverage, financial protection and financial sustainability in LTC. Supplemental Table 1 outlines study characteristics, including initiatives and quality appraisal of included reports.

Patient and Public Involvement

Two public partners, co-authors, attended regular team meetings over a six-month period and provided input into the design of the study. They also reviewed and commented on report drafts, tools, and manuscript drafts, as well as dissemination activities.

RESULTS

The search retrieved 51,766 electronic database and grey literature records. Following screening at the title/abstract level, 100 reports were identified for full-text review (Figure 1). Of these, 24 reports met the inclusion criteria for this rapid scoping review.

Characteristics of Included Studies

Of the 24 reports, 22 were published in peer-reviewed journals, and two were grey literature sources. (30, 31) Just over half of the reports addressed initiatives in China (n=13). (32-44) The remainder originated in the United States (U.S., n=3) (45-47), Singapore (n=2) (48, 49),

For peer review only

Taiwan (n=2) (50, 51), and the Netherlands (n=1). (52) Three reports covered multiple countries. (30, 31, 53) All 24 reports were published between 2019 and 2022. The study designs included quasi-experimental (n=7) (32, 34, 35, 41, 43, 44, 48), policy analysis or comparison (n=6) (31, 38, 42, 49, 51, 53), qualitative research (n=3) (40, 47, 50), comparative case study (n=2) (30, 45), cross-sectional study (n=2) (33, 37), systematic literature review (n=2) (36, 39), economic evaluation (n=1) (52), and survey (n=1) (46) (see Supplemental Table 1).

Included studies analyzed secondary data (n=10) (30-33, 37, 41, 43, 44, 48, 52), policy documents (n=6) (36, 38, 39, 42, 51, 53), interviews (n=6) (33, 40, 45, 49, 50, 53) and survey data (n=3). (35, 46, 47) One study had data from both policy documents and interviews. (53)

All included sources focused on people aged 60 and above, but not all included data collected from older adults. Six reports included data from LTC stakeholders (40, 45-47, 50, 53). For example, Beauregard and Miller (45) conducted interviews with bureaucrats, consumer and provider advocacy groups, consultants and policy experts. For these six studies, we have indicated both who the data concerned and who provided the data (see Supplemental Table 1). Of the remaining 18 reports, nine (32-35, 37, 41, 43, 44, 48) reported findings from a LTC population and nine (30, 31, 36, 38, 39, 42, 49, 51, 52) focused on analyzing policy, comparing LTC systems across countries, or analyzing government spending on LTC (see Supplemental Table 1).

Quality Appraisal

Twenty-one out of 24 reports underwent critical appraisal. The three reports excluded from appraisal included two (30, 31) summarizing and interpreting existing studies and the third (42) used a design that approximated a quality appraisal. Overall studies were appraised at moderate to high in quality (see Supplemental Table 1).

For peer review only

210 **Long-Term Care Initiatives**

211 The LTC initiatives are described in Table 1.

212 **Table 1: LTC Initiatives**

Initiative	Country	Author Year	Description
LTCI Pilots	China	Chen et al. 2020 (33)<br>Chen & Ning 2022 (41)<br>Dai et al. 2022 (39)<br>Feng 2021 (38)<br>Han & Shen 2022 (40)<br>Lei et al. 2022 (32)<br>Liu & Hu 2022 (44)<br>Peng et al. 2022 (42)<br>Tang et al. 2022 (43)<br>Wu et al. 2020 (37)<br>Zhang & Yu 2019 (35)<br>Zhang et al. 2020 (34)<br>Zhou & Dai 2021 (36)	• Nearly universal health insurance coverage yet did not include community LTC coverage before the pilots<br>• LTCI pilots instituted to learn and plan how best to deliver LTC<br>• Began in 2016 with 16 cities and expanded to 49 cities by 2020<br>• Pilot cities explored multiple sources of financing and customized benefits packages based on their unique needs<br>• Aimed to promote formal (paid) care, while also encouraging informal care
CareShield Life	Singapore	Fong & Borowski 2022 (49)	• Legislated LTC social insurance<br>• Coverage for citizens and permanent residents born on or after January 1, 1980; entry age is 30; inclusive of people with severe disabilities and the economically disadvantaged<br>• Assessed for eligibility by the Ministry of Health (i.e., disability in performing at least three ADLs)<br>• Lifetime coverage; a claim can be made at any age<br>• Benefits increase annually by 2% for the first five years; premiums stop at age 67; the later a claim is made the higher the monthly payout
LTC 2.0 Reform	Taiwan	Chen & Fu 2020 (51)<br>Chiu et al. 2019 (50)	• Expansion of LTC to target aging in place<br>• Eligibility determined by assessing ADL, IADL, cognition, behavioural changes, rehabilitation, home situation, and caregiver stress<br>• Eligible populations: people ≥65 with ADL limitations as well as frailty; 2. people with dementia aged ≥50<br>• Changes included a reduction of co-payments (meaning the premium amount paid for services), a simplified contract system for service providers, modified care worker service fees, government subsidies for rural areas, family caregiver support centres, dementia support centres, and community care stations
Community First Choice	U.S.	Beauregard & Miller 2021 (45)	• The federal government program allowing states to expand Medicaid home and community-based services through the Affordable Care Act<br>• 6% federal increase in matching payments to shift spending from institutional to home-based care<br>• Provides individuals considered to be at an institutional level of care with ADL and IADL assistance

For peer review only - http://bmjopen.bmj.com/site/about/guidelines.xhtml

			• Agency-delivered or self-directed (clients take responsibility for hiring, supervising, and de-hiring workers)
LTC Reform	The Netherlands	Alders & Shut 2022 (52)<br>Hashiguchi & Llena-Nozal 2020 (30)<br>Kotschy & Bloom, 2022 (31)	• Reform initiated in response to high institutional care costs and focused on intensive home care as a substitute for institutional care<br>• Social care responsibility transferred to municipalities

LTCI=long-term care insurance; ADL=activities of daily living; IADL=instrumental activities of daily living

Equity

Although the term "gender" was used consistently, only biological sex (female-male) was reported. Nine studies reported gender (32-34, 37, 40, 41, 43, 44, 48) (42.3% male). Place of residence was occasionally unclear. Education level and socio-economic status, and age were reported in multiple ways based on the study design and variables included. Supplemental Table 2 reports the PROGRESS-Plus characteristics reported by study.

LTC Insurance Coverage

The LTCI pilots in China represented LTC in the home setting and in institutions. The countries involved in the remaining initiatives have been delivering LTC in home care and have moved to either enhance initiatives or to reform existing initiatives. Table 2 outlines the five initiatives, associated studies and a summary of coverage by initiative.

Table 2: Coverage by Country and Initiative

Initiative	Country	Author Year	Coverage	Coverage Effect
LTCI pilots	China	Dai et al. 2022 (39)<br>Feng et al. 2021 (38)<br>Han & Shen 2022 (40)<br>Lei et al. 2022 (32)<br>Peng et al. 2022 (42)<br>Zhou & Dai 2021 (36)	Eligible populations: seven pilot cities provided coverage to UEBM participants only. Eight cities expanded coverage to include: both urban and non-employees of UEBMI and URBMI (n=2); urban employees enrolled in UEBMI and rural and urban residents aged 60 and over enrolled in URBMI and NRCMS (n=1); all rural and urban participants in UEBM, UEBMI, and URBMI (n=5)	Eligibility criteria is determined by assessment of disability (assessment tools varied). People with severe disability were eligible in all pilot cities (32, 40), with some cities including coverage for moderate/mild disability (n=3) (32) and dementia (n=6) (40)

For peer review only

CareShield Life	Singapore	Fong & Borowski 2022 (49)	Eligible population: citizens and permanent residents born on or after 1 January 1980 who are $\geq$ age 30.	The overall assessment indicates benefits are modest and that depth of coverage is sacrificed to breadth.
LTC 2.0	Taiwan	Chen & Fu 2020 (51)	Eligible populations: people $\geq$65 with ADL limitations as well as frailty, and people with dementia aged $\geq$50.	Eligibility criteria determined by assessing ADL, IADL, cognition, behavioural changes, rehabilitation, home situation, and caregiver stress.<br>LTC includes health prevention and supportive community services; excludes institutional care.
Community First Choice	U.S.	Beauregard & Miller 2021 (45)	Eligible populations: Individuals who are Medicaid-eligible. States who adopt the program must provide services state-wide to everyone eligible.	Beauregard and Miller (45) found that states were concerned that this level of coverage would cause budgetary issues, and that "the added cost of CFC was not something that state officials were able or willing to shoulder."[p.184]
LTCI Reform	The Netherlands	Alders & Shut 2022 (52)	Eligible populations: Age 60 years and above. Social care transferred to municipalities, institutional care a federal responsibility, strict rules for eligibility.	Municipalities with low economic solvency directed those eligible to institutional care

UEBMI=Urban Employee Basic Medical Insurance; URBMI=Urban Resident Basic Medical Insurance; NRCMS=New Rural Cooperative Medical Scheme; CFC=Community First Choice; ADLs = activities of daily living; IADLs = instrumental activities of daily living

Initiative – LTC Pilots China

Six reports (32, 36, 38-40, 42) addressed coverage (Table 2) where near-universal health insurance coverage is provided through three social health schemes: the Urban Employee Basic Medical Insurance (UEBMI), the Urban Resident Basic Medical Insurance (URBMI) and the New Rural Cooperative Medical Scheme (NRCMS) for rural residents. The pilots across 15 cities in China were funded through these existing schemes and largely covered the severely disabled. (32, 36, 40) Unmet care needs related to ADL and IADL were common in the absence of LTCI. (32) Seven of the pilot cities provided coverage for urban residents only, while eight expanded to varying degrees to provide coverage to rural areas as well as the non-employed. (39) Eligible participants are covered for both institutional and home care, however, in some cities, coverage for institutional care was more comprehensive than for home care. (39, 40)

For peer review only

Initiative – CareShield Life Singapore

In 2019, Singapore implemented a mandatory social insurance scheme for citizens and permanent residents aged 30 and older born on or after January 1, 1980. Eligibility is determined by disability assessment (disability in performing at least three ADLs). CareShield Life (49) provides lifetime coverage, a claim can be made at any age, and premiums stop at age 67.

Initiative – Taiwan LTC 2.0 Plan

Taiwan's publicly funded LTC 2.0 Plan (51) is directed to aging in place, and institutional care is not included in the initiative. The program's successes included expanding coverage through the reduction of co-payments from 30% to 16% and waiving these for low-income users. The plan also includes family caregiver support centres as well as dementia support centres for rapid diagnosis and access to services. Community care stations offering healthy living programs focused on exercise and health self-management are part of the program. (51)

Community First Choice – U.S.

Medicaid-supported LTC services and supports excludes many older people with a disability, and that only 11% of older adults are Medicaid eligible (qualifiers include citizenship or permanent residency, income, household size, disability, family status and other factors). (47) Therefore, communities continuously fundraise to provide services to older people who do not qualify for Medicaid and cannot afford to purchase LTC services. (47) One recent initiative is the CFC Program (45) through which the federal government provides participating states with matching funding targeted at providing the services needed in the home setting to offset the need for institutional care. Many states are ideologically opposed to added government oversight (45) and/or may be fiscally unable to meet matching funding to maintain the program.

For peer review only

Initiative – Netherlands LTC Reform

The Netherlands has a long history of comprehensive public coverage of LTC (52), and a recent reform to address increasing costs involved shifting the financing of home care away from the central government to municipalities, while maintaining the financing of institutional care. Municipalities where the fiscal status is sound are well situated to manage the shift, while those that are not find themselves in the position of directing people with home care needs to institutional care which is still financed by the central government. This was an unintended effect of the reform.

Financial protection in LTC

Eleven reports (30-33, 40, 41, 43, 48, 49, 51, 52) addressed financial protection, two of which focused on OOP expenses. One (38) additional report contributed points relevant to financial protection. Table 3 outlines financial protection in relation to the five initiatives.

Table 3: Financial Protection Outcomes by Country and Initiative

Initiative	Country	Author Year	Financial protection outcomes
LTCI pilots	China	Chen & Ning 2022 (41)<br>Feng et al. 2021 (38)<br>Han & Shen 2022 (40)<br>Lei et al. 2022 (32)<br>Tang et al. 2022 (43)	• LTCI significantly reduced inpatient OOP expenditure, but not outpatient OOP expenditure. Individuals reporting fair health had higher OOP compared to those with poor health. (41)<br>• Existing medical schemes are being used to finance the pilots; shifting of funds to LTCI could drive up OOP costs for URBMI and NRCMS enrolees. (38)<br>• LTCI pilots do not financially protect rural residents, migrant workers, and employees in new forms of employment. Across pilots funding criteria vary with geography, occupation, and age. LTC institutions offer greater financial protection than home care.<br>• For each additional year of LTCI coverage, OOP medical expenses were reduced by 23.5%. (40)<br>• Implementation of LTCI decreased outpatient expenses (22.82%) and inpatient expenses (19.8%) in pilot cites compared to non-pilot cities. (43)
CareShield Life	Singapore	Fong & Borowski 2022 (49)	Includes lifetime protection against catastrophic spending associated with severe disability. Although the scheme is described as providing good basic LTC care needs protection there is an expectation that over time the depth of the protection will develop based on need.

For peer review only

LTC 2.0	Taiwan	Chen & Fu, 2020 (51)	Changes to the LTC system included a reduction of co-payments, reducing out-of-pocket spending for older adults.
Community First Choice	U.S.	Beauregard & Miller 2021 (45) Hashiguchi & Llena-Nozal 2020 (30)	In California, the costs of home care for older people with median income, no net wealth, and low care needs is not covered. It is also estimated that the public social protection system would not reduce the risk of income poverty for those earning a medium income with moderate care needs. (30)
LTCI reform	The Netherlands	Alders & Shut 2022 (52) Hashiguchi & Llena-Nozal 2020 (30) Kotschy & Bloom 2022 (31)	The use of asset-testing means that those without net wealth would have lower out-of-pocket costs than the median income for older people. Those with net wealth would still be able to afford out-of-pocket costs from their incomes alone. Public support only covers help with ADLs – for help with IADLs or social activities out of pocket costs can be up to 100%, depending on income and net wealth. (30)

276 LTCI = long term care insurance; OOP = out of pocket; URBMI = Urban Resident Basic Medical Insurance;
277 NRCMS = New Rural Cooperative Medical Scheme; ADLs = activities of daily living; IADLs = instrumental
278 activities of daily living

279 Initiative – LTCI Pilots China

280 Five reports directly (32, 33, 40, 41, 43) and one report indirectly (38) addressed financial
281 protection in relation to the LTCI Pilots in China. These reports addressed (OOP) health
282 expenditures (32, 41), the relationship between LTCI and the presence of LTC services (33);
283 population groups without protection (40); and LTCI impact on medical services utilization (43).
284 OOP health expenditures were statistically significantly reduced (23.5%) for inpatients but not
285 for outpatients because OOP rates favor the institutional setting. (43) Existing medical schemes
286 are being used to finance the pilots; shifting of funds to LTCI could drive up OOP costs for
287 enrolees. (38)

288 Filial responsibility in China traditionally meant that family members fulfilled caregiving
289 roles across the lifespan at rates bordering on 80%. (33) The initiation of LTCI is tied directly to
290 the availability of LTC services for ADLs and IADLs in both rural and urban settings. (33) There
291 was variability in who was considered eligible for LTCI. Groups such as migrant workers,

For peer review only

individuals working in new forms of employment and rural residents were often excluded. (40) Over time, there has been a gradual expansion to include these groups.

Cities piloting LTCI have experienced a decrease in medical services utilization due to a decrease in accessing both inpatient and outpatient services. Outpatient services use decreased by 22.8%, and inpatient services by 19.8%. (43) At the same time, non-pilot cities saw these percentages rise. These reductions in medical system utilization in the presence of LTCI hold promise for more optimal usage of the medical system, support for LTCI, and thus a strategic shifting of the costs from medical to social care.

Initiative – CareShield Life Singapore's LTCI

This initiative is an example of a universally publicly funded LTC scheme that extends to an entire population. (49) The scheme includes protection against catastrophic spending associated with severe disability and financial difficulty and nominal cash benefits with regular increases aimed at fostering consumer choice and flexibility, which can complement or substitute in-kind benefits and services. Although the scheme is described as providing good basic LTC care needs protection, there is an expectation that over time the depth of the protection will develop based on need.

Initiative – LTC 2.0 Taiwan

The reform of LTC undertaken in Taiwan (51) was targeted toward assisting the population of older people to age in place. The reform focused on the expansion of services that included but were not limited to dementia care, linking services such as adult daycare and respite services, family caregiver support centres, community health preventive care, links to discharge plans from hospitals and to home-based medical care. The reduction in insurance co-payments,

For peer review only

availability of LTC support services and the subsidies provided to rural areas demonstrate the attention paid to financial protection.

Community First Choice – U.S.

In recognition of the number of people ineligible for LTC via Medicaid in the U.S., the CFC Program (45) was conceptualized as an opportunity for individual states to consider protecting more of their older residents with social care needs from income poverty. Hashiguchi found that in California, even with social care systems in place, “the out-of-pocket costs for older people with mean net wealth would be higher than their incomes” (p. 32), and that the costs of home care for those with median income, no net wealth, and low care needs would not be covered, highlighting the risk of income poverty among older adults. (30)

Initiative – Netherlands LTC Reform

The LTC insurance system in the Netherlands guarantees no older person experiences income poverty, despite OOP costs for low care needs (30), because copayments are means-tested to reduce the economic burden for the poor. (31) Municipalities that were unable to fund home-care recipients over the long term directed them to institutional care. Although institutional care would financially protect care recipients, those choosing to remain in their home risk their financial protection. To counteract this, municipalities have been able to negotiate substantial reductions in maximum co-payments since 2019. (52)

Financial sustainability in LTC

Seven reports (34, 36, 40, 42, 45, 49, 52) directly address financial sustainability and three (38, 39, 50) peripherally. Table 4 portrays financial sustainability in relation to the five initiatives.

Table 4: Financial Sustainability by Country and Initiative

For peer review only

Initiative	Country	Author, Year	Financial sustainability findings
LTCI pilot	China	Dai et al. 2022 (39)<br>Feng et al. 2021 (38)<br>Han & Shen 2022 (40)<br>Peng et al. 2022 (42)<br>Zhang et al., 2020 (34)<br>Zhou & Dai 2021[38]	• Integrating LTCI into the existing medical insurance framework is favored to reduce administrative costs, address basic LTC needs and gradually grow programs as financing permits. "The financial sustainability of the LTCI programmes has been questionable. A main reason for this is that the value allowed to be extracted from medical insurance foundations is limited." (39 p.196)<br>• Diversification of funding sources such as cash benefits to support family caregivers and in-kind services may be necessary based on pilot funding in some cities. Such diversification is offered as a path toward sustainability. (39)<br>• Glinskaya et al. advocates for the gradual increase in individual contributions in tandem with increased financial support from central and local governments to attain sustainability. (38)<br>• Funding sources affect sustainability and most cities have more than one source. Primary reliance is on transfers from the medical insurance fund, other sources include individual contributions, organizational contributions, financial subsidies, and social donations. (40)<br>• The Policy Modelling Consistency index was employed to measure the interaction coupling coordination degree between LTCI policy, population aging and economic development to assess the sustainability of the LTC pilot system. Shanghai reached the excellent level, Nantong achieved good coordination and the remaining cities' coordination degree was basic. The coordination degree between LTCI policy, population aging and economic development was reported as low or basic and suggests that economic development is not where it needs to be to sustain LTC financing. Sustained economic growth in tandem with population ageing and modifiable LTCI policies are essential to the financial sustainability of LTCI. (42)<br>• A general lack of system integration was identified and believed to place financial sustainability in jeopardy. (36)<br>• LCTI reduced informal/family caregiver care (34)<br>• Satisfaction with formal care was high. (34)<br>• Continued expansion of LTCI is favoured (34)
CareShield Life	Singapore	Fong & Borowski 2022 (49)	Policy analysis reported CareShield Life represents a collectivist (everyone contributes) approach to LTC Social Insurance; financial sustainability is guarded by rigorous claims criteria and eligibility assessments; inclusion of younger age groups contributes to affordability (coverage begins at age 30); and depth in service provision is sacrificed for breadth. Financial forecasting of the scheme is recommended to address sustainability.
LTC 2.0	Taiwan	Chiu et al. 2019 (50)	Examined difficulty with implementation to LTC 2.0 and challenges to sustainability.
LTCI reform	The Netherlands	Alders & Shut 2022 (52)	LTC reform initiated to address high institutional care costs. Reform focused on intensive home care as a substitute for nursing homes/institutional care. Social care was transferred to municipalities. The unanticipated effect was a cost-shifting by municipalities to the central government, which funds Public LTCI (specifically institutional care). This economic evaluation

For peer review only - http://bmjopen.bmj.com/site/about/guidelines.xhtml

Initiative	Country	Author, Year	Financial sustainability findings
			study examined cost shifting with a focus on the solvency rate of municipalities. Results: Municipalities with low solvency (20%) had 3.79 more admissions to Public LTCI per 10,000 population over age 65, equivalent to 2.5% more admissions than average. Urban municipalities with low financial solvency, a higher population over age 80 living alone or with limitations, and with a lower income had a significantly higher number of admissions to the Public LTCI system. Authors suggest shifting can be addressed by risk adjusting their social support funding based on the proportion of older people by municipality and on service needs. Such approaches optimize the use of funding sources and location of care and contribute to system sustainability.
Community First Choice	U.S.	Beauregard & Miller 2021 (45)	CFC was developed as an alternative to long-term institutional care. Participating states receive a 6% additional federal match to transition from Medicaid home and community-based services benefits. This comparative case study (2 states with CFC and 1 without) examined factors that influenced adoption. Factors included leadership from the state Medicaid office, ability to meet the 6% matching, LTC advocacy, ideology related to federal government involvement. A state government advocate stated, "we have heard states really express concerns about the budgetary implications of this and whether it be sustainable even with the 6% increased FMAP [Federal Medical Assistance Percentage]."p. 184

LTCI=long-term care insurance; CFC=Community First Choice

Initiative – LTC Pilots China

Several studies (36, 40, 42) indicated challenges to the sustainability of China's LTCI pilots. The challenges reported were a lack of system integration (36), reliance on transfers from the medical insurance fund (39, 40), and a need for policy planning to address population aging and aligns with economic development. (42) Feng et al. (38) advocated for the gradual increase in individual contributions in tandem with increased financial support from central and local governments to attain sustainability. In addition, Zhang et al. (34) conducted a survey of care recipients and their family caregivers in Shanghai to determine the impact of LTCI on informal care hours provided by family members. Informal/family caregiver care was reduced by 0.47 hours. Across the 407 families, a weekly average decrease of 12.36 hours of informal care was reported. Satisfaction with formal care was 72%. These results speak favorably for the LTCI pilots to alleviate the burden on carers and the continued expansion of LTCI.

For peer review only

Initiative – CareShield Life Singapore's LTCI

A policy analysis (49) of CareShield Life which is a mandatory scheme spreads risk across the age cohorts, while including a pre-funding component in which enrolees pay forward their future LTC needs, thus contributing to sustainability. It is estimated that less than 0.1% of the population aged 30-40 years have severe disabilities, therefore this insurance scheme is projected to not only meet LTC needs but also the needs of younger persons who may require services. These authors recognized the collectivist approach to this mandatory LTCI scheme, noting however that depth is sacrificed for breadth, and the need to conduct financial forecasting of the scheme to address sustainability.

Initiative – LTC 2.0 Taiwan

Chiu et al. addresses coverage peripherally in a qualitative descriptive study of the implementation of LTC 2.0. (50) They found that the mechanisms for service integration needed to be fully implemented and also recommended building trust between service delivery agencies and government, clarifying roles and responsibilities of government care managers and agency care managers, the development of an integrated information system, and the establishment of a budget tracking system.

Initiative – LTCI Reform the Netherlands

LTC reform (in 2015–2019) was initiated in response to high institutional care costs and focused on intensive home care as a substitute for nursing home/institutional care. Social care responsibility in the home was transferred to municipalities. Alders and Schut (52) conducted an economic evaluation of municipal-level data and estimated a random-effects model to explain variation across municipalities and to report the numbers of people admitted to LTC institutions annually. The examination of municipality solvency was part of the evaluation. Municipalities

For peer review only - http://bmjopen.bmj.com/site/about/guidelines.xhtml

with low solvency (20%) had 3.79 more admissions to Public LTCI per 10,000 population over age 65, equivalent to 2.5% more admissions than average. The authors suggest that shifting can be addressed by risk-adjusting their social support funding based on the proportion of older people by municipality and service needs. Such approaches optimize the use of funding sources and location of care and contribute to system sustainability.

Initiative – Community First Choice program in the U.S.

The CFC program offers a 6% federal increase in matching payments to shift spending from institutional to home-based care for individuals considered to be at an institutional level of care. Adoption and sustainability of the program are contingent upon federal home and community-based services policy that considers implications at the state level. Higher federal match rates and reducing the state share of Medicaid spending would create the conditions for expansion and sustainability. (46) A case study of two states with the CFC program (Maryland and Texas) and one state without (Oklahoma) (45) reported factors influencing the adoption of the CFC program included state ideology, fiscal status, advocacy for LTC, Medicaid leadership and the strength of existing home and community-based services.

DISCUSSION

Thirteen of the 24 papers included in this review addressed LTCI initiatives in China. Coincidentally China initiated 16 pilot studies of these initiatives in 2016 and evaluations of several of the pilots occurred in the five-year window of our inclusion criteria for LTCI initiatives that were evaluated. The challenges and learnings from these pilots were common across the dataset and not deemed as unique to China. We found that, apart from the CFC program, all initiatives either provided coverage to the entire population of the country or were working incrementally toward providing coverage to the entire population. Also common was

For peer review only

the quest across initiatives to discern how to fund them. Older people with substantive care needs were the most likely to be eligible for services across all initiatives.

Although initiatives differed across the various countries, most entailed an increase in coverage. Strategies to increase coverage included expansion of eligibility criteria, reducing or waiving co-payments based on income, and simplifying processes for home support service providers. These strategies support aging in place as do initiatives aimed at health promotion that assist older people in maintaining if not improving their physical and cognitive abilities.

The findings related to financial protection include strategies such as tackling OOP expenses and providing home support services to support aging at home, mandating LTC, providing caregiver support and striking a balance between meeting LTC needs and how to fund them. These strategies saw a decrease in accessing both outpatient and inpatient medical services and access to LTC across the lifespan. A strategy that may help in the short and medium term is to develop an equitable LTC recipient dose sliding scale using ADLs, IADLs, cognition, behavioural changes, rehabilitation, home situation and caregiver stress. A sliding scale approach with individual contextualized person-focused assessments represents a strategy to push toward universalism, advance defamilization and restrain marketization (54) through optimal care to client matching. A sliding-scale approach calls for LTC systems that are dynamic and that can increase or decrease supports and services based on changes in individual needs.

Financial sustainability was a goal across all initiatives. Evidence of this was found in the evaluation of the LTCI pilots that stressed the need for system integration, dedicated funding, controlling OOP costs, and projecting LTC needs in relation to population aging and economic development. Furthermore, Singapore mandated LTC and structured the scheme to enroll everyone at age 30, paying premiums until age 67 and ensuring sustainability. Lastly the

For peer review only

Netherlands reform altered the funding plan to cost share with municipalities. In doing so, they discovered they needed to adjust the proportion of cost sharing based on the proportion of older people per municipality and service needs to address sustainability.

The findings of this review call forth the need to reflect on traditional approaches to LTC such as home care and institutional care. An alternate model could focus on enhanced services to support older adults to remain living in their homes and communities. A continuum that begins with remaining at home all the way to institutional care with more home-approximating options such as co-housing (55) may support optimal independence over a longer timeframe. This thinking is conceptually underpinned by the notion of providing the right support, in the right amount, at the right time to delay decline and to push down projections on the amount of care required, thereby contributing to financial sustainability. Such a continuum could contribute to positively addressing age-related inflation, which occurs when a group consumes more goods and services than it produces as they age. (56, 57) The control of the need for services combined with an increasing workforce dampens inflation and improves the economic growth required to sustain health and social initiatives. Health promotion can slow the decline in health thus delaying the need for supports and services.

STRENGTHS AND LIMITATIONS

This team has breadth and depth in review methodology and the conduct of reviews as well as experts in long-term care. The searches were extensive including four databases and multiple sources of grey literature, and the searches were peer reviewed. There were no language limits imposed on the searches to capture as many titles and abstracts as possible. This rapid scoping review was limited to identifying initiatives (programs, policies or reforms) implemented and evaluated in the past five years, in LTC for people aged 60 and above.

For peer review only

Countries need to include mechanisms within their initiatives to capture evaluation data. In the evidence assessed, LTC initiatives were focused on launching as opposed to evaluation, and we expect to see more evaluation including quality addressed in future research. Undoubtedly there are initiatives that do not appear in this report because they did not meet all the inclusion criteria. In addition, there is commonly a lag between initiatives, their evaluation and subsequent publication. There is always the possibility that, despite the number of database searches conducted, the keywords used in the searches may not have been used in a relevant publication. Many countries have long-established LTC programs; however, unless a major modification occurred and met the inclusion criteria for this review, it would not have been captured. Some of the included reports analyzed textual data from policy data in the absence of implementation data.

RESEARCH GAPS

We believe there are recent LTC initiatives in place that have not been evaluated or have been evaluated but not published, and that such evidence needs to be published for all countries/sub-nations to benefit from. The number of economic evaluations of LTC initiatives was limited, and such evaluations should be encouraged to inform evolution in LTC. In addition to evaluation implementation, data from the initiatives is important to know what works and where improvements are needed to contribute to sustainability.

Although it may have been the intent of some researchers to capture equity measures, this was not necessarily made explicit in their reports. Absent from the data are race, religion, and identification of any gender minority groups. Discussion relating the characteristics to study results/findings was limited. Consistent use of a conceptual framework to compare policies such

For peer review only - http://bmjopen.bmj.com/site/about/guidelines.xhtml

as that proposed by Noda et al. (53), is suggested to inform comparative evaluation studies or single initiative evaluation.

CONCLUSION

The initiatives related to coverage, financial protection and financial sustainability identified in this review came from high, upper-middle, lower-middle and low-income[13] and hold hope for the growth of existing initiatives and their expansion within and beyond their respective countries. The ones pertaining to lower-middle and low-income economies in particular fill a gap in a body of literature that has focused on high-income countries. Initiatives where LTCI is mandatory and accompanied by a commensurate funding scheme are situated to facilitate older people aging in place. Efforts to expand population coverage are common across the initiatives, with the potential for wider economic benefits. In addition, initiatives that enable older people to access services while avoiding OOP-induced poverty contribute to improved health and well-being. Preserving health in older people longer may alleviate downstream costs and contribute to financial sustainability. The relevance and timeliness of LTC initiatives cannot be understated because by 2050, 80% of older people will live in low and middle-income countries. (58)

ETHICS STATEMENTS

Patient consent for publication

Not applicable.

ACKNOWLEDGEMENTS

The authors are grateful to Dalhousie University Librarian Louise Gillis, who completed the peer review of the search strategy.

For peer review only

For peer review only - http://bmjopen.bmj.com/site/about/guidelines.xhtml

FOOTNOTES

Contributors: Design of the review: MM, LEW, ACT, EL, BP, GVC, MRG, BP, SS, CL, TK, ARY; Data collection: MM, LEW, EL, AM, MI, BP, JC; Critical appraisal: MM, LEW, BP; Data analysis and interpretation: MM; Drafting the article: MM, EL, JC, SR, MXM; Critical revisions of the article: MM, LEW, EL, JC, ACT, BP, SS, SM, WI, GVC, MRG, ARY, AM, MI, CL, JW, SS (NSH), TK, DL, JC, EM, RR, SR, MXM. All authors approved the final version to be published.

Competing interests: LEW is the co-owner of an assisted living home.

Funding: This research was supported by the World Health Organization Centre for Health Development (WHO Kobe Centre – WKC: K22005).

Data sharing: All search strategies are available via searchRxiv (see online supplemental file). All additional data relevant to the study are included as supplementary information.

Ethics approval: Ethics approval not required in this rapid scoping review.

For peer review only

References

1. United Nations Department of Economic and Social Affairs. World population prospects 2022: Summary of results. 2022. https://www.un.org/development/desa/pd/sites/www.un.org.development.desa.pd/files/undesa_pd_2022_wpp_key-messages.pdf.

2. Costa-Font J, Courbage C, Swartz K. Financing long-term care: Ex ante, ex post or both? Health Econ 2015;24(S1):45-57. doi: 10.1002/hec.3152

3. Grignon M, Spencer BG. The funding of long-term care in Canada: What do we know, what should we know? Can J Aging 2018;37(2):110-20. doi: 10.1017/S0714980818000028

4. OECD. Spending on long-term care. 2020. https://www.oecd.org/health/health-systems/Spending-on-long-term-care-Brief-November-2020.pdf.

5. Feng Z, Glinskaya E. Aiming Higher: Advancing public social insurance for longterm care to meet the global aging challenge; Comment on "Financing long-term care: Lessons from Japan". Int J Health Policy Manag 2020;9(8):356-59. doi: 10.15171/ijhpm.2019.121

6. Chen L, Zhang L, Xu X. Review of evolution of the public long-term care insurance (LTCI) system in different countries: Influence and challenge. BMC Health Services Research 2020;20:1057. doi: 10.1186/s12913-020-05878-z

7. Hosseini Jebeli SS, Aziz R, Megumi R, et al. Measuring universal health coverage to ensure continuing care for older people: A scoping review with specific implications for the Iranian context. Eastern Mediterranean Health Journal 2021;27(8):806-17. https://apps.who.int/iris/handle/10665/353217.

8. World Health Organization. Framework for countries to achieve an integrated continuum of long-term care. 2021. https://apps.who.int/iris/handle/10665/349911.

9. World Health Organization. Financial protection. https://www.who.int/health-topics/financial-protection.

[dataset] 10. World Health Organization. SDG Target 3.8: Achieve universal health coverage, including financial risk protection, access to quality essential health-care services and access to safe, effective, quality and affordable essential medicines and vaccines for all. https://www.who.int/data/gho/data/themes/topics/indicator-groups/indicator-group-details/GHO/sdg-target-3.8-achieve-universal-health-coverage-(uhc)-including-financial-risk-protection.

11. Colombo F, Llena-Nozal A, Mercier J, et al. Help wanted? Providing and paying for long-term care. 2011. https://www.oecd-ilibrary.org/content/publication/9789264097759-en.

12. Peters M, Godfrey C, McInerney P, et al. Chapter 11: Scoping Reviews (2020 version). In: Aromataris E, Munn, Z, eds. JBI Manual for Evidence Synthesis 2020. doi: 10.46658/JBIMES-20-12

13. Tricco A, Lillie, E, Zarin, W, et al. PRISMA extension for scoping reviews (PRISMA-ScR): Checklist and explanation. Annals of Internal Medicine 2018;169(7):467-73. doi: 10.7326/m18-0850

14. Tricco AC, Langlois EV, Straus SE. Rapid reviews to strengthen health policy and systems: A practical guide. Geneva: World Health Organization 2017. https://apps.who.int/iris/bitstream/handle/10665/258698/9789241512763-eng.pdf.

15. Tricco AC, Zarin W, Clement F, et al. Introducing the Strategy for Patient Oriented Research (SPOR) Evidence Alliance: A partnership between researchers, patients and health system decision-makers to support rapid-learning and responsive health systems in Canada and beyond. Facets 2022;7:639-53. doi: 10.1139/facets-2021-0127

16. Lunny C, Zarin W, Chaudhry S, et al. An inclusive and diverse governance structure of the strategy for patient-oriented research (SPOR) Evidence Alliance. Facets 2022;7:435-47. doi: 10.1139/facets-2021-0129

17. Zarin W, Lunny C, Chaudhry S, et al. A Canadian model for providing high-quality, timely and relevant evidence to meet health system decision-maker needs: The SPOR Evidence Alliance. Facets 2022;7:420-34. doi: 10.1139/facets-2021-0132

18. Li LC, Hoens AM, Wilhelm L, et al. Patient engagement in the SPOR Evidence Alliance: Reflection and learnings. Facets 2022;7:126-38. doi: 10.1139/facets-2021-0133

19. McGowan J, Sampson M, Salzwedel DM, et al. PRESS peer review of electronic search strategies: 2015 guideline statement. Journal of Clinical Epidemiology 2016;75:40-6. doi: 10.1016/j.jclinepi.2016.01.021

20. Rethlefsen ML, Kirtley S, Waffenschmidt S, et al. PRISMA-S: An extension to the PRISMA statement for reporting literature searches in systematic reviews. Syst Rev 2021;10(1):39. doi: 10.1186/s13643-020-01542-z

21. Pham B, Rios P, Radhakrishnan A, et al. Comparative-effectiveness research of COVID-19 treatment: A rapid scoping review. BMJ Open 2022;12(6):e045115. doi: 10.1136/bmjopen-2020-045115

22. Rios P, Radhakrishnan A, Williams C, et al. Preventing the transmission of COVID-19 and other coronaviruses in older adults aged 60 years and above living in long-term care: A rapid review. Syst Rev 2020;9(1):218. doi: 10.1186/s13643-020-01486-4

23. Page MJ, McKenzie JE, Bossuyt PM, et al. The PRISMA 2020 statement: An updated guideline for reporting systematic reviews. BMJ 2021;372:n71. doi: 10.1136/bmj.n71

24. The Campbell and Cochrane Equity Methods Group. PROGRESS-Plus. https://methods.cochrane.org/equity/projects/evidence-equity/progress-plus.

25. O'Neill J, Tabish H, Welch V, et al. Applying an equity lens to interventions: Using PROGRESS ensures consideration of socially stratifying factors to illuminate inequities in

For peer review only

health. Journal of Clinical Epidemiology 2014;67(1):56-64. doi: 10.1016/j.jclinepi.2013.08.005

26. Gomersall JS, Jadotte YT, Xue Y, et al. Conducting systematic reviews of economic evaluations. Int J Evid Based Healthc 2015;13(3):170-78. doi: 10.1097/xeb.0000000000000063

27. Moola S, Munn Z, Tufanaru C, et al. Chapter 7: Systematic reviews of etiology and risk. In: Aromataris E, Munn, Z eds. JBI Manual for Evidence Synthesis 2020. https://synthesismanual.jbi.global.

28. Tufanaru C, Munn, Z, Aromataris, E, Campbell, J, Hopp, L. Chapter 3: Systematic reviews of effectiveness. In: Aromataris E, Munn, Z eds. JBI Manual for Evidence Synthesis 2020. https://synthesismanual.jbi.global.

29. National Collaborating Centre for Methods and Tools. Health evidence quality assessment tool. 2018. https://www.nccmt.ca/knowledge-repositories/search/275.

30. Hashiguchi TCO, Llena-Nozal A. The effectiveness of social protection for long-term care in old age: Is social protection reducing the risk of poverty associated with care needs? OECD Publishing 2020. https://EconPapers.repec.org/RePEc:oec:elsaad:117-en.

31. Kotschy R, Bloom D. A comparative perspective on long-term care systems [Working paper 29951]. National Bureau of Economic Research 2022. doi: 10.3386/w29951

32. Lei X, Bai C, Hong J, et al. Long-term care insurance and the well-being of older adults and their families: Evidence from China. Social Science & Medicine 2022;296:114745. doi: 10.1016/j.socscimed.2022.114745

33. Chen L, Zhang X, Xu X. Health insurance and long-term care services for the disabled elderly in China: Based on CHARLS data. Risk Management and Healthcare Policy 2020;13:155-62. doi: 10.2147/RMHP.S233949

34. Zhang H, Zhang X, Zhao Y, et al. Impact of formal care use on informal care from children after the launch of long-term care insurance in Shanghai, China. International Journal of Environmental Research and Public Health 2020;17(8): 2938. doi: 10.3390/ijerph17082938

35. Zhang Y, Yu X. Evaluation of long-term care insurance policy in Chinese pilot cities. International Journal of Environmental Research and Public Health 2019;16(20): 3826. doi: 10.3390/ijerph16203826

36. Zhou W, Dai W. Shifting from fragmentation to integration: A systematic analysis of long-term care insurance policies in China. International Journal of Integrated Care 2021;21(3):11. doi: 10.5334/ijic.5676

37. Wu J, Chen S, Wen H, et al. Health status, care needs, and assessment for beneficiaries with or without dementia in a public long-term care insurance pilot in Guangzhou, China. BMC Health Services Research 2020;20(1):1127. doi: 10.1186/s12913-020-05965-1

For peer review only

38. Feng Z, Zhuang X, Glinskaya E. Understanding China's long-term care insurance pilots: Key features, challenges and prospects. China: An International Journal 2021;19(3):168-87. doi: 10.1353/chn.2021.0034

39. Dai W, Li Y, Shen J. The pilot programme of long-term care insurance in China: Fragmentation and policy implications. China: An International Journal 2022;20(2):186-206. doi: 10.1353/chn.2022.0010

40. Han Y, Shen T. Long-term care insurance pilot programme in China: Policy evaluation and optimization options-taking the pilot programme in the northeast of China as an example. International Journal of Environmental Research and Public Health 2022;19(7): 4298. doi: 10.3390/ijerph19074298

41. Chen H, Ning J. The impacts of long-term care insurance on health care utilization and expenditure: Evidence from China. Health Policy and Planning 2022;37(6):717-27. doi: 10.1093/heapol/czac003

42. Peng R, Deng X, Xia Y, et al. Assessing the sustainability of long-term care insurance systems based on a policy-population-economy complex System: The case study of China. International Journal of Environmental Research and Public Health 2022;19(11):6554. doi: 10.3390/ijerph19116554

43. Tang Y, Chen T, Zhao Y, et al. The Impact of the long-term care insurance on the medical expenses and health status in China. Frontiers in Public Health 2022;10:847822. doi: 10.3389/fpubh.2022.847822

44. Liu H, Hu T. Evaluating the long-term care insurance policy from medical expenses and health security equity perspective: Evidence from China. Archives of Public Health 2022;80:3. doi: 10.1186/s13690-021-00761-7

45. Beauregard LK, Miller EA. A comparative analysis of state adoption of the Community First Choice program. Home Health Care Services Quarterly 2021;40(3):177-91. doi: 10.1080/01621424.2021.1947926

46. Koumoutzis A, Heston-Mullins J, Mayberry PS, et al. If you ask them, they will support: A national study of local initiatives developed to provide social care to older adults in the community. Journal of Applied Gerontology 2022;41(8):1860-69. doi: 10.1177/07334648221090945

47. Koumoutzis A, Stemen SE, Maharjan R, et al. Local initiatives to fund services for older americans: community recognition of the importance of social care. Journal of Applied Gerontology 2021;40(9):980-84. doi: 10.1177/0733464820944325

48. Sum G, Kadir MM, Ho SH, et al. Cost analysis of a Patient-Centred Medical Home for community-dwelling older adults with complex needs in Singapore. Annals Academy of Medicine Singapore 2022;51(9):553-66. doi: 10.47102/annals-acadmedsg.2022165

For peer review only

49. Fong JH, Borowski A. Long-term care insurance reform in Singapore. Journal of Aging & Social Policy 2022;34(1):73-90. doi: 10.1080/08959420.2021.1926867

50. Chiu T-Y, Yu H-W, Goto R, et al. From fragmentation toward integration: A preliminary study of a new long-term care policy in a fast-aging country. BMC Geriatrics 2019;19(1):159. doi: 10.1186/s12877-019-1172-5

51. Chen C-F, Fu T-H. Policies and transformation of long-term care system in Taiwan. Annals of Geriatric Medicine and Research 2020;24(3):187-94. doi: 10.4235/agmr.20.0038

52. Alders P, Schut FT. Strategic cost-shifting in long-term care: Evidence from the Netherlands. Health Policy 2022;126(1):43-48. doi: 10.1016/j.healthpol.2021.11.008

53. Noda S, Hernandez PMR, Sudo K, et al. Service delivery reforms for Asian ageing societies: A cross-country study between Japan, South Korea, China, Thailand, Indonesia, and the Philippines. International Journal of Integrated Care 2021;21(2):1. doi: 10.5334/ijic.4739

54. Ranci C, Pavolini E. Not all that glitters is gold: Long-term care reforms in the last two decades in Europe. Journal of European Social Policy 2015;25(3):270-85. doi: 10.1177/0958928715588704

55. Canadian Cohousing Network. Home. https://cohousing.ca/.

56. Petersen T, López L. Demographics and foreign trade: What are the effects of an aging population on the current account balance of an economy? Bertelsmann Stiftung 2019. https://globaleurope.eu/globalization/effects-of-aging-population/.

57. Yoshino N, Kim CJ, Sirivunnabood P. Aging population and its impacts on fiscal sustainability. T20 Japan 2019. https://t20japan.org/policy-brief-aging-population-impacts-fiscal-sustainability/.

58. World Health Organization. Ageing and Health 2022. https://www.who.int/news-room/fact-sheets/detail/ageing-and-health.

For peer review only

Figure 1: PRISMA flow diagram of article selection

[1] Three reviewers initially independently screened 500 records using CAL® and a combined screening sample of 1970 for a total screening sample of 3470 records. With an estimate of duplicate records of 8.4% among the first 1500, we estimate humans screened a total of 3179 records using CAL®.

For peer review only

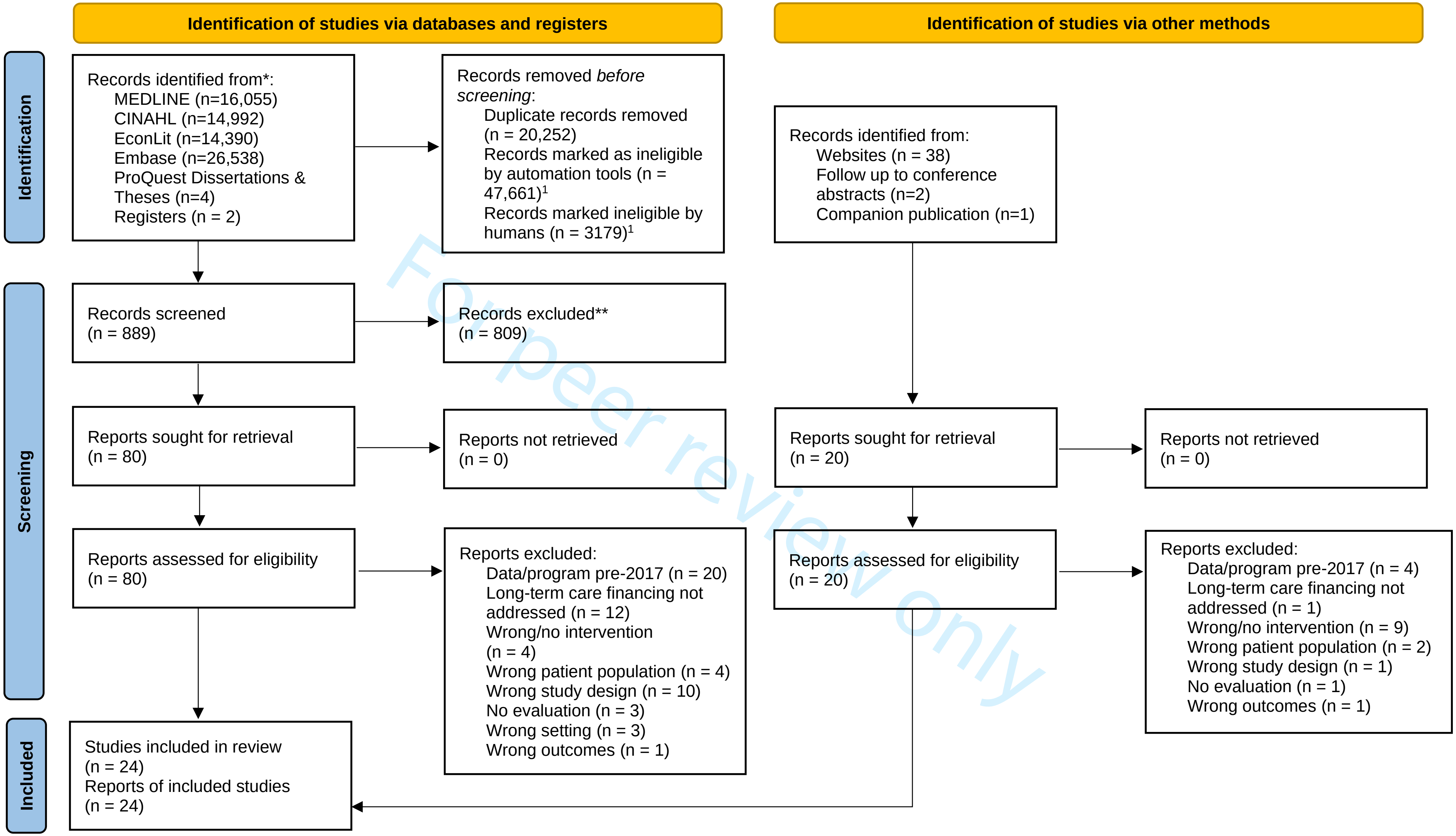

For peer review only

Table 1: Study Characteristics

Author Year	Country	Initiative	Design	Aim	Population and Data Sources	Quality Appraisal
Alders & Shut 2022(54)	The Netherlands	LTCI reform	Economic evaluation	To discern if municipalities with a low solvency rate (<20 percent) are more likely to engage in strategic cost shifting and if this resulted in an overall upward trend in admission rates to LTC services covered by public LTC services.	People aged 65+ in the Netherlands; data from 327 out of 355 municipalities from 2014–2019.<br><br>Data source(s): Monitor Long-term Care (Monitor Langdurige Zorg); Online dashboard by the Ministry of the Interior; Statistics Netherlands	Medium
Beauregard & Miller 2021(47)	U.S.	Community First Choice program	Comparative case study (qualitative)	To understand state processes related to the adoption of the Medicaid Community First Choice (CFC) Program and to identify factors that influenced states' decisions to adopt CFC.	LTC population: Medicaid-eligible older adults in Texas, Oklahoma, Maryland, and nationally (U.S.).<br><br>Study population: Data collected from n=46 individuals in roles as follows: federal and state bureaucrats, consumer and provider advocacy groups, consultants, and policy experts. Maryland (n=14), Texas (n=14), Oklahoma (n=9), and national (n=9) level participants.<br><br>Data source(s): Interviews	Medium
Chen et al. 2020(35)	China	LTCI pilots	Cross-sectional study	To evaluate the influence of health insurance and other factors on the availability of LTC services.	n=7787; Urban: n=3945; Rural: n=3842; CHARLS (2015) database adults aged 45 and up; the sample is aged 60 and up; CHARLS (2015) covered more than 10,000 families and 17,500 individuals from 150 districts of 28 provinces.<br><br>Data source(s): China Health and Retirement Longitudinal Study (CHARLS) database, 2015	Medium
Chen & Fu 2020(53)	Taiwan	LTC 2.0 reform	Policy analysis	To review the development of LTC policies, present strategies for expanding LTC, and outline policy suggestions and implications.	Older adults in Taiwan. The proportion of the population aged 65 years and over in Taiwan has doubled from 7% in 1993 to 14% in 2018. The number of older adults receiving LTC services increased from 9,148 in 2008 to	High

For peer review only

Author Year	Country	Initiative	Design	Aim	Population and Data Sources	Quality Appraisal
					258,351 in 2019, which only accounts for 41.47% of the population needing LTC.<br><br>Data source(s): Policy documents; government data	
Chen & Ning 2022(43)	China	LTCI pilot	Quasi-experimental design	To examine the policy treatment effect of long-term care insurance (LTCI) on health care utilization and OOP health expenditure (OOP) in China.	N=20,814<br>Participants in the China Health and Retirement Longitudinal Study (CHARLS).<br><br>Data source(s): China Health and Retirement Longitudinal Study (CHARLS) longitudinal study, 2018	High
Chiu et al. 2019(52)	Taiwan	LTC 2.0 reform	Qualitative research	To examine the experiences and challenges of integration for LTC plan 2.0 agencies in Taiwan	LTC population: Older adults in 20 counties where LTC Plan 2.0 was being piloted in Taiwan in early 2017.<br><br>Study population: Interviews with 6 people working in 3 LTC Plan 2.0 Tier A agencies in north, west, and central Taiwan, including a chief executive officer (CEO) or executive director (ED) and a case manager at each agency.<br><br>Data source(s): Interviews	Medium
Dai et al. 2022(41)	China	LTCI pilot	Systematic literature review of LTC policies and implementation	This study examines the LTCI pilot in China and compares it to other LTCI programs in Germany, Japan, and South Korea through a systematic literature review and discusses the implications of its implementation in a national framework.	Adults in one of the 49 expanded pilot cities in China.<br><br>Data source(s): National and local government websites; China National Knowledge Infrastructure, Baidu Scholar and international databases	High
Feng et al. 2021(40)	China	LTCI pilot	Policy analysis	To provide a timely review and assessment of China's ongoing LTCI programs	People over 60 who meet the eligibility requirement for the LTCI pilot and who live in one of the 15 pilot cities.	High

For peer review only

Author Year	Country	Initiative	Design	Aim	Population and Data Sources	Quality Appraisal
				across the initial 15 pilot cities. The focus is on synthesizing key LTCI program features across all pilot sites, not on profiling pilot sites, and analyzing individual pilots in depth.	Data source(s): Policy documents; published research articles; grey literature; LTCI Policy Database, School of Public Health, Peking University	
Fong & Borowski 2022(51)	Singapore	CareShield Life	Policy analysis	To analyze the 2019 long-term care social insurance program, including successes and challenges, goals, design, features, financing arrangements, and initial assessment.	Older adults in Singapore. The proportion of those aged 65 and older increased x3 from 4.9% in 1980 to 14.5% in 2019.<br><br>Data source(s): Not reported. The reference list includes government documents and websites.	High
Han & Shen 2022(42)	China	LTCI pilot	Qualitative research	To explore the common problems in policy practices and to provide recommendations for further expansion of the LTCI pilot.	LTC population: China's adult population ≥60, which accounts for 18.7% of the total population.<br><br>Study population: n=10 participants, including managers (n=4), caregivers (n=2), a medical staff member (n=1), and older adults with disabilities (n=3)<br><br>Data source(s): In-depth interviews	High
Hashiguchi & Llena-Nozal 2020(32)	25 out of 41 OECD countries and EU Member States*	Public social protection systems for LTC	Comparative case study	To provide a novel set of comprehensive and internationally comparable estimates of the adequacy, equity and efficiency of public social protection systems for LTC in old age in OECD countries and EU Member States.	People 65 and older in the selected 25 OECD countries and EU Member States*.<br><br>Data source(s): Survey of Health, Ageing and Retirement in Europe (SHARE) Wave 7	N/A

For peer review only

Author Year	Country	Initiative	Design	Aim	Population and Data Sources	Quality Appraisal
Kotschy & Bloom 2022(33)	Germany, Israel, Japan, the Netherlands, South Korea	LTC systems comparison	Policy analysis	To investigate the challenges that population aging poses to long-term care.	Adults aged 65+ in Germany, Israel, Japan, the Netherlands, and South Korea<br><br>Data source(s): Survey of Health, Ageing and Retirement in Europe (SHARE) wave 7 and 8; Health and Retirement Study (HRS) wave 13; English Longitudinal Study of Ageing (ELSA) wave 8	N/A
Koumoutzis et al. 2021(49)	U.S.	Local initiatives to fund social care services	Qualitative research	To examine how communities across the nation are utilizing local funding streams to support aging services.	LTC population: Adults 60+ in the U.S.<br><br>Study population: NR<br><br>Data source(s): 2019 Advancing States national survey of State Units on Aging; telephone calls (State Units on Aging, Area Agencies on Aging, secretary of state's offices, county clerk's offices, and other officials involved in local funding efforts); web searches	Medium
Koumoutzis et al. 2022(48)	U.S.	Community LTC initiatives to support ageing	Survey	To study community initiatives developed to provide services to older adults through locally generated tax revenues and provide insight into their caregiver support efforts.	LTC population: Older adults in the U.S.<br><br>Study population: Representatives from 228 organizations across 15 states, delivering LTC services.<br><br>Data source(s): Phase 1: 2019 Advancing States national survey of state units on aging; telephone calls (telephone calls to state units on aging, area agencies on aging, aging services providers, county auditor offices, and Secretary of State offices); online reviews and web searches. Phase 2: Qualtrics survey to local funding organizations	High
Lei et al. 2022(34)	China	LTCI pilot	Quasi-experimental design	To evaluate the impact of LTCI on the covered populations in the LTCI pilots across different cities from 2015 to 2017, and to study	Older adults (aged 65+) from 152 cities: n=3,423<br>Treatment group (coverage): n=187<br>Control group (no coverage): n=3,236.	High

For peer review only

Author Year	Country	Initiative	Design	Aim	Population and Data Sources	Quality Appraisal
				various outcomes (including older adults' ADL-related need for care, family burden, medical expenditures, and some important health outcomes) among covered older adults and their families.	Data source(s): Chinese Longitudinal Healthy Longevity Survey (CLHLS)	
Liu & Hu 2022(46)	China	LTCI pilot	Quasi-experimental design	To investigate the impact of the policy pilot on the income fairness of the group system and to comprehensively analyze the institutional effect of the LTCI policy pilot.	People aged 60 and over living in one of the 15 LTCI pilot areas (n=25,063) included in CHARLS.<br><br>Data source(s): China Health and Retirement Longitudinal Study (CHARLS) database, 2013, 2015, 2018 (valid samples with a 3-year period)	High
Noda et al. 2021(55)	Japan, Korea, Thailand, China, Indonesia, the Philippines	Healthcare service delivery reform policies for the ageing	Policy comparison	To compare healthcare service delivery reform policies for older adults in Japan, Korea, Thailand, China, Indonesia and the Philippines.	LTC population: Older adults in Japan, Korea, Thailand, China, Indonesia, and the Philippines.<br><br>Study population: Health officials of provincial health and welfare departments, and service providers such as doctors and LTC providers (no n provided).<br><br>Data source(s): Document reviews; interviews with key informants	Medium
Peng et al. 2022(44)	China	LTCI pilot	Policy analysis	To assess the sustainability of the LTCI system in China using a policy population economics (PPE) system.	Population of 14 pilot cities in China. Due to a lack of data, one pilot city, Shihezi, was excluded.<br><br>Data source(s): Policy documents; China Statistical Yearbook 2020; the China City Statistical Yearbook; the Chinese National Economy and Social Development Statistics Bulletin	N/A

For peer review only

Author Year	Country	Initiative	Design	Aim	Population and Data Sources	Quality Appraisal
Sum et al. 2022(50)	Singapore	Integrated Patient-Centred Medical Home (PCMH) model	Quasi-experimental design	To address gaps in the literature on the evaluation of costs associated with the implementation of an integrated PCMH model for community-dwelling older adults with complex needs.	Patients with high biopsychosocial health risk, aged ≥40, who resided in Whampoa (n=165)<br><br>Data source(s): Client Service Receipt Inventory (CSRI) survey	High
Tang et al. 2022(45)	China	LTCI pilot	Quasi-experimental design	To evaluate the implementation effect of the Long-Term Care Insurance (LTCI) policy, and its impact on the medical expenses and the health status of the middle-aged and elder population.	Families with people middle-aged (≥45) and older in any of the 12 cities included in the CHARLS database (n=42,591)<br><br>Data source(s): China Health and Retirement Longitudinal Study (CHARLS)	Medium
Wu et al. 2020(39)	China	LTCI pilot	Retrospective cross-sectional study	To evaluate the LTCI pilot by exploring the characteristics and care needs of claimants, and performance of the assessment tool.	Claimants: n=4810; Individuals who received benefits: n=4582;<br>Aged ≥60 years: n=4357;<br>Beneficiaries without dementia: n=3791;<br>Beneficiaries with dementia: n=791;<br>Unsuccessful claimants without dementia: n=206;<br>Unsuccessful claimants with dementia: n=22<br><br>Data source(s): Claims data	Medium
Zhang & Yu 2019(37)	China	LTCI pilot	Quasi-experimental design	To explore the outcomes and evaluate the performance of the LTCI policy in the Chinese pilot cities and estimate the willingness of Chinese citizens to expand the formal implementation of LTCI policy in China.	Older adults engaged in the implementation process of the LTCI pilots in 15 pilot cities (n=1,167)<br>Elite politicians (n=5)<br><br>Data source(s): Survey questionnaire; interviews	Medium

**Author Year**	**Country**	**Initiative**	**Design**	**Aim**	**Population and Data Sources**	**Quality Appraisal**
Zhang et al. 2020(36)	China	LTCI pilot	Quasi-experimental design	To examine how the use of formal care impacts the use of informal care in Shanghai, which was one of China's first long-term insurance pilots in 2016.	Families in Shanghai that include an older adult (≥60) who had used formal care provided by LTCI for 1 to 3 months and a child who is primarily responsible for daily informal care.<br>Care recipients: n=407<br>Caregivers n=407<br><br>Data source(s): Interview-based surveys	Medium
Zhou & Dai 2021(38)	China	LTCI pilot	Systematic literature review of LTC policies	To review and assess the performance and effectiveness of the LTCI policy regime in China.	Older adults in one of the 15 pilot cities.<br><br>Data source(s): Policy documents	Medium

*The countries and subnational areas covered include Austria (Vienna region), Belgium (Flanders), Canada (Ontario), Croatia, Czech Republic, England, Estonia (Tallinn), Finland, France, Germany, Hungary, Iceland (Reykjavik), Ireland, Italy (South Tyrol), Japan, Korea, Latvia, Lithuania, Luxembourg, Slovak Republic, Slovenia, Spain, Sweden, the Netherlands, the United States of America (Illinois and California).
Quality appraisal (score % as per # of tool items): high quality=80-100%; medium quality=50-79%; low quality= <50%

For peer review only

Table 2: PROGRESS-Plus characteristics reported by study

Author	P	G/S	E	SE	SC	D	A	Author	P	G/S	E	SE	SC	D	A
Alders 2022 (52)	✓			✓		✓	✓	Koumoutzis 2021 (47)							
Beauregard 2021 (45)								Koumoutzis 2022 (46)							
Chen 2020 (33)	✓	✓	✓	✓	✓	✓	✓	Lei 2022 (32)	✓	✓		✓	✓		✓
Chen 2020 (51)	✓						✓	Liu 2022 (44)	✓	✓	✓	✓	✓	✓	✓
Chen 2022 (41)	✓	✓	✓	✓	✓	✓	✓	Noda 2021 (53)							
Chiu 2019 (50)								Peng 2022 (42)	✓					✓	✓
Dai 2022 (39)	✓						✓	Sum 2022 (48)	✓	✓	✓		✓	✓	✓
Feng 2021 (38)	✓						✓	Tang 2022 (43)		✓	✓	✓	✓	✓	✓
Fong 2022 (49)						✓	✓	Wu 2020 (37)	✓	✓				✓	✓
Han 2022 (40)	✓	✓				✓	✓	Zhang 2019 (35)	✓	✓		✓	✓	✓	✓
Hashiguchi 2020 (30)	✓			✓			✓	Zhang 2020 (34)	✓	✓	✓	✓	✓		✓
Kotschy 2022 (54)	✓					✓		Zhou 2021 (36)							

P=Place of residence; G=Gender/Sex; E=Education; SE=Socio-economic status; SC=Social capital; D=Disability; A=Age

For peer review only

For peer review only - http://bmjopen.bmj.com/site/about/guidelines.xhtml

Search Strategies

Search strategies are available via searchRxiv for the following information sources:

- MEDLINE: https://doi.org/10.1079/searchRxiv.2023.00214
- CINAHL: https://doi.org/10.1079/searchRxiv.2023.00213
- EconLit: https://doi.org/10.1079/searchRxiv.2023.00216
- Embase: https://doi.org/10.1079/searchRxiv.2023.00217
- ProQuest Dissertations and Theses: https://doi.org/10.1079/searchRxiv.2023.00220
- WHO ICTRP: https://doi.org/10.1079/searchRxiv.2023.00219
- ClinicalTrials.gov: https://doi.org/10.1079/searchRxiv.2023.00218

CADTH Grey Matters

All websites included in the Health Economics category of CADTH Grey Matters were searched or browsed for relevant records:

- Centre for Health Economics and Policy Analysis (McMaster University)
- Centre for Reviews and Dissemination (University of York)
- Economic Burden of Illness in Canada (Public Health Agency of Canada)
- Health Economics Research Unit (University of Aberdeen)
- IDEAS/RePEc database
- IHE publications (Institute of Health Economics, Canada)
- National Quality Measures Clearinghouse (Agency for Healthcare Research and Quality, U.S.)
- OHE publications (Office of Health Economics, United Kingdom)
- Ontario Case Costing Initiative (Ontario Ministry of Health and Long-Term Care)
- THETA Publications and Knowledge Translation to Policy (KT) Activities (Toronto Health Economics and Technology Assessment Collaborative)

If the website had a search feature, the search was as follows: ("long-term care" OR "long term care")

If there were too many results, it was narrowed by adding the following: AND (financing OR funding)

Results: 34 (38 minus 4 duplicates)

For peer review only

Data extraction form

General information

Study ID
Enter the Covidence record number here.

Example: #34

Title
Title of paper/abstract/report

Last name of lead author

Characteristics of study

Publication type
If publication type is "other" please specify the details below.
☐Journal article
☐Dissertation/thesis
☐Report
☐Other Click or tap here to enter text.

Aim/purpose/objective of study
Enter the research question or hypothesis.

Study design
Studies might not always explicitly state the design, in which case select the best option based on the methods section. If unclear, please include any relevant information in the notes section below.
☐Randomized controlled trial
☐Non-randomized experimental study
☐Quasi-experimental design

For peer review only

☐Cohort study
☐Cross-sectional study
☐Case–control study
☐Case series
☐Case report
☐Economic evaluation
☐Comparative case study
☐Policy analysis
☐Qualitative research (specific approach)
☐Other Click or tap here to enter text.

Country/setting
If the study is comparing countries/settings, ensure all the countries are listed, separated by semicolons. If the study does not mention the country but mentions a region, enter the region.

Population description
Describe the overall population. For more than one population start a new paragraph.

Gender
Enter the breakdown of population gender if reported.

Sexual orientation
Enter the breakdown of population sexuality if reported.

Race
Enter the breakdown of population racial composition if reported.

Age
Enter the overall age of population if reported. If age is reported as a range (e.g. 35–50 years) enter the relevant range and accompanying value with a colon in between (e.g. 60–79: 50%). If there are multiple ranges, separate using a semicolon (e.g. 60–79: 50%; 80–99: 50%).

Place of residence

For peer review only

Enter the breakdown of places of residence (e.g. rural, urban, suburban, long-term care [LTC] congregate setting, etc.) if reported.

Occupation
Enter the breakdown of occupations if reported.

Religious backgrounds
Enter the breakdown of religious backgrounds if reported.

Education
Enter the breakdown of educational backgrounds if reported.

Socioeconomic status
Enter the breakdown of socioeconomic status if reported.

Social capital
Social capital refers to social relationships, networks and social support. Enter information related to the social capital of the population if reported.

Disability
Enter the breakdown of the population disability categories if reported.

LTC financing
LTC system description
Describe the overall LTC system. For more than one system start a new paragraph.

Financing description
Describe how the LTC system is financed. For more than one system start a new paragraph.

Funding mechanism
Enter the details of the funding mechanism(s) in the relevant column. Enter NA in boxes where the funding mechanism is not applicable. Include the percentage mix for each funding mechanism if reported. If not reported, enter NR.

Funding mechanism	Details	Percentage mix
Guarantees		
Insurance		

For peer review only

Fees for service		
Loans		
Repayable contributions		
Result-based financing		
Equity		
Bonds		
Charitable donations		
Local government subsidies		
Taxes		
Co-payment by users		
Central government subsidies		
Other		

Service description
Describe the services covered under the programme policy reform or initiative if reported.

Coverage
How does the initiative/programme/policy/reform address LTC coverage? Enter NA if not addressed.

Quality
How does the initiative/programme/policy/reform address LTC quality? Enter NA if not addressed.

Financial protection
How does the initiative/programme/policy/reform address LTC financial protection? Enter NA if not addressed.

Financial sustainability
How does the initiative/program/policy/reform address LTC financial sustainability? Enter NA if not addressed.

Empirical evaluation
Intervention
What is the initiative/programme/policy/reform?

Comparator

For peer review only

What is the initiative/programme/policy/reform being compared to? If there is no comparator enter NA.

Intervention time frame
What is the time frame of the intervention?
Examples: policy put in place in June of 2017; pilot project from January 2017 to August 2019.

Data sources
Enter the sources of data. If there are multiple sources separate them with a semi-colon.

Dates of data collection
Enter the start and end date of data collection in yyyyy/mm format. If data sources are distinct years enter all years of data collection separated by commas.
Examples: 2017/11–2018/03; 2014; 2019; 2021.

Key findings and/or outcomes
Enter the key findings related to LTC financing.

Author's overall conclusion
Enter the authors' conclusions as it relates to LTC financing.

Reviewer notes
Enter any relevant notes or comments that are not captured elsewhere.

For peer review only

Preferred Reporting Items for Systematic reviews and Meta-Analyses extension for Scoping Reviews (PRISMA-ScR) Checklist

SECTION	ITEM	PRISMA-ScR CHECKLIST ITEM	REPORTED ON PAGE #
**TITLE**			
Title	1	Identify the report as a scoping review.	1
**ABSTRACT**			
Structured summary	2	Provide a structured summary that includes (as applicable): background, objectives, eligibility criteria, sources of evidence, charting methods, results, and conclusions that relate to the review questions and objectives.	2-3
**INTRODUCTION**			
Rationale	3	Describe the rationale for the review in the context of what is already known. Explain why the review questions/objectives lend themselves to a scoping review approach.	4-6
Objectives	4	Provide an explicit statement of the questions and objectives being addressed with reference to their key elements (e.g., population or participants, concepts, and context) or other relevant key elements used to conceptualize the review questions and/or objectives.	6
**METHODS**			
Protocol and registration	5	Indicate whether a review protocol exists; state if and where it can be accessed (e.g., a Web address); and if available, provide registration information, including the registration number.	Agreement of workplan provided to the contractor
Eligibility criteria	6	Specify characteristics of the sources of evidence used as eligibility criteria (e.g., years considered, language, and publication status), and provide a rationale.	7
Information sources*	7	Describe all information sources in the search (e.g., databases with dates of coverage and contact with authors to identify additional sources), as well as the date the most recent search was executed.	6
Search	8	Present the full electronic search strategy for at least 1 database, including any limits used, such that it could be repeated.	supplementary file
Selection of sources of evidence†	9	State the process for selecting sources of evidence (i.e., screening and eligibility) included in the scoping review.	7
Data charting process‡	10	Describe the methods of charting data from the included sources of evidence (e.g., calibrated forms or forms that have been tested by the team before their use, and whether data charting was done independently or in duplicate) and any processes for obtaining and confirming data from investigators.	7-8
Data items	11	List and define all variables for which data were sought and any assumptions and simplifications made.	7 and supplementary file
Critical appraisal of individual	12	If done, provide a rationale for conducting a critical appraisal of included sources of evidence;	8

For peer review only

SECTION	ITEM	PRISMA-ScR CHECKLIST ITEM	REPORTED ON PAGE #
sources of evidence§		describe the methods used and how this information was used in any data synthesis (if appropriate).	
Synthesis of results	13	Describe the methods of handling and summarizing the data that were charted.	8
**RESULTS**			
Selection of sources of evidence	14	Give numbers of sources of evidence screened, assessed for eligibility, and included in the review, with reasons for exclusions at each stage, ideally using a flow diagram.	Figure 1
Characteristics of sources of evidence	15	For each source of evidence, present characteristics for which data were charted and provide the citations.	8-9
Critical appraisal within sources of evidence	16	If done, present data on critical appraisal of included sources of evidence (see item 12).	Supplementary file, Table 1
Results of individual sources of evidence	17	For each included source of evidence, present the relevant data that were charted that relate to the review questions and objectives.	9-22
Synthesis of results	18	Summarize and/or present the charting results as they relate to the review questions and objectives.	9-22
**DISCUSSION**			
Summary of evidence	19	Summarize the main results (including an overview of concepts, themes, and types of evidence available), link to the review questions and objectives, and consider the relevance to key groups.	22-24
Limitations	20	Discuss the limitations of the scoping review process.	24
Conclusions	21	Provide a general interpretation of the results with respect to the review questions and objectives, as well as potential implications and/or next steps.	23-24
**FUNDING**			
Funding	22	Describe sources of funding for the included sources of evidence, as well as sources of funding for the scoping review. Describe the role of the funders of the scoping review.	32

JBI = Joanna Briggs Institute; PRISMA-ScR = Preferred Reporting Items for Systematic reviews and Meta-Analyses extension for Scoping Reviews.
* Where *sources of evidence* (see second footnote) are compiled from, such as bibliographic databases, social media platforms, and Web sites.
† A more inclusive/heterogeneous term used to account for the different types of evidence or data sources (e.g., quantitative and/or qualitative research, expert opinion, and policy documents) that may be eligible in a scoping review as opposed to only studies. This is not to be confused with *information sources* (see first footnote).
‡ The frameworks by Arksey and O'Malley (6) and Levac and colleagues (7) and the JBI guidance (4, 5) refer to the process of data extraction in a scoping review as data charting.
§ The process of systematically examining research evidence to assess its validity, results, and relevance before using it to inform a decision. This term is used for items 12 and 19 instead of "risk of bias" (which is more applicable to systematic reviews of interventions) to include and acknowledge the various sources of evidence that may be used in a scoping review (e.g., quantitative and/or qualitative research, expert opinion, and policy document).

From: Tricco AC, Lillie E, Zarin W, O'Brien KK, Colquhoun H, Levac D, et al. PRISMA Extension for Scoping Reviews (PRISMAScR): Checklist and Explanation. Ann Intern Med. 2018;169:467–473. doi: 10.7326/M18-0850.

For peer review only
